# Genome-Wide Analysis of DNA Methylation and Cigarette Smoking in a Chinese Population

**DOI:** 10.1289/ehp.1509834

**Published:** 2016-01-12

**Authors:** Xiaoyan Zhu, Jun Li, Siyun Deng, Kuai Yu, Xuezhen Liu, Qifei Deng, Huizhen Sun, Xiaomin Zhang, Meian He, Huan Guo, Weihong Chen, Jing Yuan, Bing Zhang, Dan Kuang, Xiaosheng He, Yansen Bai, Xu Han, Bing Liu, Xiaoliang Li, Liangle Yang, Haijing Jiang, Yizhi Zhang, Jie Hu, Longxian Cheng, Xiaoting Luo, Wenhua Mei, Zhiming Zhou, Shunchang Sun, Liyun Zhang, Chuanyao Liu, Yanjun Guo, Zhihong Zhang, Frank B. Hu, Liming Liang, Tangchun Wu

**Affiliations:** 1 Department of Occupational and Environmental Health, and; 2 Ministry of Education Key Lab for Environment and Health, School of Public Health, Tongji Medical College, Huazhong University of Science and Technology, Wuhan, China; 3 Department of Epidemiology, Harvard T.H. Chan School of Public Health, Boston, Massachusetts, USA; 4 Department of Cardiology, Union Hospital, Tongji Medical College, Huazhong University of Science and Technology, Wuhan, China; 5 Department of Cardiology, People’s Hospital of Zhuhai, Zhuhai, China; 6 Department of Cardiology, Bao’an Hospital, Shenzhen, China; 7 Department of Cardiology, Wuhan Central Hospital, Wuhan, China; 8 Department of Nutrition, Harvard T.H. Chan School of Public Health, Boston, Massachusetts, USA; 9 Channing Division of Network Medicine, Department of Medicine, Brigham and Women’s Hospital and Harvard Medical School, Boston, Massachusetts, USA; 10 Department of Biostatistics, Harvard T.H. Chan School of Public Health, Boston, Massachusetts, USA

## Abstract

**Background:**

Smoking is a risk factor for many human diseases. DNA methylation has been related to smoking, but genome-wide methylation data for smoking in Chinese populations is limited.

**Objectives:**

We aimed to investigate epigenome-wide methylation in relation to smoking in a Chinese population.

**Methods:**

We measured the methylation levels at > 485,000 CpG sites (CpGs) in DNA from leukocytes using a methylation array and conducted a genome-wide meta-analysis of DNA methylation and smoking in a total of 596 Chinese participants. We further evaluated the associations of smoking-related CpGs with internal polycyclic aromatic hydrocarbon (PAH) biomarkers and their correlations with the expression of corresponding genes.

**Results:**

We identified 318 CpGs whose methylation levels were associated with smoking at a genome-wide significance level (false discovery rate < 0.05), among which 161 CpGs annotated to 123 genes were not associated with smoking in recent studies of Europeans and African Americans. Of these smoking-related CpGs, methylation levels at 80 CpGs showed significant correlations with the expression of corresponding genes (including RUNX3, IL6R, PTAFR, ANKRD11, CEP135 and CDH23), and methylation at 15 CpGs was significantly associated with urinary 2-hydroxynaphthalene, the most representative internal monohydroxy-PAH biomarker for smoking.

**Conclusion:**

We identified DNA methylation markers associated with smoking in a Chinese population, including some markers that were also correlated with gene expression. Exposure to naphthalene, a byproduct of tobacco smoke, may contribute to smoking-related methylation.

**Citation:**

Zhu X, Li J, Deng S, Yu K, Liu X, Deng Q, Sun H, Zhang X, He M, Guo H, Chen W, Yuan J, Zhang B, Kuang D, He X, Bai Y, Han X, Liu B, Li X, Yang L, Jiang H, Zhang Y, Hu J, Cheng L, Luo X, Mei W, Zhou Z, Sun S, Zhang L, Liu C, Guo Y, Zhang Z, Hu FB, Liang L, Wu T. 2016. Genome-wide analysis of DNA methylation and cigarette smoking in Chinese. Environ Health Perspect 124:966–973; http://dx.doi.org/10.1289/ehp.1509834

## Introduction

Tobacco kills nearly 6 million people per year on account of direct tobacco use or indirect smoke exposure [World Health Organization (WHO) 2014]. Cigarette smoking, the primary method of tobacco consumption, is a major cause of preventable diseases (including cardiovascular diseases, respiratory diseases, and cancers) (Cunningham et al. 2014; Rea et al. 2002; Sosnowski and Przewoźniak 2015) and mortality (Ezzati and Lopez 2003; Mathers and Loncar 2006). Various human carcinogens have been identified in cigarette smoke, including polycyclic aromatic hydrocarbons (PAHs) [International Agency for Research on Cancer (IARC) 2004; Centers for Disease Control and Prevention (CDC) 2010; Rodgman et al. 2000]. Although the adverse health effects of smoking are well acknowledged, less is known about its underlying mechanisms of toxicity, especially at the molecular level.

DNA methylation is an epigenetic modification of the genome that is involved in regulating gene expression and genome stability (Lee and Pausova 2013). Methylation status can be modified by both genetic and environmental factors, and it can integrate the effects of both gene and environment on a phenotype or disease (Feil and Fraga 2012; Schadt 2009). Previous studies using targeted approaches (global methylation and candidate gene methylation) have established potential links between smoking and DNA methylation (Furniss et al. 2008; Philibert et al. 2010; Smith et al. 2007), but it was not until the widespread use of genome-wide methylation technologies that hundreds of smoking-related methylation markers were discovered and their relationships with smoking-related diseases were evaluated (Besingi and Johansson 2014; Breitling et al. 2011; Elliott et al. 2014; Harlid et al. 2014; Joubert et al. 2012; Markunas et al. 2014; Shenker et al. 2013; Sun et al. 2013; Zeilinger et al. 2013). Previous genome-wide methylation analyses of smoking have been conducted in Europeans (Guida et al. 2015; Shenker et al. 2013; Zeilinger et al. 2013) and African Americans (Dogan et al. 2014; Philibert et al. 2013; Sun et al. 2013); however, populations of mid-income countries such as China, the biggest cigarette producer and customer in the world, have not been evaluated.

To investigate epigenome-wide methylation alterations in relation to cigarette smoking in a Chinese population, we measured DNA methylation levels at > 485,000 CpG sites (CpGs) in peripheral blood leukocytes and conducted a genome-wide meta-analysis of DNA methylation and smoking in a total of 596 Chinese participants. Furthermore, we investigated the correlations of smoking-related CpGs with the expression of annotated genes as well as their associations with urinary monohydroxy-PAH (OH-PAH) metabolites.

## Methods

### Study Participants

In the present study, the genome-wide meta-analysis of DNA methylation and smoking was conducted in 596 Chinese participants selected from the Coke Oven Cohort, acute coronary syndrome (ACS) patients from Wuhan and Guangdong, China, and the Wuhan-Zhuhai (WHZH) Cohort (see Figure S1, for a flowchart of the study).

***The Coke Oven Cohort.*** A total of 1,628 coke-oven workers (COW) were recruited from a coke-oven plant in Wuhan, China in 2010 (Li et al. 2012). We included 144 workers in the present study based on the following criteria: *a*) donated blood and urine samples; *b*) had baseline total urinary OH-PAH (ΣOH-PAH) levels in the high tertile; *c*) had worked in the plant for more than 5 years; *d*) had no self-reported diseases or discomfort; *e*) had no fever or infectious conditions within 2 weeks of the baseline examination; *f*) did not take prescribed medicine in the past month; and *g*) had a body mass index (BMI) of 18.0–30.5. After quality controls for methylation and genotyping data were performed, 137 individuals (abbreviated as COW-1) remained in the present study.

***Acute coronary syndrome patients.*** The present study also included 103 clinically confirmed acute coronary syndrome (ACS) patients from Wuhan, China (recruited in Union Hospital and Wuhan Central Hospital), and 103 ACS patients from Guangdong, China (recruited in Bao’an Hospital and Peoples’ Hospital of Zhuhai). Patients were *a*) diagnosed with acute myocardial infarction or unstable angina pectoris by professional clinicians; *b*) did not have complications including congenital heart disease, cardiomyopathy, autoimmune disease, acute infection, tuberculosis, chronic obstructive pulmonary disease, diabetes mellitus, severe kidney or liver disease, hyperthyroidism, or malignant neoplasms; and *c*) donated blood samples at the earliest convenient time on the first day of admission. We included 101 patients from Wuhan (abbreviated as ACS-1) and 97 patients from Guangdong (abbreviated as ACS-2) who passed quality controls for both methylation data and genotyping data in the present analysis.

***The Wuhan–Zhuhai (WHZH) Cohort.*** The WHZH Cohort is a community-based cohort established in 2011 with 4,812 individuals (3,053 from Wuhan and 1,759 from Zhuhai, respectively) recruited at baseline (Song et al. 2014). From all participants who *a*) had no acute or chronic diseases or any kind of discomfort; *b*) showed no sign of abnormalities in clinical exanimations; *c*) had no fever or infectious conditions within 2 weeks of the baseline examination; *d*) did not take prescribed medicine in the past month; and *e*) donated both blood and urine samples, a total of 180 Wuhan residents were selected as healthy controls for the ACS patients in Wuhan (matched for age, sex, and BMI, *n* = 103) and/or healthy and low–PAH-exposed controls for COWs in Wuhan (matched for age, sex, and BMI, and with urinary ΣOH-PAH in the low tertile, *n* = 144; ACS patients and COWs shared 64 controls). A total of 103 Guangdong residents were selected as healthy controls for ACS patients from Guangdong (matched for age, sex, and BMI). We included 162 Wuhan residents and 99 Guangdong residents (abbreviated as WHZH) who passed quality controls for both methylation and genotyping data in the present analysis.

***Subjects for investigating methylation-expression correlations.*** To investigate the correlation between DNA methylation and gene expression, we recruited 144 individuals who participated in regular health examinations at the Health Examination Center of Dongfeng Central Hospital (Dongfeng Motor Corporation and Hubei University of Medicine) in Shiyan, China during April and May of 2015. The selected participants met the following criteria: *a*) were 20 to 70 years of age; *b*) had no self-reported diseases or discomfort; *c*) had no fever or infectious conditions within 2 weeks of the baseline examination; *d*) took no prescribed medicine in the past month; and *e*) donated both blood and urine samples. The methylation and expression data for all 144 subjects (abbreviated as SY) passed quality control and were included in the present analysis.

Our study was approved by the Ethics Committee of Tongji Medical College, and written informed consent was obtained from each participant. We required all participants to consume a bland diet and to fast for at least 12 hr before donating blood samples. Biological samples from all study panels were collected according to the same protocol and were stored under similar conditions.

### Laboratory Assays

***Illumina HumanMethylation450 BeadChip.*** Genomic DNA was extracted from whole blood using a BioTeke Whole Blood DNA Extraction Kit (BioTeke) and was then stored at –80°C. One microgram of each sample was bisulfite converted using a Zymo EZ DNA Methylation kit (Zymo Research) according to the manufacturer’s instructions and was then diluted to a concentration of 60 ng/μL. DNA methylation was assayed at > 485,000 CpGs using a HumanMethylation450 BeadChip (Illumina) with 4-μL bisulfite-converted samples.

***HumanHT-12 v4 Expression BeadChip.*** Leukocytes were isolated from whole blood immediately after blood collection, and the total RNA of blood leukocytes was isolated using TRIzol® LS solution (Invitrogen) according to the manufacturer’s instructions. Gene expression was profiled by a commercial company (ETMD, Beijing, China) using a HumanHT-12 v4 Expression BeadChip array according to standard protocols from Illumina. We acquired raw expression values using GenomeStudio (Illumina) and normalized the expression data using quantile-quantile normalization with the “beadarray” package (Dunning et al. 2007) in R 3.1.2 (R Core Team 2014). All unexpressed signals were assigned as 0 before analysis.

***Urinary creatinine and OH-PAH measurement.*** The urinary measures of creatinine and 12 OH-PAH metabolites in the WHZH (Song et al. 2014) and COW (Deng et al. 2014; Li et al. 2012) cohorts have been previously reported. All urine samples were collected in sterile conical tubes and were stored at –20°C until the laboratory assays were performed. The identification and quantification of PAH metabolites were based on retention time, mass-to-charge ratio, and peak area using a linear regression curve obtained from separate internal standard solutions. Among the 12 urinary OH-PAH metabolites, 10 noncarcinogenic metabolites, including 1-hydroxynaphthalene, 2-hydroxynaphthalene, 2-hydroxyfluorene, 9-hydroxyfluorene, 1-hydroxyphenanthrene, 2-hydroxyphenanthrene, 3-hydroxyphenanthrene, 4-hydroxyphenanthrene, 9-hydroxyphenanthrene, and 1-hydroxypyrene were above the limits of quantification (LOQ) and were hence included in the present analysis, whereas the 2 carcinogenic metabolites, 6-hydroxy chrysene and 3-hydroxy benzo[*a*]pyrene, were below the LOQ (Deng et al. 2014; Li et al. 2012) and, therefore, were not used in the present analysis. The OH-PAH levels were calibrated to urinary creatinine and were presented as micromoles per millimole creatinine.

### Quality Controls for Genome-Wide Data

We randomized sample pairs of cases (disease or exposed group) and matched controls across different plates and beadchips to minimize batch effects. We used the minfi package (Aryee et al. 2014) to preprocess the IDAT files. Signal outliers were identified by multidimensional scaling (MDS) analysis. We examined potential sample mix-ups by matching genotypes of the 65 single nucleotide polymorphisms (SNPs) on the Methylation450k Beadchips with the genotypes of the same SNPs obtained from the genome-wide association study (GWAS) data. Methylation probes were excluded if they: *a*) were the 65-SNP probes; *b*) had a missing rate > 20% across samples (missing was defined as follows for a probe of a certain sample: detection *p* value > 0.01 or bead counts < 3); or *c*) potentially contained or extended on SNPs with MAF > 0.05 in the 1000 Genomes Project 20110521 release for the ASN population, or cross-hybridized to other genomic locations (41,296 probes). Samples were excluded if they *a*) were MDS outliers; *b*) were mix-up samples; *c*) had a missing rate > 0.05 across probes; or *d*) failed GWAS quality controls, including unexpected duplicates or relatives (in IBD analysis, PI_HAT>0.185), sex discrepancies, heterozygosity outliers, or individual call rate < 0.98. After filtering, methylation values at 431,369 CpGs were normalized using the dasen method in the wateRmelon package (Pidsley et al. 2013). Methylation values with detection *p* value > 0.01 or bead counts < 3 were assigned as NA before further analysis.

### Statistical Analysis

***Genome-wide analyses of DNA methylation and smoking.*** Participants who had smoked an average of > 1 cigarette/day over the previous 6 months were defined as current smokers; participants who had stopped smoking for > 6 months were defined as former smokers; and participants who had never smoked during their lifetimes were classified as never smokers. Individuals who drank alcohol > 1 time/week over the previous 6 months were defined as current drinkers; individuals who had stopped drinking for > 6 months were defined as former drinkers; and individuals who had never had alcohol were defined as never drinkers. Surrogate variable analysis (SVA) was conducted separately in each panel using the SVA package (Leek et al. 2012). Variables used in the SVA included smoking status (coded as 0, 1, and 2 for never, former, and current smokers, respectively), age (years, as a continuous variable), sex (coded as 1 and 2 for male and female, respectively), drinking status (coded as 0, 1, and 2 for never, former, and current drinkers, respectively), and BMI (kilograms per meters squared, as a continuous variable). Surrogate variables (SVs) can capture major unknown variations of the genome-wide data that cannot be explained by included variables. Association analyses were performed separately in each panel using linear regression models, with inverse-normal transformed (INT) methylation beta values included as dependent variables and smoking status, age, sex, drinking status, BMI, and SVs included as independent variables. In the analyses of the COW and WHZH cohorts, ΣOH-PAHs were also included in the models as covariates because ΣOH-PAHs were considered in sample selection in these two panels. Results from all four panels were combined using a fixed effect meta-analysis with a sample-size weighted method to obtain *p* values and an inverse-variance weighted method to obtain estimates of effect size. The significance threshold for the genome-wide meta-analysis was a false discovery rate (FDR) < 0.05. The analyses were performed in R 3.1.2 (R Core Team 2014).

***Correlation between CpGs and gene expression.*** CpGs and expression probes were paired based on annotation files provided by Illumina, which provided information on genomic locations and gene annotations for both expression probes and CpGs probes. Linear regressions, of which dependent variables were inverse-normal–transformed expression values and independent variables were methylation values, age, and sex, were used to estimate associations between methylation and expression. For each CpG, the significance threshold was defined as 0.05/number of expression probes of the corresponding gene.

***Urinary PAH metabolites and smoking-related methylation alterations.*** We evaluated which urinary OH-PAHs could be used as representative biomarkers of smoking exposure by calculating the contribution of smoking to each OH-PAH metabolite [defined as the difference of *R* ^2^ between the models with and without smoking status; other covariates were age, drinking status, BMI, occupation, geographical region and beadchip operation date (geographical regions were coded as 1 and 2 for Wuhan and Guangdong, respectively)] using linear regression models in males from the WHZH cohort. The association between methylation values of the smoking-related CpGs and urinary 2-hydroxynaphthalene levels were analyzed seperately in males from the WHZH cohort and the Coke Oven cohort. Mediation analysis was performed to evaluate whether 2-hydroxynaphthalene showed mediation effects of smoking on methylation alterations in males from the WHZH cohort with adjustment for age, drinking status, BMI, occupation, differential leukocyte proportion, geographical region and beadchip operation date (Valeri and Vanderweele 2013). The association analyses were conducted in R 3.1.2 (R Core Team 2014), and the mediation analyses were performed in SAS 9.2 (SAS Institute Inc.).

## Results

### Basic Characteristics of the Participants

The genome-wide meta-analysis contained a total of 596 participants recruited from China, including 137 coke-oven workers (107 males; mean age = 46.51 years), 198 ACS patients (including 101 from Wuhan with 81 males and a mean age of 58.96 years, and 97 from Guangdong with 78 males and a mean age of 59.37 years), and 261 community residents from the WHZH cohort (206 males, mean age = 53.84 years). The characteristics of the study populations are summarized in Table 1.

**Table 1:** Characteristics of the study participants (*n* or mean ± SD).

Characteristics	Genome-wide meta-analysis of DNA methylation and smoking				Data set for methylation-expression association analysis
	COW-1 (*n* = 137)	ACS-1 (*n* = 101)	ACS-2 (*n* = 97)	WHZH (*n* = 261)	SY (*n* = 144)
Age, years	46.51 ± 8.91	58.96 ± 10.20	59.37 ± 11.47	53.84 ± 13.06	41.31 ± 10.27
Male, *n* (%)	107 (78.1%)	81 (80.2%)	78 (80.4%)	206 (78.9%)	107 (74.3%)
Smoking status, current/former/non	84/3/50	40/23/38	40/13/44	109/25/127	45/2/97
Drinking status, current/former/non	49/4/84	23/0/78	20/0/77	81/5/175	55/1/88
Body mass index, kg/m^2^	23.47 ± 2.72	24.84 ± 2.80	22.97 ± 2.43	23.37 ± 2.76	24.22 ± 2.67
White blood count, 10^9^/L	6.71 ± 1.51	7.31 ± 2.44	10.52 ± 3.82	6.00 ± 1.57	5.97 ± 1.40
Neutrophil proportions, %	59.62 ± 7.26	63.38 ± 10.50	72.50 ± 13.04	55.83 ± 8.33	57.97 ± 8.18
Lymphocyte proportions, %	36.83 ± 7.11	27.63 ± 9.73	19.07 ± 11.46	37.38 ± 8.17	33.68 ± 7.52
Intermediate cell^*a*^ proportions, %	3.55 ± 1.16	8.97 ± 3.08	8.44 ± 4.07	6.85 ± 3.75	8.35 ± 2.71
Abbreviations: ACS-1, ACS patients from Wuhan; ACS-2, ACS patients from Guangdong; COW-1, participants from the Coke Oven cohort; SY, individuals who attended regular health examinations in Shiyan; WHZH, residents selected from the Wuhan–Zhuhai cohort. ^***a***^Intermediate cells were defined as the sum of monocytes, eosinophils, and basophils.					

### Genome-Wide Analysis of DNA Methylation and Smoking

**Figure 1:**
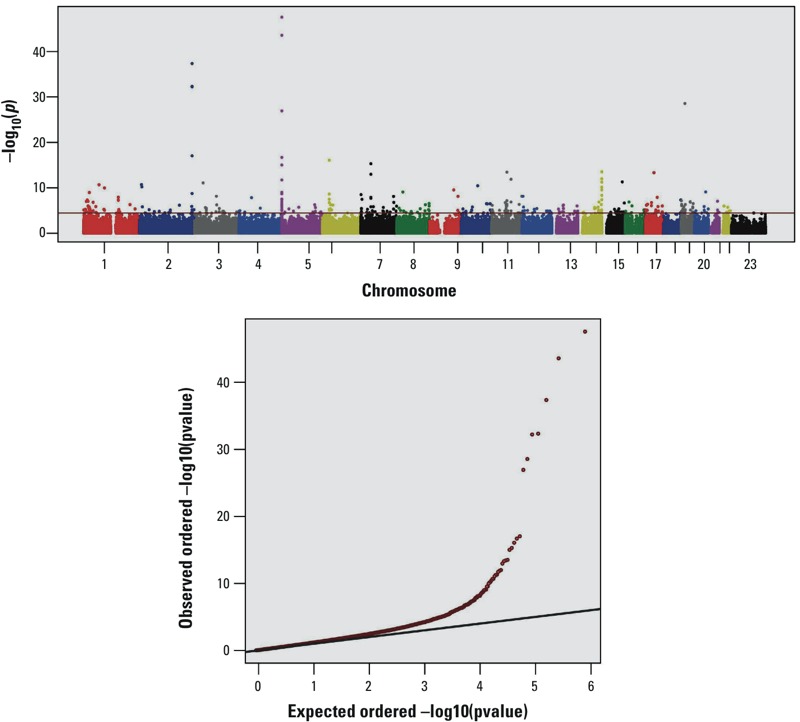
Manhattan plot and Q-Q plot of the *p* values of the associations between methylation and cigarette smoking in the genome-wide meta-analysis. In the Manhattan plot, the *x*-axis indicates genomic locations of the CpGs, the *y*-axis indicates –log10 (*p*-values) of the associations, and the red line indicates the –log (*p*-value) at false discovery rate (FDR) = 0.05. In the Q-Q plot, the *x*-axis shows the expected –log10 (*p*-values), whereas the *y*-axis indicates the observed –log10 (*p*-values).

In our genome-wide methylation meta-analysis, we identified 318 CpGs whose methylation levels were associated with smoking at genome-wide significance level (FDR < 0.05, Figure 1). Of these, 161 CpGs annotated to 123 genes were not reported to be significantly associated with smoking in previous genome-wide studies of methylation and smoking in Europeans (Guida et al. 2015; Shenker et al. 2013; Zeilinger et al. 2013) or in African Americans (Dogan et al. 2014; Philibert et al. 2013; Sun et al. 2013) (see Table S1). The association results for the top 40 smoking-related CpGs (FDR < 0.01) are presented in Table 2, and the association results of the 318 smoking-related CpGs in each panel are presented in Table S2. For most of the 318 CpGs, we observed a gradational alteration trend of the methylation levels from never to former to current smokers; the methylation alterations from current smokers to nonsmokers were larger than the alterations from former smokers to nonsmokers (see Table S3).

**Table 2:** The 40 CpGs associated with cigarette smoking in the genome-wide meta-analysis (FDR < 0.01).

Chr	Position	Gene	Relation to gene	CpG	Effect (s.e.)^*a*^	*p*	*FDR*
1	11908164	*NPPA*	TSS1500	cg05396397	0.257 (0.047)	1.02 × 10^–7^	6.97 × 10^–4^
1	19717337	*CAPZB*	Body	cg07573717	–0.104 (0.020)	1.00 × 10^–7^	6.97 × 10^–4^
1	21617442	*ECE1*	TSS1500	cg26348226	–0.143 (0.030)	2.18 × 10^–6^	0.007
1	42367407	*HIVEP3*	5’UTR	cg14663208	0.193 (0.035)	1.63 × 10^–7^	9.99 × 10^–4^
1	154299179	*ATP8B2*	TSS1500	cg06811467	–0.126 (0.021)	1.29 × 10^–8^	1.22 × 10^–4^
1	154379696	*IL6R*	Body	cg09257526	–0.112 (0.020)	5.59 × 10^–8^	4.16 × 10^–4^
2	11969958	*-*		cg02560388	–0.180 (0.037)	1.60 × 10^–6^	0.006
2	176987918	*HOXD9*	1stExon	cg22674699	0.216 (0.043)	7.15 × 10^–7^	0.003
2	231790037	*GPR55*	TSS200	cg16382047	–0.128 (0.026)	1.31 × 10^–6^	0.005
3	99792561	*C3orf26*	Body	cg15554421	–0.159 (0.031)	5.94 × 10^–7^	0.003
4	56813860	*CEP135*	TSS1500	cg26542660	–0.150 (0.026)	1.57 × 10^–8^	1.41 × 10^–4^
4	95679705	*BMPR1B*	5’UTR	cg09156233	0.198 (0.042)	3.15 × 10^–6^	0.010
5	146614298	*STK32A*	TSS1500	cg09088988	0.177 (0.035)	5.57 × 10^–7^	0.003
6	46702983	*PLA2G7*	1stExon	cg18630040	0.196 (0.038)	6.68 × 10^–7^	0.003
7	1102177	*C7orf50*	Body	cg15693483	–0.161 (0.027)	3.33 × 10^–9^	3.79 × 10^–5^
7	147065665	*MIR548I4*	Body	cg15700587	0.181 (0.034)	1.81 × 10^–7^	0.001
7	158937969	*VIPR2*	TSS1500	cg23572908	0.239 (0.048)	1.16 × 10^–6^	0.004
9	127054428	*NEK6*	TSS1500	cg14556677	–0.111 (0.019)	7.99 × 10^–9^	8.02 × 10^–5^
10	49892930	*WDFY4*	TSS1500	cg15164194	–0.091 (0.020)	1.81 × 10^–6^	0.006
10	116298339	*ABLIM1*	Body	cg07978738	–0.145 (0.028)	3.62 × 10^–7^	0.002
10	128994432	*DOCK1*	Body	cg03242819	0.229 (0.044)	3.41 × 10^–7^	0.002
11	65201834	*-*		cg10416861	0.216 (0.043)	8.67 × 10^–7^	0.004
11	65550444	*-*		cg09419102	–0.141 (0.029)	1.60 × 10^–6^	0.006
11	122709551	*CRTAM*	Body	cg22512531	0.138 (0.026)	4.70 × 10^–7^	0.002
12	7055657	*PTPN6*	TSS200	cg23193870	–0.204 (0.042)	1.84 × 10^–6^	0.006
14	78051204	*SPTLC2*	Body	cg14544289	–0.199 (0.041)	3.06 × 10^–6^	0.010
14	89933549	*FOXN3*	5’UTR	cg13679772	0.149 (0.031)	1.94 × 10^–6^	0.007
14	106331803	*-*		cg14387626	–0.142 (0.026)	4.34 × 10^–8^	3.47 × 10^–4^
14	106354912	*-*		cg27113548	–0.284 (0.047)	4.60 × 10^–9^	5.09 × 10^–5^
15	99194021	*IGF1R*	Body	cg07779120	0.251 (0.046)	2.35 × 10^–7^	0.001
17	4923126	*KIF1C*	Body	cg03877174	0.154 (0.030)	7.12 × 10^–7^	0.003
17	9921982	*GAS7*	Body	cg02018337	–0.122 (0.024)	4.43 × 10^–7^	0.002
17	27050723	*RPL23A*	Body	cg18150958	–0.229 (0.046)	8.85 × 10^–7^	0.004
17	27401793	*TIAF1*	5’UTR	cg18960216	0.181 (0.034)	1.97 × 10^–7^	0.001
17	56082867	*SFRS1*	3’UTR	cg08591265	–0.152 (0.032)	1.42 × 10^–6^	0.005
18	76739409	*SALL3*	TSS1500	cg05080154	0.212 (0.038)	4.87 × 10^–8^	3.75 × 10^–4^
19	40919465	*PRX*	TSS200	cg01447828	0.233 (0.044)	1.54 × 10^–7^	9.63 × 10^–4^
19	53758055	*ZNF677*	5’UTR	cg03217253	0.216 (0.042)	3.10 × 10^–7^	0.002
20	43438809	*RIMS4*	Body	cg15207742	0.212 (0.043)	1.40 × 10^–6^	0.005
22	20792535	SCARF2	TSS1500	cg14785479	0.123 (0.025)	1.10 × 10^–6^	0.004
Abbreviations: Body, gene body; Chr, Chromosome; *FDR*, false discovery rate; TSS200, within 200 bps from transcription start site; TSS1500, within 1,500 bps from transcription start site; UTR, untranslated regions. ^***a***^Estimates were calculated based on inverse-normal transformed methylation values. Fixed effect meta-analysis was used with a sample size–weighted method to obtain *p* values and with an inverse-variance weighted method to obtain estimates of effect size.							

### Correlations with the Expression of Annotated Genes

We further investigated whether the methylation values of the smoking-related CpGs were correlated with the expression of corresponding genes in an independent set of 144 healthy individuals whose methylome and gene-expression profiles were both measured (Table 1). Seventy-seven of the 318 smoking-related CpGs were excluded from the analysis either because no expression probes were designed for the genes or because of the low expression rate in blood leukocytes. Of the remaining 241 CpGs (a total of 414 CpG-expression probe pairs) that had qualified expression data for the annotated genes, we observed that methylation levels at 80 CpGs were associated with the expression of their corresponding genes (*p* < 0.05/number of expression probes of the corresponding gene; e.g., on the body of *RUNX3*, *p* = 1.57 × 10^–7^ for cg10951873 and ILMN_1787461; on the body of *IL6R*, *p* = 1.98 × 10^–9^ for cg09257526 and ILMN_1696394, *p* = 5.61 × 10^–6^ for cg09257526 and ILMN_1754753; within 1,500 bps from the transcription start site of *CEP135*, *p* = 1.82 × 10^–2^ for cg26542660 and ILMN_1693766; on the body of *CDH23*, *p* = 9.45 × 10^–3^ for cg10750182 and ILMN_1779934; within 1,500 bps from the transcription start site of *PTAFR*, *p* = 2.07 × 10^–16^ for cg20460771 and ILMN_1746836; in the 5´ untranslated regions of *ANKRD11*, *p* = 1.03 × 10^–8^ for cg01107178 and ILMN_2108709) (see Table S4).

### Associations of Smoking-Related CpGs and Urinary 2-Hydroxynaphthalene

Because the majority of smokers in our study were males (98.52%) and to avoid effects owing to occupational exposures, the analysis was mainly conducted in males from the WHZH cohort. We first tested which OH-PAH metabolite was the most representative biomarker for smoking. We observed that smoking could account for 18.0% of the variation of urinary 2-hydroxynaphthalene, larger than the variations explained by smoking for the other 9 OH-PAH metabolites (see Table S5).

**Figure 2:**
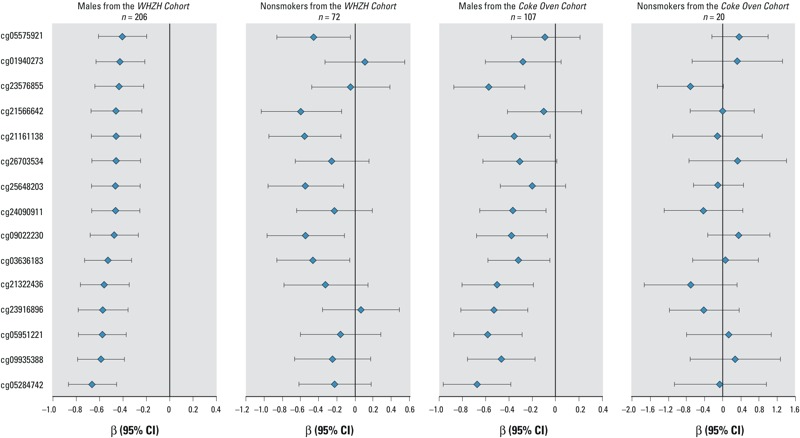
Associations of the 15 smoking-related CpGs and urinary 2-hydroxynaphthalene levels in males from the Wuhan–Zhuhai (WHZH) cohort and the Coke Oven cohort.

We then assessed the association between methylation levels at the 318 smoking-related CpGs and urinary 2-hydroxynaphthalene levels (see Table S6) and found 15 significant associations after performing Bonferroni corrections (*p* < 1.57 × 10^–4^) (Figure 2). When restricting the analysis only to nonsmokers, these associations were greatly attenuated (Figure 2; see also Table S6), suggesting that the correlations between DNA methylation and urinary 2-hydroxynaphthalene were mainly attributable to smoking. We further investigated whether 2-hydroxynaphthalene could be a mediator of these smoking-induced methylation alterations and found that among the 15 CpGs associated with 2-hydroxynaphthalene, the smoking-related methylation variation at 12 CpGs (including cg05575921, cg23916896, cg24090911, and cg26703534 on *AHRR*) might be partially mediated by their associations with urinary 2-hydroxynaphthalene levels (*p* < 0.05) (Table 3).

**Table 3:** Mediation analysis of 15 significant CpGs whose methylation levels were correlated with urinary 2-hydroxynaphthalene in males from the Wuhan–Zhuhai (WHZH) cohort.

CpG	Gene	Relationships between smoking, 2-hydroxynaphthalene, and methylation			
		Total effect of smoking on methylation^*a*^		Mediating effect of 2-hydroxynaphthalene in association between smoking and methylation^*a*^	
		Total effect (s.e.)	*p*	Indirect effect (s.e.)	*p*
cg05575921	*AHRR*	–0.667 (0.060)	2.17 × 10^–28^	–0.084 (0.032)	0.008
cg21161138	*AHRR*	–0.305 (0.074)	< 1.00 × 10^–05^	–0.134 (0.041)	0.059
cg23916896	*AHRR*	–0.085 (0.078)	< 1.00 × 10^–05^	–0.136 (0.042)	0.017
cg23576855	*AHRR*	–0.655 (0.059)	<1.00 × 10^–05^	–0.056 (0.030)	0.100
cg24090911	*AHRR*	–0.203 (0.074)	1.81 × 10^–10^	–0.124 (0.040)	0.009
cg25648203	*AHRR*	–0.360 (0.071)	< 1.00 × 10^–05^	–0.089 (0.037)	0.088
cg26703534	*AHRR*	–0.428 (0.067)	1.10 × 10^–17^	–0.092 (0.035)	0.008
cg01940273	*-*	–0.542 (0.063)	< 1.00 × 10^–05^	–0.088 (0.033)	0.016
cg03636183	*F2RL3*	–0.464 (0.068)	3.70 × 10^–05^	–0.059 (0.034)	0.001
cg05284742	*ITPK1*	–0.273 (0.070)	0.273	–0.084 (0.036)	0.001
cg05951221	*-*	–0.430 (0.068)	1.39 × 10^–04^	–0.057 (0.035)	0.015
cg09022230	*TNRC18*	–0.282 (0.073)	1.24 × 10^–04^	–0.103 (0.039)	0.008
cg09935388	*GFI1*	–0.274 (0.072)	0.006	–0.091 (0.037)	0.002
cg21322436	*CNTNAP2*	–0.284 (0.073)	9.90 × 10^–05^	–0.102 (0.038)	0.008
cg21566642	-	–0.534 (0.067)	1.05 × 10^–04^	–0.083 (0.035)	0.021
^***a***^Adjusted for age, drinking status, body mass index, occupation, differential leukocyte proportions, geographical region, and beadchip operation date in a mediation macro in SAS 9.2 (SAS Institute Inc.): %macro mediation (data=, yvar=, avar=, mvar=, cvar=, a0=, a1=, m=, nc=, yreg=, mreg=, Interaction=, casecontrol= false, output= reduced, c=, boot=) (Valeri and Vanderweele 2013). Methylation values were inverse-normal transformed, and concentrations of urinary 2-hydroxynaphthalene were natural log–transformed.					

Although subjects from the Coke Oven cohort had occupational exposures to PAHs, similar association patterns between smoking, 2-hydroxynaphthalene, and methylation at these CpGs were observed in male subjects from the Coke Oven cohort after adjustment for 1-hydroxypyrene, an occupational exposure marker for coke-oven workers (Figure 2; see also Table S6).

## Discussion

In the present study, we identified 318 smoking-related CpGs through a genome-wide meta-analysis of DNA methylation in several Chinese populations. Among the identified CpGs, 161 annotated to 123 genes were not associated with smoking in recent studies of Europeans (Guida et al. 2015; Shenker et al. 2013; Zeilinger et al. 2013) or African Americans (Dogan et al. 2014; Philibert et al. 2013; Sun et al. 2013). We also observed that methylation levels at some smoking-related CpGs might affect the expression of corresponding genes, and some smoking-related methylation alterations might be partly mediated by exposure to naphthalene.

Although China is the largest consumer and producer of tobacco in the world (Gu et al. 2009), genome-wide methylation studies of DNA methylation and smoking have not been conducted in Chinese populations. The present study identified 318 smoking-related CpGs in a Chinese population, 157 of which have been reported by previous methylation studies, suggesting that smoking-related methylation alterations were mainly consistent across Chinese and Western populations. The 161 CpGs that have not been previously reported in Europeans or African Americans suggest novel smoking-related sites or sites specific to the Chinese population, which calls for replication by further studies among other Chinese populations. Most of the identified loci were annotated on genes involved in the metabolism of smoking-released chemicals [e.g., *AHRR* is a repressor of the nuclear receptor for aryl hydrocarbons that is involved in xenobiotic metabolism (Shenker et al. 2013)] or that might be involved in smoking-related health effects [e.g., methylation of *F2RL3* mediates the detrimental impacts of smoking and is related to mortality caused by coronary heart disease (Breitling et al. 2012; Zhang et al. 2014)].

DNA methylation might be a potential link between smoking and human diseases. In the present study, the smoking-related methylation changes to *RUNX3*, *IL6R*, *PTAFR*, and *ANKRD11* (cardiovascular-related genes) and *CEP135* and *CDH23* (cancer-related genes) corresponded to increased gene expression. *RUNX3* encodes a member of the runt domain-containing family of transcription factors, which might have important functions in innate and adaptive immune cell types and might be associated with several inflammatory-related diseases (Lotem et al. 2015). Interleukin 6 is a cytokine with vital roles in inflammatory responses, and its dysregulation has been implicated in many health problems (Ferreira et al. 2013). *PTAFR* encodes a receptor for platelet-activating factor (PAF) that plays a significant role in proinflammatory processes (Ninio et al. 2004). In addition to their critical role in hemostasis and thrombosis, platelets are also involved in regulating inflammatory and immune responses (von Hundelshausen and Weber 2007). *ANKRD11* might be involved in apoptosis pathways (e.g., p53 signaling) (Lim et al. 2012; Neilsen et al. 2008), which have been reported to play key roles in the pathogenesis of cardiovascular diseases (Lee and Gustafsson 2009). It has been speculated that smoking-induced abnormal physiological processes might be important mechanisms in the development of cardiovascular diseases (Frostegård 2013). Our findings that smoking was associated with methylation of cardiovascular-related genes that were correlated with the corresponding expression suggested that DNA methylation might contribute to disease progression though immune reactions, inflammation responses, and apoptosis induced by smoking.

*CEP135* encodes a centrosomal protein that acts as a scaffolding protein during early centriole biogenesis (Kim et al. 2008). Centrosomes play crucial roles in many processes (including organizing mitotic spindle poles), and centrosome aberrations (Nigg 2002) are included in many human tumors (Rusan and Peifer 2007). Notably, antimitotic compounds have been identified in tobacco-smoke condensate, and smoking could induce mitotic abnormalities (Qiao et al. 2003; Vogt Isaksen 2004). *CDH23* encodes cadherin 23, which acts as a mediator at intercellular junctions and in cellular differentiation and cell migration (Agarwal 2014). Previous studies showed that *CDH23* was up-regulated in breast cancer tissues and was involved in metastatic processes (Binai et al. 2013). Recent evidence has suggested that active smoking played a potentially causal role in breast cancer (Reynolds et al. 2009). Smoking is also a well-established cause of many cancers (e.g., lung, colon, and stomach) (Gandini et al. 2008). Therefore, it is possible that methylation alterations are potential mechanisms of smoking-induced adverse effects and cancers.

Cigarette smoking is a major source of PAH exposure, particularly naphthalene exposure (Ding et al. 2005; Jacob et al. 2013). We estimated that cigarette smoking accounted for 18.0% of the variation in urinary 2-hydroxynaphthalene among males in the WHZH cohort, which supports further investigation of urinary 2-hydroxynaphthalene as a possible biomarker of internal exposure to smoking-sourced PAHs. Smoking-related alterations of *AHRR* methylation might be caused by exposure to PAHs (Shenker et al. 2013). *AHRR* encodes a repressor of the aryl hydrocarbon receptor (AhR) (Harlid et al. 2014). Previous studies have suggested that the AhR pathway is important in the metabolism of various xenobiotics including PAHs (Zeilinger et al. 2013) and is modified in response to exposure to smoking (Besingi and Johansson 2014). Our present data suggested that smoking-released naphthalene might alter the AhR pathway by changing the methylation levels of vital genes in the AhR pathway.

Different cells and tissues have distinct DNA methylation signatures (Ohgane et al. 2008). The use of peripheral blood DNA in the present study is reasonable for two reasons. First, peripheral blood is an important carrier for many xenobiotics absorbed into human bodies (Barr et al. 2007); peripheral blood cells have direct contact with the internal forms of xenobiotics and react to them (Bonassi et al. 2007). Second, blood samples are the most convenient to collect in large-scale studies, and using blood cells allows comparison of our results with those of other studies. A limitation of using blood leukocytes as the source of DNA for methylation analyses is that methylation varies among leukocyte subtypes, and the distribution of leukocyte subtypes may vary in association with exposure, thus resulting in potential confounding of associations between exposures and methylation (Reinius et al. 2012). As suggested in a previous study that factor-based “batch” correction methodology (such as surrogate variable analysis) can not only control for batch effects but can also empirically estimate and control for cell-type compositions (Jaffe and Irizarry 2014), we adopted surrogate variables in our genome-wide methylation analyses to limit effects of batch and cellular compositions simultaneously. When investigating associations between smoking-related CpGs and urinary 2-hydroxynaphthalene, we adjusted for differential white blood cell proportions in the analysis models. However, we cannot rule out the potential for residual confounding related to leukocyte subtype variations or to other factors. In addition, given the cross-sectional study design, we could not establish a temporal relationship between smoking and DNA methylation.

## Conclusions

On the basis of a genome-wide analysis of smoking and DNA methylation in a Chinese population, we identified 318 smoking-related CpGs, among which 161 CpGs annotated to 123 genes have not been previously reported in Europeans or in African Americans. Some smoking-related CpGs might play a role in gene regulation. We also found that naphthalene might be one of the smoking-released chemicals inducing the methylation alterations that we observed in smokers. Additional studies are needed to replicate our findings, to determine their potential relevance to health outcomes, and to elucidate underlying mechanisms that link smoking and DNA methylation.

## Supplemental Material

(2.9 MB) PDF
