# Supplementary material for: Genome-Wide Analysis of DNA Methylation and Cigarette Smoking in a Chinese Population
Source: Environ Health Perspect. 2016 Jan 12;124(7):966–73. doi: 10.1289/ehp.1509834 (PMC4937856; doi:10.1289/ehp.1509834)
Supplement: (2.9 MB) PDF [file ehp.1509834.s001.acco.pdf]

Environ Health Perspect
DOI: 10.1289/ehp.1509834

Note to readers with disabilities: *EHP* strives to ensure that all journal content is accessible to all readers. However, some figures and Supplemental Material published in *EHP* articles may not conform to 508 standards due to the complexity of the information being presented. If you need assistance accessing journal content, please contact ehp508@niehs.nih.gov. Our staff will work with you to assess and meet your accessibility needs within 3 working days.

Supplemental Material

Genome-Wide Analysis of DNA Methylation and Cigarette Smoking in Chinese

Xiaoyan Zhu, Jun Li, Siyun Deng, Kuai Yu, Xuezhen Liu, Qifei Deng, Huizhen Sun, Xiaomin Zhang, Meian He, Huan Guo, Weihong Chen, Jing Yuan, Bing Zhang, Dan Kuang, Xiaosheng He, Yansen Bai, Xu Han, Bing Liu, Xiaoliang Li, Liangle Yang, Haijing Jiang, Yizhi Zhang, Jie Hu, Longxian Cheng, Xiaoting Luo, Wenhua Mei, Zhiming Zhou, Shunchang Sun, Liyun Zhang, Chuanyao Liu, Yanjun Guo, Zhihong Zhang, Frank B. Hu, Liming Liang, and Tangchun Wu

Table of Contents

Table S1. The 318 identified CpGs associated with cigarette smoking in the genome-wide meta-analysis (*FDR* < 0.05).

Table S2. The effect size (s.e.) of smoking on the 318 CpGs with inverse normal transformed methylation values calculated in different groups.

Table S3. The median methylation values (inter quartile range) of the 318 smoking-related CpGs in never, former and current smokers.

Table S4. Methylation-gene expression correlations of the smoking-related CpGs in SY (*n* = 144).

Table S5. Contribution rates of smoking on urinary PAHs metabolites calculated in male individuals from the WHZH cohort (*n* = 206).

Table S6. Associations between the smoking-related CpGs and urinary 2-hydroxynaphthalene levels in males from the WHZH Cohort and the Coke Oven Cohort.
Figure S1. The flowchart of the study.
References

Table S1. The 318 identified CpGs associated with cigarette smoking in the genome-wide meta-analysis (*FDR* < 0.05).

Chr	Position	Gene	Relation to gene	CpG	Our study (*n*=596)[a]		In Europeans (*n*=745)[b]		In African Americans (*n*=972)[c]	
					Effect	*p*	Effect	*p*[*]	Effect	*p*[#]
1	1078901	-	-	cg19708306[d]	0.136	7.89E-06		-		-
1	8212843	-	-	cg05270224[d]	0.196	5.70E-06		-		-
1	11865352	*CLCN6*	TSS1500	cg05228408	-0.152	1.79E-05	-0.090	4.00E-08	-0.020	2.44E-06
1	11908164	*NPPA*	TSS1500	cg05396397[d]	0.257	1.02E-07		-		-
1	16301562	*ZBTB17*	5'UTR	cg04211179	-0.145	5.11E-08		-		-
1	19717337	*CAPZB*	Body	cg07573717[d]	-0.104	1.00E-07		-		-
1	19810690	*CAPZB*	Body	cg19713429	-0.175	1.05E-06	-0.172	1.46E-09		-
1	21617442	*ECE1*	TSS1500	cg26348226[d]	-0.143	2.18E-06		-		-
1	24867657	-	-	cg17094249[d]	-0.229	6.34E-06		-		-
1	25173236	-	-	cg02818189[d]	-0.135	1.85E-05		-		-
1	25254746	*RUNX3*	Body	cg10951873	-0.186	1.85E-06		-		-
1	25349681	-	-	cg27537125	-0.141	1.11E-09	-0.207	2.55E-26		-
1	28521540	*PTAFR*	TSS1500	cg20460771[d]	0.133	5.47E-06		-		-
1	31234437	-	-	cg25310233[d]	-0.125	1.87E-05		-		-
1	42367407	*HIVEP3*	5'UTR	cg14663208[d]	0.193	1.63E-07		-		-
1	42385662	*HIVEP3*	TSS1500	cg16145216[d]	0.197	1.12E-05		-		-
1	54122060	*GLIS1*	5'UTR	cg24741609	-0.181	1.39E-06		-		-
1	54946918	-	-	cg04158878[d]	0.147	2.86E-05		-		-
1	68299493	*GNG12*	TSS1500	cg25189904	-0.296	2.25E-11	-0.559	1.25E-32		-
1	90289611	*LRRC8D*	5'UTR	cg20146909	-0.185	5.88E-06		-		-
1	92946825	*GFI1*	Body	cg12876356	-0.181	8.95E-06	-0.415	1.63E-17		-
1	92947588	*GFI1*	Body	cg09935388	-0.289	1.11E-10	-0.669	9.56E-33		-
1	153746211	*INTS3*	3'UTR	cg08129092[d]	0.120	2.52E-05		-		-
1	154298956	*ATP8B2*	TSS1500	cg23924887[d]	-0.110	2.44E-05		-		-
1	154299179	*ATP8B2*	TSS1500	cg06811467[d]	-0.126	1.29E-08		-		-
1	154379696	*IL6R*	Body	cg09257526[d]	-0.112	5.59E-08		-		-
1	156074135	-	-	cg12593793	-0.110	1.32E-05	-0.120	3.28E-10		-

1	156710278	*MRPL24*	5'UTR	cg01416295[d]	-0.180	6.53E-06	-		-
1	159753902	-	-	cg09471611[d]	0.144	1.84E-05	-		-
1	206224334	*AVPR1B*	5'UTR	cg08709672	-0.235	5.08E-07	-0.189	4.75E-25	-
1	228689056	-	-	cg11229399[d]	0.194	4.75E-06	-		-
2	8343710	-	-	cg23079012	-0.278	2.07E-11	-		-
2	9843525	-	-	cg08035323	0.261	6.31E-11	-		-
2	11969958	-	-	cg02560388[d]	-0.180	1.60E-06	-		-
2	42071660	-	-	cg13711966[d]	0.168	6.76E-06	-		-
2	65284262	*CEP68*	5'UTR	cg05010058[d]	-0.181	1.78E-05	-		-
2	87088834	*CD8B*	Body	cg15746583[d]	0.094	1.59E-05	-		-
2	112124380	-	-	cg09570614	0.139	3.57E-05	-		-
2	129231478	-	-	cg01765406	-0.120	3.16E-05	-0.111	6.80E-11	-
2	135926634	*RAB3GAP1*	3'UTR	cg20949306[d]	0.180	2.71E-05	-		-
2	136577303	*LCT*	Body	cg14667406[d]	-0.212	8.71E-06	-		-
2	176987918	*HOXD9*	1stExon	cg22674699[d]	0.216	7.15E-07	-		-
2	231790037	*GPR55*	TSS200	cg16382047[d]	-0.128	1.31E-06	-		-
2	231790777	*GPR55*	TSS1500	cg19827923	-0.161	1.33E-06	-0.131	1.27E-11	-
2	233283329	-	-	cg03329539	-0.312	9.46E-18	-0.348	6.51E-35	-
2	233284112	-	-	cg06644428	-0.255	1.86E-09	-0.708	8.48E-28	-
2	233284402	-	-	cg05951221	-0.490	6.13E-33	-0.627	7.52E-69	-
2	233284661	-	-	cg21566642	-0.588	4.35E-38	-0.918	2.14E-79	-
2	233284934	-	-	cg01940273	-0.539	4.56E-33	-0.613	7.44E-74	-
2	233285289	-	-	cg13193840	-0.230	7.42E-06	-0.293	6.96E-16	-
2	233320593	*ALPI*	TSS1500	cg13481776[d]	0.131	2.17E-05	-		-
2	241140248	-	-	cg12756150[d]	0.201	6.94E-06	-		-
3	24725365	-	-	cg13279811[d]	-0.176	3.06E-05	-		-
3	39193251	*CSRNP1*	5'UTR	cg00501876	-0.265	8.61E-12	-0.141	1.03E-16	-
3	49394622	*GPX1*	3'UTR	cg18642234	-0.196	2.35E-06	-0.106	6.82E-12	-
3	52812520	*ITIH1*	TSS200	cg19784816[d]	0.133	3.24E-05	-		-
3	53700141	*CACNA1D*	Body	cg15417641	0.221	3.42E-06	-		-
3	53700195	*CACNA1D*	Body	cg00336149	0.208	3.68E-05	-		-
3	53700263	*CACNA1D*	Body	cg21188533	0.226	7.81E-06	-		-

3	96533520	*EPHA6*	1stExon	cg09301294[d]	0.159	1.96E-05	-		-	
3	98251294	*GPR15*	1stExon	cg19859270	-0.279	7.50E-09	-0.340	8.57E-27	-0.021	2.45E-11
3	99792561	*C3orf26*	Body	cg15554421[d]	-0.159	5.94E-07	-		-	
3	111260756	*CD96*	TSS200	cg05655806	-0.196	3.71E-06	-0.224	3.84E-09	-	
3	111260783	*CD96*	TSS200	cg04039397	-0.204	1.98E-05	-		-	
3	126242973	*CHST13*	TSS1500	cg18165852[d]	0.187	1.65E-05	-		-	
3	130236259	-	-	cg26958735[d]	0.176	1.57E-05	-		-	
3	188425256	*LPP*	Body	cg25853622[d]	-0.061	2.12E-05	-		-	
3	194119885	*GP5*	5'UTR	cg13185177	0.135	2.45E-05	-		-	
4	2460483	-	-	cg23867146[d]	0.194	1.34E-05	-		-	
4	5053341	*STK32B*	TSS200	cg10351287[d]	0.193	1.11E-05	-		-	
4	26789915	-	-	cg19719391	0.150	2.32E-05	-		-	
4	56813860	*CEP135*	TSS1500	cg26542660[d]	-0.150	1.57E-08	-		-	
4	95679705	*BMPR1B*	5'UTR	cg09156233[d]	0.198	3.15E-06	-		-	
5	321320	*AHRR*	Body	cg11554391	-0.217	4.03E-07	-0.201	5.91E-12	-	
5	323794	*AHRR*	Body	cg17924476	0.173	7.64E-07	-		-	
5	344057	*AHRR*	Body	cg09338136	-0.150	1.07E-05	-		-	
5	368394	*AHRR*	Body	cg12806681	-0.275	3.16E-08	-0.365	4.36E-13	-	
5	368447	*AHRR*	Body	cg03991871	-0.289	1.02E-09	-0.477	1.63E-21	-	
5	368804	*AHRR*	Body	cg23916896	-0.300	2.18E-09	-0.505	2.93E-19	-	
5	368843	*AHRR*	Body	cg11902777	-0.256	4.06E-08	-0.320	4.04E-14	-	
5	373299	*AHRR*	Body	cg23576855	-0.586	2.62E-44	-0.900	1.08E-14	-	
5	373378	*AHRR*	Body	cg05575921	-0.617	2.71E-48	-1.445	3.05E-106	-	
5	377358	*AHRR*	Body	cg26703534	-0.382	1.17E-27	-0.323	9.82E-59	-	
5	392920	*AHRR*	Body	cg14817490	-0.294	1.92E-12	-0.419	4.22E-37	-	
5	393347	*AHRR*	Body	cg17287155	-0.196	1.36E-07	-0.321	9.67E-17	-	
5	393366	*AHRR*	Body	cg04551776	-0.225	4.68E-08	-0.213	4.30E-20	-	
5	395444	*AHRR*	Body	cg25648203	-0.312	1.00E-15	-0.350	5.14E-37	-	
5	399360	*AHRR*	Body	cg21161138	-0.397	2.08E-17	-0.463	8.51E-57	-	
5	400732	*AHRR*	Body	cg24090911	-0.186	1.82E-06	-0.279	1.70E-19	-	
5	429559	*AHRR*	Body	cg26850624	0.166	5.16E-06	-		-	
5	14147618	*TRIO*	Body	cg10179300	-0.218	2.38E-05	-0.192	5.82E-09	-	

5	32018601	*PDZD2*	Body	cg13039251	0.192	4.60E-06	-		-
5	52096811	*ITGA1*	Body	cg16619991[d]	-0.157	1.42E-05	-		-
5	75878300	*IQGAP2*	Body	cg08595501	-0.219	2.02E-06	-		-
5	138861241	*TMEM173*	Body	cg04232128[d]	-0.094	2.24E-05	-		-
5	146614298	*STK32A*	TSS1500	cg09088988[d]	0.177	5.57E-07	-		-
5	149756966	*TCOF1*	Body	cg24032269[d]	0.121	1.59E-05	-		-
5	150161299	*C5orf62*	Body	cg14580211	-0.177	2.33E-06	-0.273	2.33E-23	-
5	174178256	-	-	cg24996482[d]	0.189	2.06E-05	-		-
6	30720080	-	-	cg06126421	-0.359	8.65E-17	-0.761	2.00E-60	-
6	30720108	-	-	cg14753356	-0.196	2.48E-09	-0.274	3.55E-34	-
6	30720203	-	-	cg24859433	-0.240	1.85E-07	-0.329	4.43E-35	-
6	30720209	-	-	cg15342087	-0.241	3.31E-08	-0.273	2.09E-32	-
6	30881112	*VARS2*	TSS1500	cg08617970[d]	-0.215	5.62E-06	-		-
6	30921592	*DPCR1*	3'UTR	cg11485823[d]	0.169	9.12E-06	-		-
6	35696870	*FKBP5*	TSS1500	cg25114611[d]	-0.124	4.69E-06	-		-
6	36645812	*CDKN1A*	TSS1500	cg15474579	-0.147	5.60E-07	-0.162	7.78E-14	-
6	36650733	*CDKN1A*	5'UTR	cg01955533[d]	-0.106	8.25E-06	-		-
6	41121920	*TREML1*	Body	cg01564343[d]	0.184	7.89E-06	-		-
6	46702983	*PLA2G7*	1stExon	cg18630040[d]	0.196	6.68E-07	-		-
7	1102177	*C7orf50*	Body	cg15693483[d]	-0.161	3.33E-09	-		-
7	1102226	*C7orf50*	Body	cg06009448[d]	-0.105	2.97E-05	-		-
7	2847517	*GNA12*	Body	cg09658497	-0.234	4.38E-06	-0.432	1.78E-09	-
7	2847554	*GNA12*	Body	cg19717773	-0.228	3.69E-06	-0.384	1.14E-10	-
7	2847575	*GNA12*	Body	cg18446336	-0.162	1.73E-05	-0.260	6.45E-11	-
7	5457225	*TNRC18*	Body	cg09022230	-0.229	3.51E-08	-0.159	3.13E-10	-
7	26578098	*KIAA0087*	Body	cg02451831	-0.203	2.75E-05	-0.217	1.37E-27	-
7	29606349	*PRR15*	3'UTR	cg06868100[d]	-0.222	5.71E-06	-		-
7	38370874	-	-	cg01726890[d]	0.152	3.35E-05	-		-
7	45002287	*MYO1G*	3'UTR	cg19089201	0.263	2.08E-08	-		-
7	45002486	*MYO1G*	Body	cg22132788	0.396	5.14E-16	1.070	3.57E-18	-
7	45002736	*MYO1G*	Body	cg04180046	0.224	1.56E-06	-		-
7	45002919	*MYO1G*	Body	cg12803068	0.358	1.10E-13	0.595	2.53E-15	-

7	45023329	*C7orf40*	Body	cg03440944	-0.175	1.31E-08	-0.115	1.32E-08	-
7	51384528	*COBL*	TSS200	cg09613161[d]	0.190	3.60E-05		-	-
7	51384609	*COBL*	TSS200	cg04016086[d]	0.204	1.10E-05		-	-
7	56147257	*SUMF2*	Body	cg19956914	0.233	1.51E-06		-	-
7	63642083	-	-	cg20164601[d]	-0.192	1.87E-05		-	-
7	98246001	*NPTX2*	TSS1500	cg13314145[d]	0.189	1.27E-05		-	-
7	100465833	*TRIP6*	Body	cg22851200	-0.186	2.19E-06	-0.181	1.40E-10	-
7	101556588	*CUX1*	Body	cg09762515	0.152	1.84E-05		-	-
7	116786606	*ST7*	Body	cg22619824[d]	-0.173	1.49E-05		-	-
7	130795028	*FLJ43663*	TSS1500	cg13990486[d]	-0.194	2.26E-05		-	-
7	145812842	*CNTNAP2*	TSS1500	cg21322436	-0.214	4.94E-06	-0.239	1.49E-24	-
7	145814306	*CNTNAP2*	Body	cg25949550	-0.168	8.19E-09	-0.242	3.09E-18	-
7	146904205	*CNTNAP2*	Body	cg11207515	0.204	1.64E-07	0.293	2.74E-16	-
7	147065665	*MIR548I4*	Body	cg15700587[d]	0.181	1.81E-07		-	-
7	156814480	-	-	cg09858188[d]	0.165	1.36E-05		-	-
7	158937969	*VIPR2*	TSS1500	cg23572908[d]	0.239	1.16E-06		-	-
8	11422167	-	-	cg05635807[d]	0.240	3.34E-06		-	-
8	17658543	*MTUS1*	TSS200	cg00778858[d]	0.193	1.05E-05		-	-
8	28258603	-	-	cg24540678	-0.203	8.80E-10	-0.168	4.63E-11	-
8	31496644	*NRG1*	TSS1500	cg18387156[d]	0.120	1.28E-05		-	-
8	41895100	*MYST3*	Body	cg14316231	-0.152	2.96E-05	-0.125	8.47E-12	-
8	65282185	-	-	cg25260137[d]	0.167	1.69E-05		-	-
8	93103361	*RUNX1T1*	5'UTR	cg03760919[d]	0.130	2.66E-05		-	-
8	105478855	*DPYS*	Body	cg16783744[d]	0.181	4.26E-06		-	-
8	126446923	*TRIB1*	Body	cg22644321[d]	-0.149	1.15E-05		-	-
8	128378218	-	-	cg25305703	-0.165	5.35E-07	-0.330	4.52E-20	-
8	142402728	-	-	cg12873476	-0.197	6.73E-06	-0.194	1.26E-08	-
8	144576604	*ZC3H3*	Body	cg26361535	-0.228	2.99E-07	-0.269	4.62E-16	-
8	145012644	*PLEC1*	Body	cg13389508[d]	-0.165	1.15E-05		-	-
8	145012748	*PLEC1*	Body	cg25325005	-0.146	1.00E-06		-	-
9	108005349	-	-	cg01692968	-0.250	3.01E-10	-0.293	1.36E-26	-
9	126105738	-	-	cg13418576	-0.195	9.78E-06		-	-

9	127054428	*NEK6*	TSS1500	cg14556677[d]	-0.111	7.99E-09		-	-
9	132803508	*FNBP1*	Body	cg06901890	-0.176	3.54E-05		-	-
10	8101566	*GATA3*	Body	cg17489908[d]	-0.154	4.80E-06		-	-
10	18429661	*CACNB2*	TSS200	cg20185017[d]	0.176	2.49E-05		-	-
10	21462441	*NEBL*	Body	cg17426273[d]	0.155	1.58E-05		-	-
10	43892075	*HNRNPF*	5'UTR	cg00326958	-0.169	2.46E-05		-	-
10	46970625	*SYT15*	TSS200	cg09373037[d]	0.203	8.44E-06		-	-
10	49892930	*WDFY4*	TSS1500	cg15164194[d]	-0.091	1.81E-06		-	-
10	73497514	*CDH23*	Body	cg10750182	-0.213	3.67E-11	-0.118	3.54E-19	-
10	99790170	*CRTAC1*	Body	cg04105282[d]	0.169	3.53E-05		-	-
10	112838983	*ADRA2A*	1stExon	cg05329352	-0.235	3.25E-07	-0.188	1.65E-08	-
10	116298339	*ABLIM1*	Body	cg07978738[d]	-0.145	3.62E-07		-	-
10	128994432	*DOCK1*	Body	cg03242819[d]	0.229	3.41E-07		-	-
10	128994644	*FAM196A*	TSS1500	cg03129384[d]	0.132	8.60E-06		-	-
11	2722391	*KCNQ1OT1*	TSS1500	cg07123182	-0.220	1.51E-05	-0.529	4.85E-15	-
11	2722401	*KCNQ1OT1*	TSS1500	cg16556677	-0.194	7.36E-06	-0.189	1.71E-13	-
11	2722407	*KCNQ1OT1*	TSS1500	cg26963277	-0.243	7.39E-07	-0.395	9.93E-18	-
11	5830030	-	-	cg12884422[d]	-0.213	1.91E-05		-	-
11	35161900	*CD44*	Body	cg23186333	-0.180	9.53E-06		-	-
11	58870075	-	-	cg16611234	-0.193	1.29E-05	-0.238	3.60E-13	-
11	60623782	*GPR44*	TSS1500	cg19254163	-0.176	1.92E-07	-0.142	2.31E-13	-
11	64379586	*NRXN2*	Body	cg27122888[d]	-0.195	4.48E-06		-	-
11	65196622	-	-	cg20889322	-0.204	4.77E-07		-	-
11	65201834	-	-	cg10416861[d]	0.216	8.67E-07		-	-
11	65550444	-	-	cg09419102[d]	-0.141	1.60E-06		-	-
11	68138269	*LRP5*	Body	cg21611682	-0.331	3.80E-14	-0.201	5.51E-33	-
11	68138505	*LRP5*	Body	cg10420527	-0.208	7.06E-08	-0.105	1.05E-14	-
11	68141470	*LRP5*	Body	cg09578155	-0.181	4.44E-07	-0.086	7.02E-10	-
11	68142198	*LRP5*	Body	cg14624207	-0.165	8.62E-07	-0.134	6.54E-14	-
11	71927938	*FOLR2*	1stExon	cg11295113[d]	0.124	1.18E-05		-	-
11	86510915	*PRSS23*	TSS1500	cg11660018	-0.256	1.36E-12	-0.261	6.89E-30	-
11	86510998	*PRSS23*	TSS1500	cg23771366	-0.185	1.42E-07	-0.230	1.09E-21	-

11	86512100	*PRSS23*	5'UTR	cg23351584	-0.179	1.26E-05	-		-	
11	116661388	*APOA5*	Body	cg09044186[d]	0.198	3.66E-05	-		-	
11	121322456	*SORL1*	TSS1500	cg10908953	-0.097	3.48E-05	-		-	
11	122709551	*CRTAM*	Body	cg22512531[d]	0.138	4.70E-07	-		-	
11	134095863	*VPS26B*	Body	cg09084200	-0.187	7.40E-09	-0.118	9.10E-11	-0.015	6.39E-06
12	4384888	*CCND2*	Body	cg07066369[d]	-0.206	1.27E-05	-		-	
12	4488800	*FGF23*	5'UTR	cg07178945[*]	0.223	7.62E-09	-		-	
12	7055657	*PTPN6*	TSS200	cg23193870[d]	-0.204	1.84E-06	-		-	
12	11898284	*ETV6*	Body	cg07986378	-0.185	1.08E-05	-0.307	2.57E-18	-	
12	25055967	*BCAT1*	Body	cg20399616[d]	0.228	1.02E-05	-		-	
12	54677008	*HNRNPA1*	Body	cg02583484	-0.147	5.76E-06	-0.164	3.13E-15	-	
12	56403172	-	-	cg21752525[d]	-0.192	1.83E-05	-		-	
12	66223718	*HMGA2*	Body	cg01598741[d]	-0.204	3.83E-06	-		-	
12	109025846	*SELPLG*	5'UTR	cg25165932[d]	0.197	1.30E-05	-		-	
12	113914222	-	-	cg02801786[d]	0.146	1.20E-05	-		-	
12	122518517	*MLXIP*	Body	cg03844971[d]	-0.178	1.62E-05	-		-	
12	124016861	*RILPL1*	Body	cg21618017[d]	-0.135	1.28E-05	-		-	
12	124726864	-	-	cg02869235[d]	0.135	2.57E-05	-		-	
12	124950720	*NCOR2*	Body	cg25922751[d]	-0.179	2.02E-05	-		-	
12	129337910	*GLT1D1*	TSS200	cg06419750[d]	0.180	2.98E-05	-		-	
13	29068987	*FLT1*	1stExon	cg22574825[d]	0.202	5.09E-06	-		-	
13	32889023	*BRCA2*	TSS1500	cg12836863	0.110	2.02E-05	-		-	
13	46964186	-	-	cg02985540	-0.112	1.88E-05	-		-	
13	48987165	*LPAR6*	5'UTR	cg03646329	-0.196	8.65E-07	-		-	
13	50702707	-	-	cg04214430[d]	-0.156	2.05E-05	-		-	
13	50702719	-	-	cg02003272	-0.173	3.87E-06	-0.263	6.32E-10	-	
13	50702795	-	-	cg13774342	-0.203	6.73E-07	-		-	
13	100632250	-	-	cg18656829[d]	0.207	1.45E-05	-		-	
13	111357885	*CARS2*	Body	cg20124610	-0.160	1.03E-06	-0.133	4.35E-12	-	
13	114172890	*TMCO3*	Body	cg00619505[d]	0.147	1.33E-05	-		-	
14	23450238	*JUB*	Body	cg06959340[d]	-0.214	3.06E-05	-		-	
14	24037169	*AP1G2*	5'UTR	cg02945646[d]	-0.095	3.43E-05	-		-	

14	25079357	*GZMH*	TSS1500	cg02150910[d]	0.136	2.71E-05		-	-
14	74214183	*C14orf43*	5'UTR	cg22851561	-0.146	3.90E-06	-0.160	5.81E-12	-
14	74223355	*C14orf43*	5'UTR	cg24996979	-0.172	2.20E-06	-0.129	1.03E-08	-
14	78051204	*SPTLC2*	Body	cg14544289[d]	-0.199	3.06E-06		-	-
14	89933549	*FOXN3*	5'UTR	cg13679772[d]	0.149	1.94E-06		-	-
14	91818668	*CCDC88C*	Body	cg01055824[d]	-0.169	8.47E-06		-	-
14	91881497	*CCDC88C*	Body	cg20303561	-0.150	2.31E-05	-0.164	3.14E-09	-
14	93552128	*ITPK1*	Body	cg05284742	-0.181	1.40E-07	-0.208	1.03E-29	-
14	104190678	*ZFYVE21*	Body	cg26242531	0.146	4.99E-07		-	-
14	104190829	*ZFYVE21*	Body	cg14977938	0.196	9.06E-06		-	-
14	104196038	*ZFYVE21*	Body	cg19838043	0.185	1.63E-05		-	-
14	105857250	*PACS2*	Body	cg12158535[d]	0.205	8.21E-06		-	-
14	106329158	-	-	cg01513913	-0.347	3.08E-14	-0.119	4.15E-09	-
14	106329206	-	-	cg13074055	-0.322	7.33E-11		-	-
14	106329607	-	-	cg23594345	-0.326	5.65E-12		-	-
14	106329652	-	-	cg01208318	-0.320	1.04E-12	-0.241	6.19E-09	-
14	106331803	-	-	cg14387626[d]	-0.142	4.34E-08		-	-
14	106354912	-	-	cg27113548[d]	-0.284	4.60E-09		-	-
14	106366503	-	-	cg00980649	-0.281	2.91E-10		-	-
15	38857474	*RASGRP1*	TSS1500	cg03603381	-0.186	2.58E-05		-	-
15	40076065	*FSIP1*	TSS1500	cg14428590[d]	-0.162	3.25E-05		-	-
15	55513621	*RAB27A*	Body	cg24687805[*]	0.158	5.69E-06		-	-
15	60296425	*FOXB1*	5'UTR	cg22777952[d]	0.171	2.01E-05		-	-
15	60959637	*RORA*	Body	cg15451980	0.129	3.48E-05		-	-
15	70387217	*TLE3*	Body	cg26971042	-0.189	1.29E-05		-	-
15	70387268	*TLE3*	Body	cg09747445[d]	-0.141	1.13E-05		-	-
15	74862662	*ARID3B*	Body	cg02384859[d]	0.142	4.45E-06		-	-
15	78384840	*SH2D7*	TSS200	cg18946533[d]	0.157	2.30E-05		-	-
15	84322591	*ADAMTSL3*	TSS1500	cg14096889[d]	0.184	2.15E-05		-	-
15	88798877	*NTRK3*	Body	cg20664238[d]	0.161	2.17E-05		-	-
15	90357202	*ANPEP*	5'UTR	cg23161492	-0.310	5.14E-12	-0.303	8.68E-19	-
15	96874050	*NR2F2*	TSS200	cg14858469[d]	0.199	2.00E-05		-	-

15	99194021	*IGF1R*	Body	cg07779120[d]	0.251	2.35E-07		-	-
16	3029641	*PKMYT1*	Body	cg04755561[d]	-0.146	1.52E-05		-	-
16	17562960	*XYLT1*	Body	cg06321596	-0.210	1.43E-07	-0.178	1.24E-09	-
16	28913517	*ATP2A1*	Body	cg02304156[d]	0.139	3.16E-05		-	-
16	30671749	-	-	cg07069636	-0.141	9.85E-07	-0.124	2.08E-13	-
16	83962037	-	-	cg04528720[d]	0.133	7.86E-06		-	-
16	84746995	*USP10*	Body	cg08126789[d]	0.179	3.04E-05		-	-
16	85721561	*GINS2*	Body	cg01383486[d]	-0.135	1.82E-05		-	-
16	89041793	*CBFA2T3*	Body	cg04887172[d]	-0.135	1.12E-05		-	-
16	89408248	*ANKRD11*	5'UTR	cg01107178[d]	0.118	1.83E-05		-	-
17	1104805	-	-	cg02352716[d]	0.128	3.64E-05		-	-
17	1553341	*RILP*	5'UTR	cg15380836[d]	-0.092	7.81E-06		-	-
17	4923126	*KIF1C*	Body	cg03877174[d]	0.154	7.12E-07		-	-
17	8804279	*PIK3R5*	Body	cg05460226	-0.156	9.32E-06	-0.203	5.37E-16	-
17	9921982	*GAS7*	Body	cg02018337[d]	-0.122	4.43E-07		-	-
17	27050723	*RPL23A*	Body	cg18150958[d]	-0.229	8.85E-07		-	-
17	27401793	*TIAF1*	5'UTR	cg18960216[d]	0.181	1.97E-07		-	-
17	38476024	*RARA*	5'UTR	cg19572487	-0.304	4.90E-14	-0.319	4.82E-29	-
17	45785206	*TBKBP1*	Body	cg23673974[d]	0.122	1.86E-05		-	-
17	46622522	*HOXB2*	TSS200	cg22807449[d]	-0.183	8.42E-06		-	-
17	53167407	*STXBP4*	Body	cg07465627	-0.172	1.33E-08	-0.106	9.09E-10	-
17	56082867	*SFRS1*	3'UTR	cg08591265[d]	-0.152	1.42E-06		-	-
17	58227267	*CA4*	TSS200	cg05248618[d]	0.211	1.31E-05		-	-
17	75371764	*SEPT9*	5'UTR	cg07827420[d]	-0.218	2.06E-05		-	-
17	75445905	*SEPT9*	Body	cg07324245	-0.108	1.07E-06		-	-
18	76739409	*SALL3*	TSS1500	cg05080154[d]	0.212	4.87E-08		-	-
19	1265879	-	-	cg00073090	-0.169	7.26E-08	-0.170	2.08E-21	-
19	2093896	*MOBKL2A*	5'UTR	cg15187398	-0.202	8.51E-07	-0.192	3.96E-11	-
19	2094327	*MOBKL2A*	5'UTR	cg07381806	-0.190	3.58E-05	-0.296	8.24E-12	-
19	2291020	*LINGO3*	Body	cg00378510[d]	-0.193	1.46E-05		-	-
19	2291373	*LINGO3*	Body	cg01294327	-0.236	4.65E-07		-	-
19	7986206	*SNAPC2*	Body	cg14074174	-0.158	1.94E-05	-0.093	5.91E-09	-

19	8677774	-	-	cg03172931[d]	0.184	4.56E-06			-	
19	12776725	*MORG1*	TSS1500	cg11621113	-0.125	1.53E-06	-0.135	5.08E-09	-	
19	13951168	-	-	cg05339037	-0.171	1.78E-05	-0.086	2.25E-08	-	
19	15528530	*AKAP8L*	Body	cg14588779[d]	-0.119	1.06E-05	-		-	
19	16284242	*CIB3*	1stExon	cg09686308[d]	0.173	1.16E-05	-		-	
19	17000585	*F2RL3*	Body	cg03636183	-0.471	2.78E-29	-0.660	5.37E-82	-0.096	6.21E-42
19	17003890	*CPAMD8*	3'UTR	cg15159987	-0.162	8.94E-06	-0.146	8.38E-15	-	
19	17534351	*FAM125A*	Body	cg22678402[d]	-0.093	1.80E-05	-		-	
19	18873222	*CRTC1*	Body	cg23973524	0.190	4.87E-07	0.195	9.15E-12	-	
19	18873268	*CRTC1*	Body	cg21473814	0.198	7.28E-06	-		-	
19	40919465	*PRX*	TSS200	cg01447828[d]	0.233	1.54E-07	-		-	
19	40919485	*PRX*	TSS1500	cg15393221	0.185	1.16E-07	-		-	
19	41768075	*HNRNPUL1*	TSS1500	cg13668129	-0.099	8.24E-06	-		-0.013	1.06E-07
19	51875451	*NKG7*	Body	cg10126923	0.114	1.77E-06	-		-	
19	51876788	*NKG7*	TSS1500	cg12916723[d]	0.179	2.01E-05	-		-	
19	51890586	*LIM2*	Body	cg01500140	0.106	2.72E-06	-		0.022	8.10E-07
19	53758055	*ZNF677*	5'UTR	cg03217253[d]	0.216	3.10E-07	-		-	
19	56701254	*ZSCAN5B*	Body	cg21733502[d]	-0.197	3.44E-05	-		-	
20	1310884	*SDCBP2*	TSS1500	cg05007126[d]	-0.137	3.17E-05	-		-	
20	6748730	*BMP2*	TSS200	cg13724496[d]	0.189	1.15E-05	-		-	
20	43438809	*RIMS4*	Body	cg15207742[d]	0.212	1.40E-06	-		-	
20	44069038	-	-	cg20344344[d]	-0.159	8.93E-06	-		-	
20	45985741	*ZMYND8*	TSS1500	cg12303084	-0.109	2.60E-05	-0.170	4.09E-12	-	
20	50312490	*ATP9A*	Body	cg07339236	-0.256	8.39E-10	-0.296	6.76E-13	-	
20	55204986	*TFAP2C*	Body	cg26736540[d]	0.169	3.19E-05	-		-	
20	61637856	*BHLHE23*	1stExon	cg06135139[d]	0.222	4.80E-06	-		-	
21	40180000	*ETS2*	5'UTR	cg15892280	-0.103	8.40E-06	-		-	
21	40182073	*ETS2*	Body	cg23110422	-0.255	9.38E-08	-0.258	5.45E-15	-	
21	47319304	*PCBP3*	Body	cg10204884[d]	-0.164	2.81E-05	-		-	
22	20792535	*SCARF2*	TSS1500	cg14785479[d]	0.123	1.10E-06	-		-	
22	31686097	*PIK3IP1*	Body	cg08548559	-0.154	2.62E-05	-		-	
22	37257404	*NCF4*	Body	cg02532700	-0.217	2.02E-06	-0.251	2.22E-11	-	

22	41633219	*CHADL*	Body	cg26364091	-0.197	1.86E-05	-	-
22	45403507	*PHF21B*	Body	cg06008724[d]	0.172	1.22E-05	-	-
22	48971959	*FAM19A5*	Body	cg04198308[d]	0.195	1.15E-05	-	-

Abbreviations: Chr, Chromosome; UTR, untranslated regions; Body, gene body; 1stExon, the first exon; TSS200, within 200 bps from transcription start site; TSS1500, within 1500 bps from transcription start site.

[a] Fixed effect meta-analysis, with sample size-weighted method to get *p* values, and an inverse-variance weighted method to get estimates of effect size. [b] Linear models with DNA methylation levels as the dependent variable, smoking status as the dependent variable, adjusting for micro-array, position of the sample on the micro-array and blood cell composition (Guida et al. 2015). [c] Linear mixed models with methylation beta values as the dependent variable, current smoker and pack-years as the primary independent variables, adjusting for age and sex (Sun et al. 2013). [d] CpGs that have not been reported to be significantly associated with smoking in previous genome-wide studies of methylation and smoking in Europeans (Guida et al. 2015; Shenker et al. 2013; Zeilinger et al. 2013) or in African Americans (Dogan et al 2014; Philibert et al. 2013; Sun et al. 2013).

[*] Bonferroni $p < 0.05$. [#] Bonferroni $p < 0.05$ or $FDR < 0.05$

Table S2. The effect size (s.e.) of smoking on the 318 CpGs with inverse normal transformed methylation values calculated in different groups.

CpG	Gene	Datasets of ACS patients				Datasets of healthy individuals			
		ACS in Wuhan (ACS-1, n = 101)		ACS in Guangdong (ACS-2, n = 97)		Individuals in Wuhan (COW-1&Wuhan residents of WHZH, n = 299)		Individuals in Guangdong (Zhuhai residents of WHZH, n = 99)	
		Effect (s.e.)	p	Effect (s.e.)	p	Effect (s.e.)	p	Effect (s.e.)	p
cg19708306	-	0.117 (0.061)	0.060	0.028 (0.089)	0.756	0.182 (0.042)	1.60E-05	0.092 (0.073)	0.210
cg05270224	-	0.254 (0.115)	0.030	0.139 (0.094)	0.142	0.193 (0.064)	0.003	0.219 (0.093)	0.021
cg05228408	*CLCN6*	-0.202 (0.087)	0.023	-0.211 (0.080)	0.010	-0.102 (0.049)	0.038	-0.189 (0.090)	0.038
cg05396397	*NPPA*	0.302 (0.121)	0.014	0.278 (0.104)	0.009	0.249 (0.070)	4.35E-04	0.214 (0.112)	0.059
cg04211179	*ZBTB17*	-0.120 (0.064)	0.065	-0.162 (0.059)	0.007	-0.138 (0.038)	3.69E-04	-0.169 (0.062)	0.008
cg07573717	*CAPZB*	-0.172 (0.044)	1.99E-04	-0.035 (0.039)	0.369	-0.138 (0.031)	1.03E-05	-0.039 (0.051)	0.449
cg19713429	*CAPZB*	-0.225 (0.100)	0.028	-0.254 (0.079)	0.002	-0.129 (0.048)	0.008	-0.196 (0.094)	0.040
cg26348226	*ECE1*	-0.143 (0.078)	0.069	-0.243 (0.079)	0.003	-0.133 (0.043)	0.002	-0.100 (0.063)	0.118
cg17094249	-	-0.136 (0.122)	0.268	-0.254 (0.114)	0.029	-0.295 (0.075)	1.00E-04	-0.123 (0.119)	0.302
cg02818189	-	-0.048 (0.078)	0.541	-0.100 (0.068)	0.146	-0.143 (0.046)	0.002	-0.219 (0.068)	0.002
cg10951873	*RUNX3*	-0.191 (0.095)	0.048	-0.153 (0.086)	0.081	-0.140 (0.055)	0.012	-0.345 (0.091)	3.02E-04
cg27537125	-	-0.180 (0.057)	0.002	-0.071 (0.052)	0.173	-0.120 (0.033)	2.70E-04	-0.252 (0.057)	2.74E-05
cg20460771	*PTAFR*	0.202 (0.076)	0.009	0.096 (0.085)	0.266	0.114 (0.038)	0.003	0.161 (0.068)	0.020
cg25310233	-	-0.111 (0.074)	0.138	-0.129 (0.071)	0.072	-0.132 (0.039)	7.51E-04	-0.105 (0.076)	0.172
cg14663208	*HIVEP3*	0.197 (0.095)	0.042	0.031 (0.117)	0.795	0.200 (0.046)	1.78E-05	0.241 (0.076)	0.002
cg16145216	*HIVEP3*	0.260 (0.109)	0.020	0.169 (0.096)	0.083	0.136 (0.065)	0.037	0.308 (0.097)	0.002
cg24741609	*GLIS1*	-0.074 (0.097)	0.453	-0.195 (0.080)	0.017	-0.181 (0.052)	5.84E-04	-0.260 (0.093)	0.006
cg04158878	-	0.280 (0.109)	0.012	0.175 (0.092)	0.060	0.141 (0.047)	0.003	0.059 (0.087)	0.497
cg25189904	*GNG12*	-0.299 (0.086)	7.59E-04	-0.134 (0.105)	0.206	-0.255 (0.064)	9.25E-05	-0.518 (0.096)	6.90E-07
cg20146909	*LRRC8D*	-0.313 (0.096)	0.002	-0.105 (0.098)	0.287	-0.150 (0.057)	0.009	-0.238 (0.098)	0.017

cg12876356	*GFI1*	-0.202 (0.101)	0.050	-0.177 (0.102)	0.088	-0.188 (0.058)	0.001	-0.150 (0.092)	0.108
cg09935388	*GFI1*	-0.267 (0.113)	0.020	-0.340 (0.100)	0.001	-0.268 (0.064)	3.97E-05	-0.303 (0.095)	0.002
cg08129092	*INTS3*	0.088 (0.066)	0.184	0.011 (0.069)	0.870	0.157 (0.041)	1.69E-04	0.162 (0.069)	0.022
cg23924887	*ATP8B2*	-0.149 (0.071)	0.040	-0.153 (0.057)	0.009	-0.148 (0.039)	1.69E-04	0.050 (0.059)	0.398
cg06811467	*ATP8B2*	-0.170 (0.050)	9.80E-04	-0.105 (0.058)	0.076	-0.139 (0.028)	1.62E-06	-0.032 (0.059)	0.595
cg09257526	*IL6R*	-0.129 (0.053)	0.017	-0.049 (0.046)	0.287	-0.119 (0.030)	7.39E-05	-0.151 (0.049)	0.003
cg12593793	-	-0.231 (0.058)	1.60E-04	-0.046 (0.059)	0.437	-0.070 (0.036)	0.051	-0.155 (0.056)	0.007
cg01416295	*MRPL24*	-0.118 (0.096)	0.226	-0.151 (0.095)	0.114	-0.111 (0.053)	0.038	-0.410 (0.082)	3.03E-06
cg09471611	-	0.239 (0.086)	0.007	0.094 (0.109)	0.393	0.157 (0.040)	1.29E-04	0.024 (0.085)	0.782
cg08709672	*AVPR1B*	-0.145 (0.102)	0.157	-0.395 (0.094)	7.34E-05	-0.140 (0.067)	0.036	-0.399 (0.115)	8.68E-04
cg11229399	-	0.262 (0.108)	0.018	0.099 (0.101)	0.331	0.227 (0.059)	1.70E-04	0.136 (0.101)	0.184
cg23079012	-	-0.399 (0.113)	7.05E-04	-0.276 (0.099)	0.007	-0.239 (0.057)	3.87E-05	-0.299 (0.091)	0.001
cg08035323	-	0.389 (0.108)	5.48E-04	0.316 (0.107)	0.004	0.177 (0.053)	0.001	0.356 (0.084)	5.66E-05
cg02560388	-	-0.185 (0.092)	0.047	-0.085 (0.089)	0.345	-0.226 (0.053)	2.96E-05	-0.142 (0.089)	0.116
cg13711966	-	0.322 (0.091)	6.70E-04	0.132 (0.086)	0.127	0.130 (0.054)	0.017	0.163 (0.083)	0.051
cg05010058	*CEP68*	-0.045 (0.101)	0.659	-0.100 (0.098)	0.308	-0.264 (0.063)	4.31E-05	-0.194 (0.097)	0.049
cg15746583	*CD8B*	0.079 (0.058)	0.180	0.122 (0.048)	0.012	0.100 (0.030)	0.001	0.055 (0.051)	0.277
cg09570614	-	0.252 (0.074)	0.001	0.032 (0.081)	0.691	0.101 (0.047)	0.033	0.224 (0.080)	0.006
cg01765406	-	-0.100 (0.072)	0.166	0.048 (0.068)	0.480	-0.143 (0.041)	5.19E-04	-0.207 (0.057)	5.00E-04
cg20949306	*RAB3GAP1*	0.414 (0.109)	2.85E-04	0.151 (0.112)	0.183	0.148 (0.060)	0.015	0.102 (0.095)	0.284
cg14667406	*LCT*	-0.090 (0.122)	0.460	-0.319 (0.112)	0.005	-0.210 (0.066)	0.002	-0.215 (0.111)	0.057
cg22674699	*HOXD9*	0.284 (0.103)	0.007	0.154 (0.117)	0.191	0.213 (0.059)	3.70E-04	0.204 (0.102)	0.049
cg16382047	*GPR55*	-0.157 (0.079)	0.049	-0.063 (0.059)	0.285	-0.132 (0.037)	4.25E-04	-0.170 (0.062)	0.007
cg19827923	*GPR55*	-0.189 (0.087)	0.033	-0.216 (0.066)	0.002	-0.101 (0.048)	0.036	-0.207 (0.068)	0.003
cg03329539	-	-0.314 (0.075)	7.00E-05	-0.402 (0.091)	3.11E-05	-0.272 (0.050)	1.01E-07	-0.350 (0.091)	2.53E-04
cg06644428	-	-0.309 (0.096)	0.002	-0.332 (0.107)	0.003	-0.179 (0.061)	0.004	-0.309 (0.082)	3.09E-04
cg05951221	-	-0.502 (0.081)	2.29E-08	-0.480 (0.088)	5.15E-07	-0.493 (0.058)	1.44E-15	-0.479 (0.092)	1.25E-06
cg21566642	-	-0.683 (0.100)	1.21E-09	-0.658 (0.093)	4.84E-10	-0.516 (0.061)	1.47E-15	-0.607 (0.100)	4.02E-08

cg01940273	-	-0.608 (0.102)	5.95E-08	-0.718 (0.096)	6.74E-11	-0.436 (0.06)	5.77E-12	-0.555 (0.092)	3.97E-08
cg13193840	-	-0.309 (0.116)	0.009	-0.344 (0.120)	0.005	-0.121 (0.073)	0.098	-0.322 (0.115)	0.006
cg13481776	*ALPI*	0.231 (0.079)	0.004	0.082 (0.084)	0.331	0.099 (0.042)	0.018	0.169 (0.067)	0.014
cg12756150	-	0.140 (0.114)	0.222	0.128 (0.106)	0.233	0.219 (0.062)	4.77E-04	0.275 (0.106)	0.011
cg13279811	-	-0.282 (0.096)	0.004	-0.089 (0.100)	0.380	-0.168 (0.060)	0.006	-0.167 (0.101)	0.102
cg00501876	*CSRNP1*	-0.243 (0.105)	0.023	-0.214 (0.082)	0.010	-0.302 (0.053)	3.98E-08	-0.232 (0.096)	0.018
cg18642234	*GPX1*	-0.211 (0.113)	0.066	-0.235 (0.095)	0.016	-0.183 (0.060)	0.002	-0.183 (0.087)	0.038
cg19784816	*ITIH1*	0.159 (0.073)	0.031	0.168 (0.067)	0.014	0.079 (0.046)	0.085	0.233 (0.088)	0.010
cg15417641	*CACNA1D*	0.196 (0.120)	0.106	0.249 (0.118)	0.038	0.215 (0.067)	0.001	0.233 (0.105)	0.029
cg00336149	*CACNA1D*	0.230 (0.124)	0.069	0.126 (0.124)	0.312	0.181 (0.070)	0.011	0.319 (0.109)	0.004
cg21188533	*CACNA1D*	0.248 (0.126)	0.053	0.185 (0.118)	0.121	0.227 (0.074)	0.002	0.242 (0.110)	0.030
cg09301294	*EPHA6*	0.113 (0.094)	0.234	0.167 (0.096)	0.087	0.139 (0.051)	0.006	0.242 (0.083)	0.005
cg19859270	*GPR15*	-0.439 (0.117)	3.32E-04	-0.214 (0.110)	0.056	-0.269 (0.069)	1.09E-04	-0.224 (0.114)	0.053
cg15554421	*C3orf26*	-0.143 (0.084)	0.092	-0.169 (0.066)	0.013	-0.134 (0.046)	0.004	-0.219 (0.070)	0.002
cg05655806	*CD96*	-0.233 (0.112)	0.041	-0.140 (0.088)	0.113	-0.184 (0.063)	0.004	-0.269 (0.099)	0.008
cg04039397	*CD96*	-0.026 (0.116)	0.823	-0.128 (0.106)	0.231	-0.231 (0.069)	9.73E-04	-0.383 (0.111)	8.78E-04
cg18165852	*CHST13*	0.080 (0.106)	0.452	0.085 (0.104)	0.415	0.230 (0.060)	1.64E-04	0.263 (0.105)	0.014
cg26958735	-	0.106 (0.098)	0.284	0.089 (0.097)	0.365	0.190 (0.057)	0.001	0.294 (0.098)	0.004
cg25853622	*LPP*	-0.112 (0.038)	0.004	-0.042 (0.034)	0.219	-0.079 (0.023)	5.75E-04	-0.010 (0.030)	0.733
cg13185177	*GP5*	0.278 (0.082)	0.001	0.159 (0.076)	0.040	0.090 (0.046)	0.055	0.114 (0.067)	0.092
cg23867146	-	0.333 (0.110)	0.003	0.259 (0.094)	0.007	0.137 (0.067)	0.041	0.140 (0.093)	0.136
cg10351287	*STK32B*	0.107 (0.105)	0.314	0.102 (0.104)	0.328	0.261 (0.065)	8.44E-05	0.194 (0.097)	0.049
cg19719391	-	0.183 (0.102)	0.076	0.079 (0.085)	0.359	0.198 (0.050)	9.21E-05	0.071 (0.080)	0.375
cg26542660	*CEP135*	-0.199 (0.062)	0.002	-0.108 (0.062)	0.083	-0.161 (0.037)	2.34E-05	-0.110 (0.065)	0.094
cg09156233	*BMPR1B*	0.151 (0.099)	0.133	0.231 (0.104)	0.029	0.239 (0.062)	1.34E-04	0.112 (0.099)	0.264
cg11554391	*AHRR*	-0.058 (0.110)	0.596	-0.173 (0.098)	0.080	-0.272 (0.060)	9.93E-06	-0.244 (0.096)	0.013
cg17924476	*AHRR*	0.164 (0.103)	0.114	0.144 (0.086)	0.096	0.149 (0.045)	0.001	0.297 (0.089)	0.001
cg09338136	*AHRR*	-0.207 (0.076)	0.008	-0.037 (0.087)	0.672	-0.158 (0.048)	0.001	-0.157 (0.075)	0.039

cg12806681	*AHRR*	-0.477 (0.117)	9.68E-05	-0.284 (0.120)	0.020	-0.243 (0.069)	4.50E-04	-0.150 (0.120)	0.214
cg03991871	*AHRR*	-0.433 (0.113)	2.50E-04	-0.268 (0.111)	0.018	-0.281 (0.068)	4.93E-05	-0.202 (0.106)	0.060
cg23916896	*AHRR*	-0.467 (0.102)	1.57E-05	-0.279 (0.117)	0.019	-0.238 (0.071)	8.77E-04	-0.269 (0.119)	0.026
cg11902777	*AHRR*	-0.383 (0.099)	2.23E-04	-0.119 (0.104)	0.255	-0.223 (0.068)	0.001	-0.338 (0.110)	0.003
cg23576855	*AHRR*	-0.695 (0.103)	1.74E-09	-0.517 (0.100)	1.56E-06	-0.575 (0.053)	8.02E-23	-0.591 (0.082)	2.41E-10
cg05575921	*AHRR*	-0.648 (0.089)	1.52E-10	-0.481 (0.094)	2.12E-06	-0.582 (0.055)	2.89E-22	-0.768 (0.081)	6.06E-15
cg26703534	*AHRR*	-0.497 (0.093)	6.92E-07	-0.321 (0.079)	1.17E-04	-0.372 (0.046)	3.45E-14	-0.389 (0.076)	2.11E-06
cg14817490	*AHRR*	-0.351 (0.087)	1.19E-04	-0.190 (0.091)	0.041	-0.286 (0.062)	5.32E-06	-0.367 (0.102)	5.57E-04
cg17287155	*AHRR*	-0.106 (0.094)	0.259	-0.286 (0.072)	1.42E-04	-0.127 (0.051)	0.013	-0.353 (0.090)	1.91E-04
cg04551776	*AHRR*	-0.215 (0.108)	0.048	-0.130 (0.088)	0.145	-0.221 (0.059)	2.18E-04	-0.353 (0.096)	3.97E-04
cg25648203	*AHRR*	-0.387 (0.101)	2.48E-04	-0.195 (0.089)	0.032	-0.346 (0.054)	6.23E-10	-0.279 (0.087)	0.002
cg21161138	*AHRR*	-0.481 (0.112)	4.82E-05	-0.287 (0.102)	0.006	-0.370 (0.066)	4.64E-08	-0.513 (0.107)	6.39E-06
cg24090911	*AHRR*	-0.127 (0.087)	0.146	-0.135 (0.092)	0.147	-0.222 (0.057)	1.34E-04	-0.212 (0.096)	0.030
cg26850624	*AHRR*	0.293 (0.081)	4.92E-04	0.078 (0.089)	0.385	0.180 (0.048)	2.28E-04	0.038 (0.094)	0.690
cg10179300	*TRIO*	-0.055 (0.126)	0.664	-0.141 (0.119)	0.237	-0.280 (0.076)	3.01E-04	-0.286 (0.113)	0.013
cg13039251	*PDZD2*	0.145 (0.103)	0.162	0.231 (0.095)	0.017	0.138 (0.059)	0.020	0.340 (0.096)	6.67E-04
cg16619991	*ITGA1*	-0.234 (0.091)	0.012	-0.234 (0.075)	0.003	-0.193 (0.050)	1.59E-04	0.128 (0.088)	0.150
cg08595501	*IQGAP2*	-0.124 (0.105)	0.242	-0.003 (0.114)	0.979	-0.320 (0.066)	1.95E-06	-0.238 (0.107)	0.029
cg04232128	*TMEM173*	-0.190 (0.045)	6.49E-05	-0.007 (0.047)	0.886	-0.111 (0.031)	4.28E-04	-0.006 (0.059)	0.917
cg09088988	*STK32A*	0.118 (0.076)	0.123	0.129 (0.081)	0.113	0.267 (0.054)	1.35E-06	0.068 (0.090)	0.449
cg24032269	*TCOF1*	0.141 (0.068)	0.042	0.048 (0.067)	0.480	0.123 (0.040)	0.002	0.160 (0.061)	0.011
cg14580211	*C5orf62*	-0.048 (0.093)	0.609	-0.267 (0.078)	9.61E-04	-0.158 (0.053)	0.003	-0.225 (0.083)	0.009
cg24996482	-	0.061 (0.109)	0.579	0.227 (0.103)	0.030	0.225 (0.062)	3.24E-04	0.161 (0.107)	0.134
cg06126421	-	-0.369 (0.096)	2.35E-04	-0.335 (0.103)	0.002	-0.325 (0.060)	1.66E-07	-0.452 (0.094)	7.11E-06
cg14753356	-	-0.222 (0.079)	0.006	-0.140 (0.074)	0.061	-0.194 (0.049)	1.13E-04	-0.227 (0.067)	0.001
cg24859433	-	-0.193 (0.110)	0.084	-0.301 (0.112)	0.009	-0.229 (0.064)	4.34E-04	-0.257 (0.108)	0.020
cg15342087	-	-0.202 (0.111)	0.072	-0.250 (0.099)	0.014	-0.199 (0.060)	0.001	-0.403 (0.107)	3.16E-04
cg08617970	*VARS2*	-0.321 (0.108)	0.004	-0.195 (0.114)	0.091	-0.205 (0.068)	0.003	-0.148 (0.110)	0.181

cg11485823	*DPCR1*	0.210 (0.092)	0.025	0.147 (0.093)	0.117	0.136 (0.052)	0.010	0.259 (0.097)	0.009
cg25114611	*FKBP5*	-0.159 (0.073)	0.033	-0.079 (0.062)	0.206	-0.141 (0.037)	1.98E-04	-0.094 (0.068)	0.173
cg15474579	*CDKN1A*	-0.224 (0.062)	4.99E-04	-0.092 (0.070)	0.193	-0.117 (0.041)	0.005	-0.197 (0.075)	0.010
cg01955533	*CDKN1A*	-0.189 (0.056)	0.001	0.004 (0.058)	0.945	-0.098 (0.034)	0.005	-0.137 (0.048)	0.005
cg01564343	*TREML1*	0.221 (0.099)	0.028	0.347 (0.101)	9.28E-04	0.109 (0.057)	0.057	0.212 (0.097)	0.031
cg18630040	*PLA2G7*	0.094 (0.107)	0.381	0.165 (0.092)	0.075	0.210 (0.055)	1.51E-04	0.246 (0.080)	0.003
cg15693483	*C7orf50*	-0.200 (0.075)	0.010	-0.156 (0.057)	0.008	-0.157 (0.039)	8.82E-05	-0.150 (0.060)	0.015
cg06009448	*C7orf50*	-0.191 (0.066)	0.005	-0.185 (0.057)	0.002	-0.048 (0.035)	0.175	-0.111 (0.056)	0.051
cg09658497	*GNA12*	-0.271 (0.125)	0.033	-0.313 (0.108)	0.005	-0.213 (0.074)	0.004	-0.160 (0.117)	0.177
cg19717773	*GNA12*	-0.231 (0.125)	0.067	-0.302 (0.106)	0.005	-0.218 (0.071)	0.003	-0.167 (0.110)	0.133
cg18446336	*GNA12*	-0.238 (0.089)	0.009	-0.153 (0.076)	0.048	-0.166 (0.058)	0.004	-0.091 (0.089)	0.308
cg09022230	*TNRC18*	-0.320 (0.099)	0.002	-0.279 (0.098)	0.006	-0.169 (0.058)	0.004	-0.261 (0.099)	0.010
cg02451831	*KIAA0087*	-0.379 (0.104)	4.73E-04	-0.100 (0.116)	0.395	-0.190 (0.068)	0.006	-0.129 (0.113)	0.255
cg06868100	*PRR15*	-0.071 (0.114)	0.537	-0.368 (0.117)	0.002	-0.211 (0.070)	0.003	-0.259 (0.111)	0.022
cg01726890	-	0.165 (0.088)	0.064	0.210 (0.093)	0.026	0.129 (0.051)	0.013	0.155 (0.086)	0.075
cg19089201	*MYO1G*	0.075 (0.111)	0.504	0.249 (0.109)	0.025	0.371 (0.063)	1.11E-08	0.110 (0.117)	0.352
cg22132788	*MYO1G*	0.276 (0.122)	0.026	0.421 (0.113)	3.71E-04	0.447 (0.065)	5.58E-11	0.321 (0.115)	0.006
cg04180046	*MYO1G*	0.141 (0.113)	0.214	0.245 (0.117)	0.039	0.261 (0.065)	7.91E-05	0.181 (0.110)	0.104
cg12803068	*MYO1G*	0.285 (0.120)	0.020	0.441 (0.111)	1.41E-04	0.378 (0.065)	2.16E-08	0.275 (0.113)	0.017
cg03440944	*C7orf40*	-0.204 (0.061)	0.001	-0.017 (0.076)	0.825	-0.189 (0.044)	2.43E-05	-0.252 (0.078)	0.002
cg09613161	*COBL*	0.244 (0.116)	0.038	0.052 (0.126)	0.680	0.231 (0.064)	3.56E-04	0.140 (0.097)	0.154
cg04016086	*COBL*	0.194 (0.115)	0.096	0.087 (0.114)	0.447	0.303 (0.064)	3.29E-06	0.029 (0.109)	0.787
cg19956914	*SUMF2*	0.203 (0.125)	0.109	0.249 (0.100)	0.014	0.181 (0.070)	0.010	0.343 (0.101)	0.001
cg20164601	-	-0.133 (0.114)	0.246	-0.102 (0.097)	0.298	-0.267 (0.066)	6.18E-05	-0.150 (0.111)	0.182
cg13314145	*NPTX2*	0.262 (0.096)	0.008	0.271 (0.109)	0.015	0.150 (0.062)	0.017	0.145 (0.099)	0.147
cg22851200	*TRIP6*	-0.201 (0.092)	0.032	-0.201 (0.094)	0.035	-0.143 (0.056)	0.011	-0.273 (0.091)	0.003
cg09762515	*CUX1*	0.247 (0.083)	0.004	0.193 (0.088)	0.032	0.087 (0.049)	0.077	0.207 (0.084)	0.016
cg22619824	*ST7*	-0.110 (0.090)	0.226	-0.139 (0.098)	0.159	-0.210 (0.060)	5.92E-04	-0.181 (0.087)	0.042

cg13990486	*FLJ43663*	-0.147 (0.109)	0.181	-0.161 (0.099)	0.110	-0.243 (0.069)	5.20E-04	-0.157 (0.109)	0.153
cg21322436	*CNTNAP2*	-0.198 (0.121)	0.106	-0.286 (0.104)	0.007	-0.187 (0.067)	0.006	-0.217 (0.099)	0.032
cg25949550	*CNTNAP2*	-0.153 (0.064)	0.019	-0.199 (0.070)	0.005	-0.153 (0.044)	6.11E-04	-0.187 (0.059)	0.002
cg11207515	*CNTNAP2*	0.206 (0.093)	0.030	0.185 (0.087)	0.036	0.203 (0.056)	3.64E-04	0.225 (0.092)	0.017
cg15700587	*MIR548I4*	0.102 (0.091)	0.268	0.119 (0.086)	0.171	0.189 (0.046)	4.66E-05	0.275 (0.082)	0.001
cg09858188	-	0.091 (0.088)	0.304	0.006 (0.093)	0.947	0.219 (0.055)	8.31E-05	0.235 (0.084)	0.006
cg23572908	*VIPR2*	0.267 (0.122)	0.032	0.106 (0.130)	0.418	0.254 (0.066)	1.46E-04	0.274 (0.114)	0.019
cg05635807	-	0.173 (0.123)	0.162	0.176 (0.124)	0.158	0.194 (0.071)	0.007	0.451 (0.110)	9.69E-05
cg00778858	*MTUS1*	0.245 (0.114)	0.035	0.120 (0.106)	0.260	0.226 (0.064)	4.51E-04	0.142 (0.096)	0.143
cg24540678	-	-0.293 (0.068)	4.82E-05	-0.049 (0.073)	0.506	-0.211 (0.048)	1.59E-05	-0.250 (0.088)	0.006
cg18387156	*NRG1*	0.032 (0.071)	0.653	0.195 (0.054)	5.64E-04	0.099 (0.039)	0.012	0.141 (0.056)	0.014
cg14316231	*MYST3*	-0.136 (0.091)	0.142	-0.133 (0.081)	0.104	-0.194 (0.051)	1.78E-04	-0.059 (0.091)	0.522
cg25260137	-	0.158 (0.096)	0.104	0.246 (0.108)	0.025	0.135 (0.054)	0.014	0.198 (0.081)	0.016
cg03760919	*RUNX1T1*	0.158 (0.071)	0.028	0.147 (0.075)	0.055	0.079 (0.043)	0.071	0.223 (0.071)	0.002
cg16783744	*DPYS*	0.140 (0.095)	0.143	0.145 (0.102)	0.161	0.222 (0.055)	7.63E-05	0.138 (0.089)	0.124
cg22644321	*TRIB1*	-0.365 (0.082)	2.40E-05	0.010 (0.096)	0.917	-0.131 (0.044)	0.003	-0.114 (0.078)	0.146
cg25305703	-	-0.229 (0.060)	2.71E-04	-0.068 (0.069)	0.328	-0.175 (0.051)	7.23E-04	-0.157 (0.087)	0.075
cg12873476	-	-0.255 (0.104)	0.016	-0.168 (0.107)	0.122	-0.139 (0.061)	0.024	-0.307 (0.093)	0.002
cg26361535	*ZC3H3*	-0.281 (0.094)	0.004	-0.412 (0.099)	7.60E-05	-0.131 (0.063)	0.039	-0.222 (0.103)	0.034
cg13389508	*PLEC1*	-0.188 (0.083)	0.026	-0.200 (0.084)	0.020	-0.116 (0.053)	0.030	-0.261 (0.105)	0.015
cg25325005	*PLEC1*	-0.175 (0.073)	0.018	-0.040 (0.061)	0.515	-0.188 (0.048)	1.20E-04	-0.181 (0.078)	0.022
cg01692968	-	-0.188 (0.097)	0.056	-0.234 (0.096)	0.017	-0.251 (0.055)	7.78E-06	-0.315 (0.089)	6.88E-04
cg13418576	-	-0.119 (0.100)	0.238	-0.185 (0.111)	0.099	-0.227 (0.064)	4.74E-04	-0.203 (0.103)	0.051
cg14556677	*NEK6*	-0.090 (0.052)	0.088	-0.099 (0.040)	0.015	-0.123 (0.027)	6.83E-06	-0.105 (0.047)	0.028
cg06901890	*FNBP1*	-0.218 (0.089)	0.016	-0.181 (0.085)	0.036	-0.112 (0.064)	0.082	-0.275 (0.102)	0.009
cg17489908	*GATA3*	-0.168 (0.095)	0.081	-0.162 (0.083)	0.055	-0.159 (0.047)	9.52E-04	-0.131 (0.071)	0.070
cg20185017	*CACNB2*	0.069 (0.097)	0.476	0.305 (0.110)	0.007	0.131 (0.060)	0.030	0.252 (0.077)	0.002
cg17426273	*NEBL*	0.078 (0.080)	0.333	0.166 (0.097)	0.091	0.237 (0.050)	2.94E-06	-0.016 (0.088)	0.857

cg00326958	*HNRNPF*	-0.048 (0.092)	0.603	-0.238 (0.100)	0.019	-0.221 (0.060)	3.00E-04	-0.114 (0.092)	0.220
cg09373037	*SYT15*	0.157 (0.097)	0.111	0.015 (0.116)	0.900	0.258 (0.067)	1.49E-04	0.268 (0.101)	0.009
cg15164194	*WDFY4*	-0.145 (0.047)	0.003	0.025 (0.042)	0.556	-0.124 (0.029)	2.01E-05	-0.096 (0.050)	0.060
cg10750182	*CDH23*	-0.138 (0.085)	0.108	-0.260 (0.084)	0.003	-0.211 (0.044)	2.82E-06	-0.234 (0.064)	4.48E-04
cg04105282	*CRTAC1*	0.103 (0.098)	0.296	0.288 (0.093)	0.003	0.139 (0.057)	0.015	0.194 (0.100)	0.057
cg05329352	*ADRA2A*	-0.206 (0.113)	0.073	-0.177 (0.105)	0.096	-0.247 (0.067)	3.07E-04	-0.285 (0.099)	0.005
cg07978738	*ABLIM1*	-0.215 (0.070)	0.003	-0.077 (0.070)	0.275	-0.174 (0.041)	2.86E-05	-0.073 (0.064)	0.258
cg03242819	*DOCK1*	0.200 (0.107)	0.064	0.252 (0.101)	0.014	0.217 (0.065)	0.001	0.258 (0.101)	0.012
cg03129384	*FAM196A*	0.081 (0.068)	0.236	0.176 (0.081)	0.033	0.163 (0.043)	2.19E-04	0.079 (0.066)	0.235
cg07123182	*KCNQ1OT1*	-0.186 (0.114)	0.106	-0.302 (0.115)	0.010	-0.139 (0.074)	0.060	-0.348 (0.106)	0.002
cg16556677	*KCNQ1OT1*	-0.254 (0.097)	0.010	-0.325 (0.097)	0.001	-0.092 (0.061)	0.133	-0.253 (0.095)	0.009
cg26963277	*KCNQ1OT1*	-0.221 (0.115)	0.057	-0.242 (0.119)	0.045	-0.230 (0.070)	0.001	-0.300 (0.114)	0.011
cg12884422	-	-0.213 (0.127)	0.097	-0.095 (0.116)	0.416	-0.197 (0.070)	0.005	-0.358 (0.109)	0.001
cg23186333	*CD44*	-0.155 (0.102)	0.130	-0.042 (0.091)	0.645	-0.274 (0.058)	3.41E-06	-0.083 (0.103)	0.420
cg16611234	-	-0.100 (0.099)	0.319	-0.210 (0.112)	0.065	-0.208 (0.065)	0.001	-0.236 (0.099)	0.020
cg19254163	*GPR44*	-0.191 (0.081)	0.020	-0.187 (0.093)	0.049	-0.175 (0.045)	1.15E-04	-0.155 (0.086)	0.076
cg27122888	*NRXN2*	-0.206 (0.101)	0.045	-0.054 (0.106)	0.614	-0.241 (0.060)	7.13E-05	-0.179 (0.097)	0.068
cg20889322	-	-0.247 (0.096)	0.012	-0.139 (0.087)	0.116	-0.181 (0.060)	0.003	-0.283 (0.089)	0.002
cg10416861	-	0.116 (0.101)	0.252	0.328 (0.103)	0.002	0.219 (0.064)	7.42E-04	0.204 (0.101)	0.047
cg09419102	-	-0.110 (0.068)	0.109	-0.124 (0.065)	0.060	-0.151 (0.043)	4.95E-04	-0.175 (0.077)	0.025
cg21611682	*LRP5*	-0.384 (0.096)	1.38E-04	-0.228 (0.099)	0.024	-0.307 (0.064)	2.77E-06	-0.418 (0.091)	1.42E-05
cg10420527	*LRP5*	-0.241 (0.102)	0.021	-0.145 (0.074)	0.054	-0.182 (0.059)	0.002	-0.322 (0.084)	2.40E-04
cg09578155	*LRP5*	-0.272 (0.081)	0.001	-0.002 (0.078)	0.979	-0.205 (0.055)	2.27E-04	-0.239 (0.087)	0.007
cg14624207	*LRP5*	-0.227 (0.078)	0.005	-0.124 (0.070)	0.079	-0.148 (0.051)	0.004	-0.194 (0.077)	0.013
cg11295113	*FOLR2*	0.157 (0.068)	0.022	0.059 (0.056)	0.294	0.124 (0.045)	0.006	0.200 (0.074)	0.008
cg11660018	*PRSS23*	-0.213 (0.079)	0.008	-0.336 (0.087)	2.37E-04	-0.248 (0.052)	3.86E-06	-0.255 (0.081)	0.002
cg23771366	*PRSS23*	-0.157 (0.068)	0.024	-0.140 (0.092)	0.131	-0.230 (0.052)	1.15E-05	-0.138 (0.090)	0.130
cg23351584	*PRSS23*	-0.140 (0.092)	0.132	-0.161 (0.086)	0.065	-0.163 (0.062)	0.010	-0.286 (0.097)	0.004

cg09044186	*APOA5*	0.263 (0.117)	0.028	0.042 (0.117)	0.719	0.264 (0.066)	7.65E-05	0.079 (0.117)	0.500
cg10908953	*SORL1*	-0.116 (0.049)	0.021	-0.126 (0.062)	0.045	-0.073 (0.034)	0.031	-0.120 (0.057)	0.038
cg22512531	*CRTAM*	0.164 (0.071)	0.023	0.124 (0.066)	0.066	0.168 (0.035)	2.92E-06	0.008 (0.070)	0.909
cg09084200	*VPS26B*	-0.267 (0.065)	9.50E-05	-0.201 (0.068)	0.004	-0.149 (0.045)	0.001	-0.159 (0.089)	0.076
cg07066369	*CCND2*	-0.237 (0.114)	0.041	-0.419 (0.110)	2.55E-04	-0.079 (0.064)	0.224	-0.335 (0.112)	0.004
cg07178945	*FGF23*	0.290 (0.108)	0.009	0.209 (0.099)	0.039	0.179 (0.051)	5.12E-04	0.333 (0.093)	5.62E-04
cg23193870	*PTPN6*	-0.276 (0.100)	0.007	-0.224 (0.097)	0.024	-0.172 (0.061)	0.005	-0.197 (0.104)	0.062
cg07986378	*ETV6*	-0.207 (0.100)	0.041	-0.128 (0.097)	0.192	-0.155 (0.061)	0.012	-0.274 (0.087)	0.002
cg20399616	*BCAT1*	0.286 (0.117)	0.017	0.146 (0.125)	0.244	0.222 (0.074)	0.003	0.261 (0.124)	0.039
cg02583484	*HNRNPA1*	-0.191 (0.082)	0.022	-0.103 (0.070)	0.149	-0.143 (0.046)	0.002	-0.176 (0.084)	0.039
cg21752525	-	-0.213 (0.106)	0.048	-0.177 (0.108)	0.103	-0.181 (0.064)	0.005	-0.211 (0.100)	0.039
cg01598741	*HMGA2*	-0.182 (0.111)	0.103	-0.320 (0.102)	0.002	-0.110 (0.061)	0.072	-0.338 (0.091)	3.88E-04
cg25165932	*SELPLG*	0.221 (0.103)	0.035	0.033 (0.109)	0.765	0.211 (0.064)	0.001	0.298 (0.112)	0.010
cg02801786	-	-0.021 (0.078)	0.792	0.062 (0.082)	0.454	0.246 (0.046)	2.54E-07	0.108 (0.082)	0.191
cg03844971	*MLXIP*	-0.205 (0.085)	0.019	-0.191 (0.094)	0.046	-0.172 (0.062)	0.006	-0.143 (0.098)	0.147
cg21618017	*RILPL1*	-0.140 (0.079)	0.080	-0.101 (0.071)	0.159	-0.145 (0.043)	9.49E-04	-0.137 (0.075)	0.072
cg02869235	-	-0.132 (0.090)	0.145	0.103 (0.071)	0.149	0.203 (0.042)	2.97E-06	0.145 (0.063)	0.025
cg25922751	*NCOR2*	-0.154 (0.105)	0.148	-0.316 (0.098)	0.002	-0.092 (0.057)	0.110	-0.327 (0.101)	0.002
cg06419750	*GLT1D1*	0.118 (0.096)	0.220	0.294 (0.100)	0.004	0.187 (0.064)	0.004	0.115 (0.100)	0.256
cg22574825	*FLT1*	0.062 (0.112)	0.582	0.347 (0.102)	0.001	0.219 (0.062)	4.59E-04	0.131 (0.099)	0.192
cg12836863	*BRCA2*	0.056 (0.062)	0.367	0.059 (0.056)	0.301	0.149 (0.039)	1.57E-04	0.132 (0.066)	0.050
cg02985540	-	-0.094 (0.069)	0.175	-0.122 (0.066)	0.070	-0.094 (0.036)	0.009	-0.161 (0.056)	0.005
cg03646329	*LPAR6*	-0.081 (0.095)	0.396	-0.212 (0.085)	0.014	-0.208 (0.059)	5.17E-04	-0.247 (0.087)	0.006
cg04214430	-	-0.233 (0.098)	0.020	0.008 (0.077)	0.921	-0.228 (0.063)	3.26E-04	-0.167 (0.084)	0.051
cg02003272	-	-0.119 (0.099)	0.234	-0.133 (0.084)	0.118	-0.211 (0.054)	1.28E-04	-0.161 (0.083)	0.055
cg13774342	-	-0.068 (0.102)	0.509	-0.185 (0.091)	0.047	-0.223 (0.058)	1.32E-04	-0.280 (0.093)	0.004
cg18656829	-	0.154 (0.111)	0.170	0.279 (0.113)	0.016	0.261 (0.068)	1.35E-04	0.027 (0.116)	0.815
cg20124610	*CARS2*	-0.123 (0.078)	0.116	-0.121 (0.074)	0.106	-0.192 (0.048)	8.82E-05	-0.156 (0.077)	0.047

cg00619505	*TMCO3*	0.306 (0.086)	6.17E-04	0.145 (0.076)	0.061	0.105 (0.047)	0.027	0.131 (0.084)	0.126
cg06959340	*JUB*	-0.114 (0.127)	0.372	-0.112 (0.122)	0.362	-0.297 (0.076)	1.07E-04	-0.192 (0.112)	0.091
cg02945646	*AP1G2*	-0.081 (0.064)	0.207	-0.106 (0.053)	0.047	-0.120 (0.030)	8.94E-05	-0.007 (0.057)	0.907
cg02150910	*GZMH*	0.247 (0.077)	0.002	0.098 (0.088)	0.271	0.106 (0.044)	0.017	0.147 (0.076)	0.057
cg22851561	*C14orf43*	-0.029 (0.068)	0.669	-0.102 (0.070)	0.148	-0.234 (0.051)	7.43E-06	-0.149 (0.088)	0.094
cg24996979	*C14orf43*	-0.127 (0.087)	0.150	-0.135 (0.081)	0.102	-0.163 (0.052)	0.002	-0.303 (0.095)	0.002
cg14544289	*SPTLC2*	-0.404 (0.098)	8.30E-05	-0.243 (0.094)	0.012	-0.110 (0.059)	0.064	-0.187 (0.105)	0.077
cg13679772	*FOXN3*	0.123 (0.081)	0.134	0.072 (0.075)	0.343	0.175 (0.042)	4.74E-05	0.172 (0.078)	0.031
cg01055824	*CCDC88C*	-0.215 (0.098)	0.032	-0.097 (0.084)	0.247	-0.250 (0.063)	8.82E-05	-0.066 (0.083)	0.426
cg20303561	*CCDC88C*	-0.048 (0.084)	0.570	-0.151 (0.072)	0.039	-0.152 (0.054)	0.005	-0.222 (0.074)	0.004
cg05284742	*ITPK1*	-0.214 (0.080)	0.009	-0.228 (0.081)	0.006	-0.104 (0.048)	0.033	-0.286 (0.070)	1.12E-04
cg26242531	*ZFYVE21*	0.188 (0.063)	0.004	0.162 (0.073)	0.030	0.106 (0.041)	0.011	0.191 (0.065)	0.004
cg14977938	*ZFYVE21*	0.325 (0.095)	9.42E-04	0.119 (0.100)	0.239	0.160 (0.064)	0.014	0.214 (0.101)	0.036
cg19838043	*ZFYVE21*	0.239 (0.106)	0.027	0.292 (0.093)	0.002	0.096 (0.062)	0.123	0.224 (0.082)	0.008
cg12158535	*PACS2*	0.146 (0.112)	0.196	0.308 (0.105)	0.004	0.198 (0.067)	0.003	0.172 (0.100)	0.091
cg01513913	-	-0.365 (0.121)	0.003	-0.354 (0.100)	6.47E-04	-0.365 (0.068)	1.46E-07	-0.298 (0.091)	0.002
cg13074055	-	-0.244 (0.121)	0.047	-0.334 (0.110)	0.003	-0.362 (0.072)	8.74E-07	-0.282 (0.109)	0.012
cg23594345	-	-0.288 (0.111)	0.011	-0.303 (0.108)	0.006	-0.367 (0.069)	2.23E-07	-0.286 (0.106)	0.009
cg01208318	-	-0.276 (0.108)	0.012	-0.280 (0.107)	0.010	-0.362 (0.066)	1.04E-07	-0.300 (0.092)	0.002
cg14387626	-	-0.145 (0.068)	0.035	-0.137 (0.051)	0.009	-0.227 (0.042)	1.43E-07	0.025 (0.060)	0.674
cg27113548	-	-0.205 (0.126)	0.107	-0.096 (0.116)	0.412	-0.354 (0.069)	4.89E-07	-0.321 (0.097)	0.001
cg00980649	-	-0.143 (0.112)	0.204	-0.181 (0.114)	0.115	-0.340 (0.061)	5.20E-08	-0.306 (0.094)	0.002
cg03603381	*RASGRP1*	-0.268 (0.090)	0.004	-0.145 (0.119)	0.228	-0.163 (0.062)	0.009	-0.176 (0.106)	0.099
cg14428590	*FSIP1*	-0.057 (0.098)	0.560	-0.193 (0.084)	0.024	-0.160 (0.056)	0.004	-0.213 (0.086)	0.016
cg24687805	*RAB27A*	0.129 (0.085)	0.134	0.109 (0.065)	0.099	0.182 (0.057)	0.002	0.218 (0.085)	0.012
cg22777952	*FOXB1*	0.093 (0.089)	0.302	0.183 (0.101)	0.074	0.174 (0.057)	0.003	0.252 (0.102)	0.016
cg15451980	*RORA*	0.138 (0.076)	0.073	0.092 (0.091)	0.311	0.125 (0.043)	0.004	0.152 (0.064)	0.020
cg26971042	*TLE3*	-0.154 (0.113)	0.176	-0.223 (0.109)	0.043	-0.153 (0.059)	0.010	-0.286 (0.097)	0.004

cg09747445	*TLE3*	-0.131 (0.081)	0.108	-0.142 (0.068)	0.038	-0.139 (0.047)	0.004	-0.153 (0.073)	0.041
cg02384859	*ARID3B*	0.156 (0.067)	0.023	0.151 (0.093)	0.111	0.152 (0.044)	5.79E-04	0.099 (0.070)	0.161
cg18946533	*SH2D7*	0.309 (0.097)	0.002	0.021 (0.077)	0.788	0.217 (0.053)	5.56E-05	0.001 (0.106)	0.991
cg14096889	*ADAMTSL3*	0.140 (0.097)	0.151	0.170 (0.105)	0.110	0.167 (0.063)	0.008	0.281 (0.098)	0.005
cg20664238	*NTRK3*	0.162 (0.084)	0.057	0.164 (0.091)	0.075	0.173 (0.053)	0.001	0.112 (0.100)	0.265
cg23161492	*ANPEP*	-0.393 (0.100)	1.66E-04	-0.213 (0.105)	0.046	-0.271 (0.064)	3.34E-05	-0.404 (0.097)	8.01E-05
cg14858469	*NR2F2*	0.142 (0.119)	0.236	0.056 (0.112)	0.619	0.297 (0.066)	9.53E-06	0.117 (0.107)	0.278
cg07779120	*IGF1R*	0.254 (0.105)	0.018	0.063 (0.119)	0.600	0.210 (0.067)	0.002	0.499 (0.107)	1.08E-05
cg04755561	*PKMYT1*	-0.045 (0.079)	0.570	-0.245 (0.062)	1.50E-04	-0.140 (0.051)	0.006	-0.107 (0.070)	0.132
cg06321596	*XYLT1*	-0.211 (0.103)	0.044	-0.131 (0.089)	0.142	-0.252 (0.060)	3.57E-05	-0.199 (0.087)	0.025
cg02304156	*ATP2A1*	0.127 (0.095)	0.182	0.029 (0.069)	0.671	0.214 (0.047)	8.72E-06	0.065 (0.090)	0.474
cg07069636	-	-0.102 (0.072)	0.160	-0.207 (0.072)	0.005	-0.113 (0.038)	0.003	-0.228 (0.081)	0.006
cg04528720	-	0.181 (0.075)	0.018	0.174 (0.083)	0.038	0.132 (0.041)	0.001	0.064 (0.070)	0.359
cg08126789	*USP10*	0.205 (0.119)	0.088	0.117 (0.105)	0.267	0.185 (0.059)	0.002	0.198 (0.099)	0.048
cg01383486	*GINS2*	-0.281 (0.088)	0.002	-0.146 (0.082)	0.079	-0.128 (0.045)	0.004	-0.050 (0.071)	0.481
cg04887172	*CBFA2T3*	-0.141 (0.064)	0.032	-0.190 (0.058)	0.002	-0.150 (0.042)	3.99E-04	0.057 (0.086)	0.509
cg01107178	*ANKRD11*	0.092 (0.067)	0.171	0.100 (0.060)	0.098	0.098 (0.040)	0.015	0.200 (0.059)	0.001
cg02352716	-	0.213 (0.079)	0.008	0.153 (0.089)	0.091	0.102 (0.044)	0.020	0.115 (0.065)	0.081
cg15380836	*RILP*	-0.203 (0.054)	3.24E-04	-0.061 (0.051)	0.230	-0.043 (0.028)	0.124	-0.196 (0.054)	5.04E-04
cg03877174	*KIF1C*	0.177 (0.072)	0.016	0.182 (0.072)	0.013	0.095 (0.044)	0.033	0.244 (0.065)	3.36E-04
cg05460226	*PIK3R5*	-0.096 (0.090)	0.290	-0.159 (0.081)	0.052	-0.128 (0.049)	0.010	-0.244 (0.069)	7.07E-04
cg02018337	*GAS7*	-0.185 (0.061)	0.003	-0.015 (0.057)	0.791	-0.179 (0.036)	9.79E-07	-0.034 (0.054)	0.529
cg18150958	*RPL23A*	-0.172 (0.116)	0.143	-0.213 (0.098)	0.034	-0.234 (0.069)	7.94E-04	-0.285 (0.105)	0.008
cg18960216	*TIAF1*	0.255 (0.092)	0.007	-0.019 (0.092)	0.840	0.201 (0.045)	1.34E-05	0.213 (0.078)	0.008
cg19572487	*RARA*	-0.233 (0.092)	0.013	-0.264 (0.087)	0.003	-0.265 (0.057)	6.09E-06	-0.499 (0.088)	1.86E-07
cg23673974	*TBKBP1*	0.132 (0.065)	0.045	0.095 (0.078)	0.229	0.132 (0.038)	5.11E-04	0.094 (0.075)	0.210
cg22807449	*HOXB2*	-0.007 (0.097)	0.940	-0.274 (0.081)	0.001	-0.238 (0.057)	4.56E-05	-0.055 (0.104)	0.599
cg07465627	*STXBP4*	-0.138 (0.074)	0.066	-0.202 (0.068)	0.004	-0.184 (0.041)	1.25E-05	-0.128 (0.078)	0.105

cg08591265	*SFRS1*	-0.179 (0.087)	0.044	-0.205 (0.087)	0.021	-0.163 (0.043)	1.99E-04	-0.074 (0.070)	0.297
cg05248618	*CA4*	0.335 (0.112)	0.004	0.189 (0.121)	0.123	0.166 (0.068)	0.016	0.227 (0.110)	0.042
cg07827420	*SEPT9*	-0.164 (0.123)	0.186	-0.165 (0.114)	0.151	-0.182 (0.073)	0.014	-0.405 (0.114)	6.63E-04
cg07324245	*SEPT9*	-0.130 (0.053)	0.017	-0.081 (0.046)	0.079	-0.113 (0.031)	3.49E-04	-0.105 (0.065)	0.110
cg05080154	*SALL3*	0.229 (0.082)	0.007	0.132 (0.098)	0.181	0.254 (0.059)	2.57E-05	0.170 (0.084)	0.045
cg00073090	-	-0.104 (0.070)	0.142	-0.135 (0.069)	0.054	-0.185 (0.048)	1.34E-04	-0.232 (0.071)	0.001
cg15187398	*MOBKL2A*	-0.269 (0.077)	7.91E-04	-0.191 (0.107)	0.079	-0.114 (0.058)	0.049	-0.355 (0.097)	4.16E-04
cg07381806	*MOBKL2A*	-0.232 (0.114)	0.045	-0.068 (0.112)	0.544	-0.192 (0.064)	0.003	-0.256 (0.105)	0.017
cg00378510	*LINGO3*	-0.294 (0.098)	0.004	-0.342 (0.107)	0.002	-0.078 (0.063)	0.213	-0.248 (0.099)	0.014
cg01294327	*LINGO3*	-0.368 (0.107)	8.82E-04	-0.472 (0.104)	2.01E-05	-0.109 (0.064)	0.090	-0.207 (0.111)	0.065
cg14074174	*SNAPC2*	-0.246 (0.078)	0.002	-0.216 (0.090)	0.019	-0.108 (0.053)	0.044	-0.129 (0.082)	0.119
cg03172931	-	0.192 (0.109)	0.082	0.206 (0.097)	0.037	0.161 (0.055)	0.004	0.221 (0.090)	0.016
cg11621113	*MORG1*	-0.184 (0.075)	0.017	-0.114 (0.052)	0.032	-0.087 (0.037)	0.018	-0.238 (0.073)	0.002
cg05339037	-	-0.213 (0.101)	0.038	-0.086 (0.100)	0.396	-0.183 (0.055)	9.09E-04	-0.173 (0.094)	0.068
cg14588779	*AKAP8L*	0.002 (0.070)	0.972	-0.268 (0.062)	3.87E-05	-0.112 (0.034)	0.001	-0.078 (0.067)	0.249
cg09686308	*CIB3*	0.223 (0.087)	0.013	0.262 (0.097)	0.008	0.139 (0.054)	0.011	0.126 (0.103)	0.225
cg03636183	*F2RL3*	-0.538 (0.103)	1.23E-06	-0.376 (0.101)	3.83E-04	-0.445 (0.055)	2.93E-14	-0.549 (0.083)	2.89E-09
cg15159987	*CPAMD8*	-0.123 (0.083)	0.144	-0.037 (0.085)	0.668	-0.199 (0.055)	3.65E-04	-0.233 (0.081)	0.005
cg22678402	*FAM125A*	-0.180 (0.064)	0.006	-0.036 (0.054)	0.515	-0.087 (0.028)	0.002	-0.125 (0.069)	0.076
cg23973524	*CRTC1*	0.408 (0.098)	7.96E-05	0.121 (0.078)	0.126	0.178 (0.058)	0.002	0.135 (0.085)	0.118
cg21473814	*CRTC1*	0.435 (0.109)	1.32E-04	0.122 (0.100)	0.227	0.128 (0.065)	0.048	0.232 (0.090)	0.012
cg01447828	*PRX*	0.174 (0.106)	0.107	0.135 (0.101)	0.185	0.285 (0.065)	1.51E-05	0.259 (0.105)	0.016
cg15393221	*PRX*	0.102 (0.087)	0.243	0.050 (0.082)	0.545	0.214 (0.048)	1.37E-05	0.289 (0.075)	2.45E-04
cg13668129	*HNRNPUL 1*	-0.044 (0.060)	0.472	-0.101 (0.044)	0.024	-0.116 (0.029)	1.00E-04	-0.076 (0.062)	0.221
cg10126923	*NKG7*	0.003 (0.057)	0.960	0.107 (0.060)	0.078	0.120 (0.033)	2.68E-04	0.231 (0.062)	3.30E-04
cg12916723	*NKG7*	0.243 (0.117)	0.040	0.188 (0.109)	0.088	0.134 (0.056)	0.018	0.272 (0.103)	0.010
cg01500140	*LIM2*	0.091 (0.055)	0.099	0.083 (0.061)	0.177	0.095 (0.030)	0.002	0.171 (0.053)	0.002

cg03217253	*ZNF677*	0.213 (0.092)	0.023	0.172 (0.106)	0.107	0.253 (0.060)	3.10E-05	0.150 (0.105)	0.155
cg21733502	*ZSCAN5B*	-0.268 (0.104)	0.012	-0.166 (0.103)	0.110	-0.155 (0.071)	0.030	-0.256 (0.111)	0.024
cg05007126	*SDCBP2*	-0.062 (0.083)	0.456	-0.100 (0.074)	0.184	-0.164 (0.047)	5.15E-04	-0.169 (0.079)	0.035
cg13724496	*BMP2*	0.048 (0.107)	0.655	0.248 (0.102)	0.017	0.240 (0.059)	6.04E-05	0.102 (0.103)	0.324
cg15207742	*RIMS4*	-0.055 (0.106)	0.602	0.310 (0.095)	0.002	0.275 (0.066)	4.17E-05	0.199 (0.092)	0.033
cg20344344	-	-0.232 (0.090)	0.012	-0.024 (0.085)	0.783	-0.161 (0.051)	0.002	-0.216 (0.081)	0.009
cg12303084	*ZMYND8*	-0.103 (0.070)	0.144	-0.085 (0.056)	0.135	-0.110 (0.035)	0.002	-0.146 (0.072)	0.046
cg07339236	*ATP9A*	-0.274 (0.086)	0.002	-0.270 (0.100)	0.009	-0.248 (0.060)	5.20E-05	-0.240 (0.101)	0.021
cg26736540	*TFAP2C*	0.012 (0.088)	0.894	0.136 (0.106)	0.203	0.187 (0.059)	0.002	0.367 (0.104)	6.65E-04
cg06135139	*BHLHE23*	0.152 (0.108)	0.162	-0.079 (0.122)	0.516	0.315 (0.068)	5.41E-06	0.305 (0.117)	0.011
cg15892280	*ETS2*	-0.117 (0.058)	0.049	-0.071 (0.042)	0.097	-0.142 (0.040)	4.38E-04	-0.068 (0.057)	0.236
cg23110422	*ETS2*	-0.357 (0.101)	7.04E-04	-0.164 (0.112)	0.147	-0.229 (0.071)	0.001	-0.282 (0.102)	0.007
cg10204884	*PCBP3*	-0.201 (0.095)	0.037	-0.223 (0.081)	0.007	-0.139 (0.058)	0.017	-0.122 (0.089)	0.176
cg14785479	*SCARF2*	0.159 (0.067)	0.020	0.094 (0.051)	0.071	0.121 (0.039)	0.002	0.135 (0.052)	0.012
cg08548559	*PIK3IP1*	-0.231 (0.083)	0.007	-0.197 (0.079)	0.014	-0.077 (0.052)	0.144	-0.234 (0.087)	0.009
cg02532700	*NCF4*	-0.251 (0.109)	0.024	-0.210 (0.112)	0.063	-0.182 (0.063)	0.004	-0.299 (0.114)	0.010
cg26364091	*CHADL*	-0.180 (0.108)	0.099	-0.266 (0.113)	0.021	-0.180 (0.065)	0.006	-0.195 (0.108)	0.075
cg06008724	*PHF21B*	0.040 (0.105)	0.706	0.226 (0.100)	0.027	0.220 (0.056)	1.04E-04	0.115 (0.080)	0.156
cg04198308	*FAM19A5*	0.270 (0.098)	0.007	0.221 (0.112)	0.052	0.223 (0.064)	6.09E-04	0.018 (0.103)	0.862

Table S3. The median methylation values (inter quartile range) of the 318 smoking-related CpGs in never, former and current smokers.

CpG	Gene	Methylation values			Former vs. never		Current vs. never	
		Current smoker (*n* = 273)	Former smoker (*n* = 64)	Never smoker (*n* = 259)	Effect (s.e.)[a]	*P*	Effect (s.e.)[a]	*P*
cg05575921	*AHRR*	0.641 (0.143)	0.729 (0.104)	0.795 (0.062)	-0.778	1.33E-12	-1.342	3.83E-67
cg23576855	*AHRR*	0.515 (0.134)	0.591 (0.091)	0.663 (0.065)	-0.685	6.75E-10	-1.302	1.61E-62
cg21566642	-	0.352 (0.093)	0.398 (0.076)	0.442 (0.069)	-0.581	2.00E-06	-1.103	1.09E-40
cg05951221	-	0.286 (0.074)	0.291 (0.062)	0.351 (0.057)	-0.827	1.33E-11	-1.044	1.09E-37
cg26703534	*AHRR*	0.551 (0.060)	0.575 (0.077)	0.592 (0.059)	-0.369	2.52E-03	-0.793	4.22E-23
cg03636183	*F2RL3*	0.534 (0.101)	0.553 (0.089)	0.605 (0.070)	-0.628	3.36E-07	-1.000	3.69E-34
cg01940273	-	0.468 (0.064)	0.491 (0.076)	0.539 (0.060)	-0.567	2.08E-06	-1.134	3.68E-44
cg25648203	*AHRR*	0.690 (0.063)	0.709 (0.049)	0.718 (0.059)	-0.229	0.077	-0.693	1.66E-16
cg22132788	*MYO1G*	0.913 (0.062)	0.889 (0.051)	0.876 (0.068)	0.184	0.174	0.676	1.14E-14
cg21161138	*AHRR*	0.629 (0.065)	0.657 (0.055)	0.669 (0.048)	-0.261	0.043	-0.814	9.00E-22
cg03329539	-	0.329 (0.068)	0.339 (0.070)	0.363 (0.077)	-0.333	0.010	-0.603	4.83E-13
cg06126421	-	0.629 (0.120)	0.602 (0.108)	0.692 (0.084)	-0.758	1.39E-10	-0.835	2.68E-27
cg01513913	-	0.371 (0.064)	0.381 (0.056)	0.391 (0.064)	-0.082	0.522	-0.509	5.97E-10
cg12803068	*MYO1G*	0.768 (0.096)	0.752 (0.093)	0.718 (0.106)	0.291	0.032	0.657	6.57E-14
cg21611682	*LRP5*	0.501 (0.041)	0.504 (0.055)	0.532 (0.036)	-0.705	5.32E-08	-0.843	1.74E-23
cg00501876	*CSRNP1*	0.545 (0.045)	0.553 (0.039)	0.562 (0.042)	-0.175	0.188	-0.506	2.91E-09
cg14817490	*AHRR*	0.200 (0.066)	0.236 (0.086)	0.236 (0.066)	-0.033	0.803	-0.591	3.38E-12
cg01208318	-	0.426 (0.114)	0.478 (0.177)	0.460 (0.108)	-0.018	0.890	-0.501	4.88E-09
cg06811467	*ATP8B2*	0.284 (0.079)	0.284 (0.078)	0.294 (0.079)	-0.345	4.51E-03	-0.319	3.39E-05
cg24540678	-	0.200 (0.058)	0.217 (0.081)	0.222 (0.071)	-0.216	0.103	-0.406	1.46E-06
cg23594345	-	0.442 (0.115)	0.481 (0.148)	0.484 (0.107)	0.041	0.758	-0.519	1.30E-09
cg02018337	*GAS7*	0.251 (0.081)	0.234 (0.063)	0.263 (0.095)	-0.296	8.40E-03	-0.228	0.001
cg07573717	*CAPZB*	0.321 (0.068)	0.311 (0.064)	0.328 (0.065)	-0.221	0.066	-0.288	1.58E-04
cg14387626	-	0.345 (0.052)	0.334 (0.040)	0.356 (0.048)	-0.459	2.25E-04	-0.368	3.07E-06
cg23161492	*ANPEP*	0.263 (0.059)	0.280 (0.066)	0.290 (0.047)	-0.252	0.057	-0.647	4.17E-14

cg03991871	*AHRR*	0.783 (0.047)	0.797 (0.056)	0.815 (0.037)	-0.294	0.026	-0.717	6.00E-17
cg00980649	-	0.332 (0.084)	0.342 (0.124)	0.342 (0.106)	0.029	0.830	-0.312	2.09E-04
cg11660018	*PRSS23*	0.448 (0.058)	0.450 (0.070)	0.488 (0.061)	-0.721	1.27E-09	-0.816	6.32E-26
cg03440944	*C7orf40*	0.643 (0.056)	0.660 (0.063)	0.655 (0.058)	0.034	0.776	-0.284	2.04E-04
cg19089201	*MYO1G*	0.832 (0.060)	0.830 (0.056)	0.812 (0.060)	0.221	0.117	0.378	2.48E-05
cg23079012	-	0.894 (0.036)	0.898 (0.031)	0.904 (0.031)	-0.219	0.104	-0.525	1.25E-09
cg13074055	-	0.545 (0.103)	0.553 (0.120)	0.571 (0.104)	-0.021	0.879	-0.377	1.65E-05
cg26542660	*CEP135*	0.157 (0.067)	0.154 (0.060)	0.161 (0.064)	-0.231	0.055	-0.291	1.48E-04
cg22512531	*CRTAM*	0.704 (0.077)	0.722 (0.076)	0.688 (0.083)	0.322	3.65E-03	0.219	0.002
cg15164194	*WDFY4*	0.262 (0.083)	0.240 (0.068)	0.265 (0.075)	-0.318	6.54E-03	-0.192	0.009
cg27113548	-	0.641 (0.076)	0.647 (0.083)	0.667 (0.072)	-0.115	0.381	-0.361	1.48E-05
cg19572487	*RARA*	0.468 (0.082)	0.488 (0.096)	0.503 (0.067)	-0.152	0.240	-0.588	1.58E-12
cg19859270	*GPR15*	0.816 (0.031)	0.820 (0.029)	0.831 (0.026)	-0.345	0.012	-0.568	1.07E-10
cg18960216	*TIAF1*	0.668 (0.061)	0.670 (0.046)	0.658 (0.057)	0.186	0.104	0.266	2.41E-04
cg07978738	*ABLIM1*	0.213 (0.061)	0.214 (0.057)	0.221 (0.067)	-0.043	0.728	-0.320	4.17E-05
cg25189904	*GNG12*	0.387 (0.093)	0.420 (0.138)	0.434 (0.096)	-0.509	1.21E-04	-0.616	4.27E-13
cg04232128	*TMEM173*	0.222 (0.076)	0.208 (0.061)	0.224 (0.079)	-0.183	0.118	-0.231	0.002
cg23916896	*AHRR*	0.218 (0.051)	0.225 (0.062)	0.247 (0.058)	-0.355	8.88E-03	-0.624	6.54E-13
cg07339236	*ATP9A*	0.167 (0.055)	0.165 (0.048)	0.189 (0.049)	-0.491	4.99E-05	-0.578	1.04E-13
cg18946533	*SH2D7*	0.565 (0.048)	0.554 (0.052)	0.555 (0.047)	-0.065	0.634	0.227	0.009
cg05080154	*SALL3*	0.117 (0.104)	0.110 (0.066)	0.093 (0.078)	0.135	0.283	0.381	2.02E-06
cg12806681	*AHRR*	0.818 (0.033)	0.827 (0.033)	0.836 (0.030)	-0.298	0.027	-0.593	7.80E-12
cg09088988	*STK32A*	0.128 (0.062)	0.101 (0.069)	0.110 (0.060)	0.020	0.873	0.330	5.09E-05
cg26850624	*AHRR*	0.757 (0.052)	0.748 (0.056)	0.745 (0.051)	0.310	0.013	0.407	3.15E-07
cg23771366	*PRSS23*	0.385 (0.070)	0.384 (0.068)	0.419 (0.058)	-0.469	1.83E-04	-0.622	1.34E-14
cg04016086	*COBL*	0.077 (0.038)	0.066 (0.024)	0.069 (0.029)	-0.131	0.339	0.353	4.89E-05
cg10750182	*CDH23*	0.522 (0.048)	0.515 (0.043)	0.543 (0.056)	-0.533	1.87E-05	-0.478	1.74E-09
cg01692968	-	0.287 (0.064)	0.271 (0.042)	0.324 (0.068)	-0.796	6.17E-11	-0.671	4.44E-18
cg09578155	*LRP5*	0.392 (0.051)	0.386 (0.052)	0.408 (0.043)	-0.417	9.60E-04	-0.414	2.46E-07
cg23186333	*CD44*	0.319 (0.066)	0.316 (0.066)	0.332 (0.072)	-0.092	0.472	-0.325	4.50E-05
cg09084200	*VPS26B*	0.281 (0.065)	0.286 (0.074)	0.295 (0.063)	0.020	0.877	-0.337	4.73E-05
cg25305703	-	0.556 (0.100)	0.555 (0.120)	0.589 (0.077)	-0.453	6.47E-04	-0.441	1.71E-07

cg14556677	*NEK6*	0.253 (0.078)	0.245 (0.074)	0.263 (0.073)	-0.283	0.018	-0.242	0.001
cg03217253	*ZNF677*	0.107 (0.058)	0.090 (0.061)	0.094 (0.058)	0.058	0.665	0.351	3.43E-05
cg14663208	*HIVEP3*	0.728 (0.060)	0.734 (0.057)	0.713 (0.063)	0.229	0.038	0.318	5.59E-06
cg14753356	-	0.352 (0.093)	0.327 (0.075)	0.390 (0.105)	-0.620	1.30E-07	-0.535	9.26E-13
cg09935388	*GFI1*	0.700 (0.070)	0.711 (0.067)	0.732 (0.061)	-0.451	7.00E-04	-0.628	2.12E-13
cg08595501	*IQGAP2*	0.568 (0.076)	0.579 (0.065)	0.599 (0.075)	-0.218	0.116	-0.432	1.05E-06
cg07465627	*STXBP4*	0.302 (0.063)	0.312 (0.066)	0.316 (0.072)	-0.166	0.215	-0.306	3.31E-04
cg15693483	*C7orf50*	0.432 (0.056)	0.418 (0.059)	0.450 (0.061)	-0.481	4.12E-05	-0.441	3.45E-09
cg22644321	*TRIB1*	0.157 (0.054)	0.155 (0.054)	0.165 (0.061)	-0.208	0.059	-0.321	4.90E-06
cg27537125	-	0.168 (0.057)	0.180 (0.086)	0.180 (0.069)	0.013	0.922	-0.278	7.31E-04
cg19708306	-	0.824 (0.061)	0.822 (0.056)	0.812 (0.075)	0.272	0.024	0.247	0.001
cg09471611	-	0.648 (0.051)	0.647 (0.054)	0.637 (0.057)	0.077	0.523	0.230	0.003
cg11902777	*AHRR*	0.067 (0.021)	0.072 (0.023)	0.075 (0.027)	-0.107	0.436	-0.429	9.84E-07
cg06135139	*BHLHE23*	0.180 (0.034)	0.173 (0.037)	0.169 (0.030)	0.238	0.086	0.466	1.38E-07
cg09257526	*IL6R*	0.267 (0.062)	0.260 (0.061)	0.274 (0.065)	-0.281	0.018	-0.257	6.41E-04
cg23973524	*CRTC1*	0.597 (0.076)	0.560 (0.101)	0.566 (0.081)	0.081	0.540	0.498	4.45E-09
cg02560388	-	0.190 (0.055)	0.175 (0.058)	0.197 (0.055)	-0.279	0.014	-0.303	2.48E-05
cg06321596	*XYLT1*	0.339 (0.072)	0.324 (0.059)	0.354 (0.078)	-0.397	2.88E-03	-0.412	1.15E-06
cg08035323	-	0.258 (0.080)	0.235 (0.086)	0.252 (0.095)	-0.290	0.031	0.066	0.438
cg01447828	*PRX*	0.522 (0.051)	0.496 (0.051)	0.513 (0.053)	-0.348	9.70E-03	0.301	4.00E-04
cg16619991	*ITGA1*	0.490 (0.085)	0.495 (0.087)	0.491 (0.078)	0.066	0.625	-0.091	0.287
cg17426273	*NEBL*	0.122 (0.054)	0.112 (0.054)	0.111 (0.058)	-0.024	0.849	0.277	5.73E-04
cg09044186	*APOA5*	0.931 (0.027)	0.931 (0.020)	0.931 (0.025)	-0.064	0.644	0.133	0.133
cg02304156	*ATP2A1*	0.707 (0.056)	0.710 (0.053)	0.702 (0.064)	0.149	0.241	0.288	3.49E-04
cg25325005	*PLEC1*	0.715 (0.075)	0.740 (0.069)	0.723 (0.070)	0.308	0.020	-0.131	0.117
cg19254163	*GPR44*	0.571 (0.059)	0.569 (0.071)	0.595 (0.054)	-0.544	5.28E-06	-0.495	8.01E-11
cg01055824	*CCDC88C*	0.843 (0.047)	0.833 (0.058)	0.848 (0.046)	-0.211	0.122	-0.230	0.008
cg23110422	*ETS2*	0.767 (0.032)	0.771 (0.041)	0.781 (0.032)	-0.399	2.58E-03	-0.601	1.69E-12
cg11229399	-	0.104 (0.045)	0.099 (0.046)	0.097 (0.043)	0.034	0.803	0.286	8.19E-04
cg27122888	*NRXN2*	0.126 (0.035)	0.131 (0.044)	0.138 (0.033)	-0.054	0.648	-0.339	6.85E-06
cg22674699	*HOXD9*	0.166 (0.113)	0.129 (0.115)	0.140 (0.094)	0.040	0.766	0.417	1.22E-06
cg14858469	*NR2F2*	0.102 (0.019)	0.096 (0.019)	0.097 (0.022)	-0.042	0.760	0.273	0.002

cg25853622	*LPP*	0.352 (0.113)	0.317 (0.090)	0.361 (0.115)	-0.396	3.38E-04	-0.192	0.006
cg23572908	*VIPR2*	0.147 (0.039)	0.136 (0.026)	0.135 (0.036)	0.077	0.572	0.444	2.90E-07
cg15393221	*PRX*	0.355 (0.055)	0.319 (0.061)	0.343 (0.055)	-0.401	3.12E-03	0.153	0.074
cg02801786	-	0.161 (0.064)	0.146 (0.057)	0.147 (0.072)	-0.043	0.724	0.320	3.48E-05
cg07178945	*FGF23*	0.370 (0.058)	0.368 (0.082)	0.350 (0.064)	0.248	0.070	0.383	1.06E-05
cg04198308	*FAM19A5*	0.057 (0.027)	0.051 (0.026)	0.051 (0.022)	-0.031	0.816	0.361	2.22E-05
cg07324245	*SEPT9*	0.659 (0.075)	0.678 (0.073)	0.665 (0.075)	0.314	0.010	-0.079	0.306
cg25114611	*FKBP5*	0.310 (0.076)	0.306 (0.073)	0.320 (0.068)	-0.199	0.092	-0.279	2.00E-04
cg23924887	*ATP8B2*	0.079 (0.028)	0.076 (0.034)	0.087 (0.031)	-0.216	0.113	-0.260	0.003
cg04214430	-	0.855 (0.031)	0.861 (0.028)	0.859 (0.027)	0.120	0.373	-0.175	0.041
cg19719391	-	0.546 (0.068)	0.519 (0.082)	0.532 (0.06)	-0.153	0.241	0.163	0.048
cg05396397	*NPPA*	0.508 (0.063)	0.480 (0.072)	0.485 (0.061)	-0.162	0.238	0.441	4.48E-07
cg13679772	*FOXN3*	0.468 (0.075)	0.463 (0.086)	0.458 (0.075)	-0.012	0.927	0.235	0.004
cg08591265	*SFRS1*	0.624 (0.089)	0.643 (0.096)	0.626 (0.088)	0.291	0.018	-0.028	0.714
cg15474579	*CDKN1A*	0.549 (0.094)	0.538 (0.091)	0.568 (0.095)	-0.365	2.12E-03	-0.406	8.23E-08
cg04551776	*AHRR*	0.705 (0.051)	0.733 (0.042)	0.722 (0.049)	0.070	0.598	-0.416	9.86E-07
cg20124610	*CARS2*	0.481 (0.082)	0.492 (0.104)	0.494 (0.079)	0.072	0.585	-0.232	0.006
cg11207515	*CNTNAP2*	0.389 (0.079)	0.391 (0.096)	0.349 (0.078)	0.536	5.63E-05	0.638	8.54E-14
cg16783744	*DPYS*	0.192 (0.071)	0.185 (0.060)	0.183 (0.069)	0.054	0.673	0.319	7.73E-05
cg04887172	*CBFA2T3*	0.285 (0.089)	0.263 (0.108)	0.289 (0.083)	-0.124	0.290	-0.089	0.228
cg25949550	*CNTNAP2*	0.122 (0.046)	0.130 (0.064)	0.134 (0.048)	-0.188	0.164	-0.340	7.41E-05
cg09338136	*AHRR*	0.218 (0.053)	0.224 (0.062)	0.225 (0.055)	-0.231	0.081	-0.337	6.24E-05
cg02451831	*KIAA0087*	0.679 (0.038)	0.678 (0.038)	0.687 (0.035)	-0.265	0.058	-0.306	5.57E-04
cg09613161	*COBL*	0.084 (0.040)	0.078 (0.029)	0.078 (0.038)	0.079	0.558	0.301	4.68E-04
cg02384859	*ARID3B*	0.743 (0.076)	0.758 (0.074)	0.728 (0.077)	0.386	3.16E-04	0.277	4.29E-05
cg00778858	*MTUS1*	0.057 (0.018)	0.055 (0.017)	0.054 (0.017)	0.124	0.354	0.269	0.002
cg22851561	*C14orf43*	0.483 (0.085)	0.510 (0.095)	0.508 (0.088)	-0.023	0.862	-0.329	8.09E-05
cg09022230	*TNRC18*	0.670 (0.058)	0.669 (0.071)	0.699 (0.065)	-0.589	3.75E-06	-0.648	2.36E-15
cg11554391	*AHRR*	0.156 (0.043)	0.167 (0.038)	0.170 (0.042)	-0.211	0.112	-0.475	2.03E-08
cg09373037	*SYT15*	0.121 (0.059)	0.111 (0.047)	0.109 (0.052)	0.106	0.430	0.398	3.54E-06
cg01955533	*CDKN1A*	0.238 (0.079)	0.218 (0.067)	0.242 (0.081)	-0.262	0.020	-0.230	0.001
cg15700587	*MIR548I4*	0.690 (0.046)	0.694 (0.046)	0.681 (0.049)	0.342	4.78E-03	0.340	9.85E-06

cg06644428	-	0.101 (0.052)	0.104 (0.053)	0.122 (0.058)	-0.596	4.10E-06	-0.523	2.15E-10
cg09156233	*BMPR1B*	0.073 (0.042)	0.057 (0.035)	0.062 (0.033)	-0.037	0.778	0.396	2.28E-06
cg20164601	-	0.514 (0.069)	0.528 (0.087)	0.513 (0.087)	0.122	0.393	-0.079	0.380
cg08617970	*VARS2*	0.889 (0.017)	0.888 (0.017)	0.892 (0.015)	-0.235	0.094	-0.257	0.004
cg00073090	-	0.316 (0.054)	0.315 (0.059)	0.338 (0.063)	-0.506	5.89E-05	-0.588	3.56E-13
cg16382047	*GPR55*	0.306 (0.079)	0.294 (0.057)	0.320 (0.088)	-0.427	1.87E-04	-0.318	1.07E-05
cg04180046	*MYO1G*	0.506 (0.078)	0.508 (0.074)	0.480 (0.069)	0.185	0.174	0.370	1.89E-05
cg24090911	*AHRR*	0.671 (0.053)	0.681 (0.064)	0.690 (0.042)	-0.176	0.174	-0.543	7.37E-11
cg22678402	*FAM125A*	0.709 (0.094)	0.697 (0.105)	0.702 (0.09)	-0.050	0.694	-0.066	0.410
cg15892280	*ETS2*	0.375 (0.064)	0.364 (0.048)	0.389 (0.056)	-0.424	3.30E-04	-0.352	2.63E-06
cg05329352	*ADRA2A*	0.518 (0.089)	0.546 (0.095)	0.563 (0.095)	-0.221	0.096	-0.486	1.07E-08
cg02945646	*AP1G2*	0.116 (0.046)	0.117 (0.045)	0.121 (0.049)	-0.072	0.584	-0.141	0.088
cg01383486	*GINS2*	0.748 (0.085)	0.744 (0.092)	0.749 (0.083)	-0.134	0.312	-0.075	0.372
cg23673974	*TBKBP1*	0.531 (0.074)	0.536 (0.093)	0.521 (0.08)	0.035	0.790	0.211	0.012
cg04211179	*ZBTB17*	0.484 (0.076)	0.513 (0.108)	0.492 (0.081)	0.248	0.048	-0.152	0.056
cg20344344	-	0.810 (0.032)	0.815 (0.038)	0.816 (0.035)	-0.031	0.822	-0.171	0.048
cg18656829	-	0.076 (0.021)	0.072 (0.021)	0.071 (0.019)	-0.129	0.355	0.307	5.16E-04
cg14316231	*MYST3*	0.434 (0.053)	0.432 (0.067)	0.450 (0.048)	-0.363	7.64E-03	-0.409	2.21E-06
cg04528720	-	0.679 (0.061)	0.689 (0.069)	0.667 (0.065)	0.314	4.07E-03	0.247	3.62E-04
cg20949306	*RAB3GAP1*	0.910 (0.026)	0.907 (0.024)	0.903 (0.027)	0.146	0.227	0.417	7.54E-08
cg17094249	-	0.212 (0.045)	0.217 (0.051)	0.221 (0.046)	-0.052	0.710	-0.309	4.84E-04
cg08129092	*INTS3*	0.178 (0.080)	0.153 (0.051)	0.169 (0.078)	-0.256	0.049	0.085	0.302
cg09858188	-	0.171 (0.074)	0.148 (0.063)	0.156 (0.07)	-0.067	0.612	0.311	1.89E-04
cg05339037	-	0.385 (0.042)	0.391 (0.042)	0.397 (0.039)	-0.251	0.069	-0.444	4.27E-07
cg24859433	-	0.754 (0.045)	0.751 (0.053)	0.772 (0.037)	-0.526	7.06E-05	-0.493	5.01E-09
cg02003272	-	0.828 (0.043)	0.838 (0.036)	0.829 (0.049)	0.310	0.019	-0.090	0.280
cg14624207	*LRP5*	0.483 (0.056)	0.481 (0.069)	0.503 (0.056)	-0.349	5.14E-03	-0.487	9.90E-10
cg10351287	*STK32B*	0.083 (0.033)	0.073 (0.031)	0.077 (0.033)	-0.007	0.956	0.310	3.23E-04
cg25165932	*SELPLG*	0.051 (0.011)	0.049 (0.012)	0.048 (0.012)	0.180	0.204	0.276	0.002
cg07779120	*IGF1R*	0.056 (0.026)	0.048 (0.018)	0.049 (0.019)	-0.093	0.500	0.419	1.72E-06
cg20460771	*PTAFR*	0.673 (0.076)	0.693 (0.074)	0.654 (0.076)	0.299	6.54E-03	0.327	2.71E-06
cg04158878	-	0.613 (0.048)	0.619 (0.048)	0.601 (0.047)	0.345	7.18E-03	0.359	1.05E-05

cg13279811	-	0.196 (0.055)	0.193 (0.054)	0.211 (0.051)	-0.236	0.060	-0.359	6.68E-06
cg20889322	-	0.255 (0.051)	0.256 (0.061)	0.258 (0.054)	0.066	0.628	-0.184	0.034
cg20146909	*LRRC8D*	0.669 (0.044)	0.668 (0.037)	0.679 (0.039)	-0.318	0.016	-0.303	2.90E-04
cg09419102	-	0.586 (0.053)	0.591 (0.056)	0.587 (0.057)	0.021	0.876	-0.149	0.077
cg15159987	*CPAMD8*	0.586 (0.051)	0.618 (0.051)	0.602 (0.055)	0.102	0.413	-0.305	1.21E-04
cg14785479	*SCARF2*	0.133 (0.054)	0.128 (0.048)	0.128 (0.055)	0.035	0.793	0.149	0.076
cg10420527	*LRP5*	0.471 (0.048)	0.462 (0.052)	0.487 (0.051)	-0.535	2.64E-05	-0.460	1.28E-08
cg06959340	*JUB*	0.061 (0.014)	0.062 (0.018)	0.065 (0.015)	-0.107	0.447	-0.246	0.006
cg14977938	*ZFYVE21*	0.641 (0.054)	0.615 (0.058)	0.623 (0.051)	0.011	0.936	0.405	1.56E-06
cg02583484	*HNRNPA1*	0.281 (0.055)	0.300 (0.061)	0.297 (0.058)	0.104	0.433	-0.308	2.49E-04
cg03129384	*FAM196A*	0.211 (0.077)	0.185 (0.079)	0.203 (0.087)	-0.109	0.380	0.153	0.052
cg20399616	*BCAT1*	0.065 (0.023)	0.061 (0.015)	0.060 (0.016)	0.151	0.281	0.414	3.37E-06
cg26963277	*KCNQ1OT1*	0.858 (0.038)	0.864 (0.035)	0.873 (0.032)	-0.254	0.062	-0.473	5.32E-08
cg23193870	*PTPN6*	0.078 (0.024)	0.080 (0.027)	0.085 (0.031)	-0.051	0.690	-0.394	1.22E-06
cg12876356	*GFI1*	0.760 (0.061)	0.763 (0.047)	0.772 (0.050)	-0.224	0.102	-0.355	4.34E-05
cg13711966	-	0.582 (0.063)	0.560 (0.077)	0.578 (0.075)	-0.241	0.071	0.111	0.186
cg03242819	*DOCK1*	0.155 (0.084)	0.117 (0.057)	0.125 (0.081)	-0.111	0.394	0.398	1.70E-06
cg18446336	*GNA12*	0.489 (0.136)	0.509 (0.127)	0.528 (0.130)	-0.189	0.127	-0.403	3.57E-07
cg05010058	*CEP68*	0.108 (0.024)	0.104 (0.022)	0.118 (0.028)	-0.410	1.31E-03	-0.410	4.21E-07
cg20664238	*NTRK3*	0.104 (0.035)	0.099 (0.035)	0.097 (0.033)	0.094	0.469	0.342	3.20E-05
cg15342087	-	0.752 (0.043)	0.755 (0.046)	0.768 (0.034)	-0.395	3.76E-03	-0.510	4.71E-09
cg21618017	*RILPL1*	0.078 (0.026)	0.075 (0.024)	0.084 (0.033)	-0.347	5.38E-03	-0.342	1.52E-05
cg17489908	*GATA3*	0.292 (0.062)	0.295 (0.075)	0.305 (0.071)	-0.281	0.030	-0.385	3.11E-06
cg13668129	*HNRNPUL1*	0.212 (0.066)	0.226 (0.077)	0.222 (0.067)	-0.091	0.485	-0.220	0.008
cg12836863	*BRCA2*	0.387 (0.138)	0.337 (0.131)	0.373 (0.129)	-0.379	2.96E-03	0.047	0.563
cg03603381	*RASGRP1*	0.453 (0.032)	0.454 (0.033)	0.467 (0.037)	-0.342	0.013	-0.354	5.11E-05
cg18630040	*PLA2G7*	0.068 (0.037)	0.059 (0.028)	0.061 (0.033)	-0.028	0.825	0.317	6.94E-05
cg01765406	-	0.423 (0.071)	0.395 (0.057)	0.427 (0.072)	-0.493	6.83E-05	-0.305	9.71E-05
cg13724496	*BMP2*	0.072 (0.028)	0.063 (0.031)	0.066 (0.025)	-0.048	0.708	0.300	2.26E-04
cg13990486	*FLJ43663*	0.099 (0.014)	0.102 (0.013)	0.104 (0.018)	-0.158	0.260	-0.358	5.94E-05
cg05270224	-	0.398 (0.074)	0.378 (0.097)	0.379 (0.084)	0.100	0.443	0.264	0.001
cg25310233	-	0.386 (0.075)	0.385 (0.072)	0.401 (0.074)	-0.204	0.099	-0.316	5.42E-05

cg24032269	*TCOF1*	0.665 (0.076)	0.671 (0.079)	0.644 (0.086)	0.319	4.44E-03	0.339	2.05E-06
cg13774342	-	0.778 (0.048)	0.802 (0.046)	0.788 (0.052)	0.241	0.079	-0.148	0.085
cg18150958	*RPL23A*	0.781 (0.040)	0.790 (0.047)	0.789 (0.049)	0.004	0.975	-0.200	0.022
cg18165852	*CHST13*	0.085 (0.034)	0.077 (0.024)	0.078 (0.030)	-0.084	0.525	0.321	1.33E-04
cg26242531	*ZFYVE21*	0.367 (0.088)	0.331 (0.070)	0.357 (0.088)	-0.152	0.199	0.177	0.018
cg12756150	-	0.760 (0.056)	0.749 (0.079)	0.738 (0.073)	0.151	0.234	0.432	9.37E-08
cg02150910	*GZMH*	0.724 (0.065)	0.734 (0.066)	0.707 (0.058)	0.266	0.024	0.340	6.33E-06
cg21473814	*CRTC1*	0.643 (0.062)	0.616 (0.067)	0.622 (0.066)	-0.093	0.485	0.326	1.22E-04
cg00619505	*TMCO3*	0.793 (0.045)	0.788 (0.060)	0.789 (0.050)	-0.010	0.937	0.296	2.15E-04
cg21188533	*CACNA1D*	0.587 (0.126)	0.541 (0.138)	0.557 (0.138)	-0.075	0.590	0.348	7.81E-05
cg13418576	-	0.404 (0.060)	0.408 (0.071)	0.422 (0.057)	-0.428	1.62E-03	-0.401	3.31E-06
cg17924476	*AHRR*	0.458 (0.115)	0.467 (0.123)	0.438 (0.111)	0.073	0.568	0.262	0.001
cg02532700	*NCF4*	0.181 (0.053)	0.183 (0.074)	0.197 (0.059)	-0.279	0.039	-0.417	1.32E-06
cg12593793	-	0.253 (0.070)	0.250 (0.059)	0.263 (0.070)	-0.291	0.019	-0.331	2.74E-05
cg14544289	*SPTLC2*	0.754 (0.045)	0.743 (0.045)	0.768 (0.049)	-0.616	5.38E-06	-0.488	1.36E-08
cg22619824	*ST7*	0.506 (0.073)	0.508 (0.081)	0.524 (0.078)	-0.260	0.042	-0.470	9.17E-09
cg22807449	*HOXB2*	0.355 (0.049)	0.340 (0.050)	0.357 (0.055)	-0.398	2.86E-03	-0.209	0.013
cg15417641	*CACNA1D*	0.621 (0.103)	0.587 (0.110)	0.593 (0.115)	-0.023	0.864	0.363	2.88E-05
cg07381806	*MOBKL2A*	0.419 (0.077)	0.423 (0.081)	0.437 (0.072)	-0.126	0.373	-0.348	1.04E-04
cg08126789	*USP10*	0.765 (0.044)	0.766 (0.050)	0.760 (0.044)	0.222	0.083	0.337	3.51E-05
cg03844971	*MLXIP*	0.113 (0.028)	0.117 (0.030)	0.119 (0.028)	-0.021	0.868	-0.229	0.004
cg05248618	*CA4*	0.063 (0.017)	0.063 (0.013)	0.058 (0.015)	0.422	2.16E-03	0.403	3.92E-06
cg05655806	*CD96*	0.432 (0.077)	0.447 (0.071)	0.448 (0.084)	-0.059	0.657	-0.232	0.005
cg09658497	*GNA12*	0.686 (0.124)	0.694 (0.129)	0.731 (0.113)	-0.150	0.268	-0.391	6.09E-06
cg06008724	*PHF21B*	0.175 (0.091)	0.166 (0.082)	0.156 (0.085)	0.086	0.492	0.388	1.14E-06
cg26348226	*ECE1*	0.221 (0.079)	0.208 (0.056)	0.234 (0.077)	-0.331	2.94E-03	-0.320	5.68E-06
cg18642234	*GPX1*	0.464 (0.055)	0.462 (0.046)	0.477 (0.049)	-0.297	0.021	-0.378	4.12E-06
cg19717773	*GNA12*	0.604 (0.119)	0.626 (0.118)	0.639 (0.114)	-0.125	0.359	-0.380	1.20E-05
cg11295113	*FOLR2*	0.784 (0.046)	0.782 (0.066)	0.775 (0.050)	0.190	0.158	0.273	0.001
cg01500140	*LIM2*	0.674 (0.089)	0.671 (0.092)	0.662 (0.093)	0.141	0.196	0.279	5.48E-05
cg15746583	*CD8B*	0.136 (0.060)	0.137 (0.065)	0.135 (0.061)	0.043	0.743	0.061	0.462
cg10416861	-	0.747 (0.043)	0.746 (0.050)	0.736 (0.049)	0.038	0.771	0.300	2.94E-04

cg09570614	-	0.430 (0.059)	0.400 (0.056)	0.416 (0.060)	-0.143	0.254	0.222	0.005
cg24687805	*RAB27A*	0.107 (0.030)	0.098 (0.022)	0.097 (0.028)	-0.092	0.482	0.317	1.31E-04
cg13481776	*ALPI*	0.713 (0.070)	0.721 (0.058)	0.693 (0.074)	0.301	8.09E-03	0.308	1.93E-05
cg09686308	*CIB3*	0.579 (0.068)	0.576 (0.065)	0.570 (0.066)	0.028	0.827	0.236	0.004
cg24996979	*C14orf43*	0.191 (0.043)	0.199 (0.052)	0.198 (0.055)	0.061	0.635	-0.313	1.25E-04
cg03646329	*LPAR6*	0.667 (0.074)	0.707 (0.067)	0.672 (0.091)	0.384	3.75E-03	-0.034	0.685
cg13314145	*NPTX2*	0.109 (0.045)	0.102 (0.030)	0.098 (0.034)	0.032	0.810	0.312	2.18E-04
cg19713429	*CAPZB*	0.241 (0.055)	0.250 (0.068)	0.253 (0.063)	0.166	0.205	-0.255	0.002
cg12303084	*ZMYND8*	0.179 (0.066)	0.181 (0.084)	0.186 (0.068)	0.028	0.828	-0.129	0.119
cg21752525	-	0.776 (0.047)	0.782 (0.048)	0.781 (0.043)	-0.026	0.846	-0.155	0.071
cg15451980	*RORA*	0.787 (0.063)	0.787 (0.066)	0.770 (0.069)	0.318	5.40E-03	0.305	2.64E-05
cg15187398	*MOBKL2A*	0.430 (0.079)	0.431 (0.083)	0.468 (0.070)	-0.615	3.14E-06	-0.600	1.13E-12
cg00326958	*HNRNPF*	0.120 (0.034)	0.137 (0.059)	0.122 (0.043)	0.276	0.041	0.006	0.940
cg24996482	-	0.072 (0.040)	0.064 (0.042)	0.066 (0.036)	0.124	0.352	0.303	3.35E-04
cg05007126	*SDCBP2*	0.710 (0.046)	0.728 (0.039)	0.721 (0.043)	0.074	0.569	-0.214	0.009
cg26958735	-	0.087 (0.021)	0.085 (0.021)	0.083 (0.020)	0.122	0.361	0.241	0.004
cg03172931	-	0.694 (0.046)	0.688 (0.047)	0.678 (0.055)	0.318	0.011	0.442	2.68E-08
cg15554421	*C3orf26*	0.186 (0.047)	0.179 (0.055)	0.192 (0.053)	-0.302	0.012	-0.234	0.002
cg11485823	*DPCR1*	0.693 (0.053)	0.682 (0.063)	0.683 (0.055)	0.024	0.856	0.243	0.004
cg24741609	*GLIS1*	0.338 (0.059)	0.358 (0.075)	0.357 (0.067)	-0.072	0.593	-0.361	2.37E-05
cg10179300	*TRIO*	0.783 (0.042)	0.788 (0.041)	0.794 (0.036)	-0.013	0.925	-0.338	1.35E-04
cg02352716	-	0.706 (0.081)	0.712 (0.065)	0.689 (0.080)	0.156	0.158	0.249	3.76E-04
cg09747445	*TLE3*	0.552 (0.089)	0.556 (0.072)	0.572 (0.076)	-0.202	0.106	-0.345	1.39E-05
cg02869235	-	0.469 (0.088)	0.455 (0.066)	0.467 (0.087)	-0.134	0.298	0.070	0.390
cg22574825	*FLT1*	0.094 (0.042)	0.083 (0.038)	0.080 (0.034)	0.171	0.200	0.464	4.60E-08
cg13185177	*GP5*	0.497 (0.101)	0.460 (0.093)	0.472 (0.097)	0.014	0.906	0.337	1.04E-05
cg22851200	*TRIP6*	0.573 (0.067)	0.561 (0.086)	0.591 (0.072)	-0.392	2.93E-03	-0.333	6.79E-05
cg15207742	*RIMS4*	0.092 (0.043)	0.081 (0.033)	0.080 (0.041)	-0.117	0.367	0.357	1.50E-05
cg14074174	*SNAPC2*	0.360 (0.047)	0.354 (0.047)	0.369 (0.041)	-0.291	0.016	-0.400	1.93E-07
cg16611234	-	0.326 (0.075)	0.332 (0.093)	0.343 (0.070)	-0.291	0.034	-0.439	4.91E-07
cg26361535	*ZC3H3*	0.653 (0.076)	0.659 (0.088)	0.680 (0.062)	-0.520	1.27E-04	-0.455	1.32E-07
cg23867146	-	0.594 (0.033)	0.581 (0.039)	0.588 (0.046)	-0.063	0.625	0.095	0.240

cg11621113	*MORG1*	0.314 (0.085)	0.327 (0.097)	0.331 (0.094)	-0.011	0.934	-0.265	0.001
cg07069636	-	0.430 (0.071)	0.439 (0.074)	0.436 (0.069)	0.121	0.312	-0.174	0.021
cg12884422	-	0.291 (0.117)	0.317 (0.101)	0.303 (0.100)	-0.024	0.867	-0.275	0.002
cg07986378	*ETV6*	0.537 (0.087)	0.543 (0.078)	0.563 (0.089)	-0.259	0.044	-0.411	4.46E-07
cg26364091	*CHADL*	0.635 (0.026)	0.636 (0.030)	0.647 (0.027)	-0.317	0.018	-0.546	2.09E-10
cg12158535	*PACS2*	0.627 (0.038)	0.611 (0.030)	0.615 (0.037)	0.089	0.518	0.312	3.75E-04
cg21322436	*CNTNAP2*	0.272 (0.052)	0.287 (0.058)	0.284 (0.047)	0.010	0.940	-0.277	0.001
cg10126923	*NKG7*	0.294 (0.084)	0.269 (0.075)	0.285 (0.075)	-0.181	0.160	0.160	0.050
cg10951873	*RUNX3*	0.109 (0.027)	0.120 (0.036)	0.111 (0.032)	0.278	0.036	-0.115	0.166
cg05284742	*ITPK1*	0.655 (0.049)	0.658 (0.051)	0.669 (0.052)	-0.387	2.57E-03	-0.481	4.44E-09
cg12873476	-	0.611 (0.064)	0.616 (0.068)	0.641 (0.067)	-0.281	0.027	-0.537	4.33E-11
cg21733502	*ZSCAN5B*	0.824 (0.035)	0.828 (0.043)	0.827 (0.032)	-0.054	0.695	-0.193	0.026
cg01294327	*LINGO3*	0.621 (0.109)	0.612 (0.146)	0.629 (0.106)	-0.221	0.114	-0.200	0.024
cg15380836	*RILP*	0.132 (0.062)	0.137 (0.065)	0.144 (0.067)	-0.138	0.268	-0.240	0.002
cg06419750	*GLT1D1*	0.138 (0.071)	0.121 (0.054)	0.127 (0.064)	-0.003	0.984	0.266	0.001
cg00336149	*CACNA1D*	0.367 (0.078)	0.334 (0.081)	0.346 (0.084)	-0.071	0.607	0.315	3.21E-04
cg22777952	*FOXB1*	0.089 (0.019)	0.085 (0.016)	0.086 (0.024)	-0.242	0.070	0.166	0.048
cg14667406	*LCT*	0.841 (0.028)	0.843 (0.029)	0.848 (0.029)	-0.038	0.781	-0.283	0.001
cg10204884	*PCBP3*	0.742 (0.043)	0.751 (0.041)	0.747 (0.037)	0.103	0.426	-0.128	0.119
cg12916723	*NKG7*	0.606 (0.045)	0.603 (0.045)	0.591 (0.047)	0.083	0.501	0.412	1.56E-07
cg01726890	-	0.699 (0.049)	0.703 (0.057)	0.685 (0.062)	0.163	0.162	0.372	6.02E-07
cg03877174	*KIF1C*	0.223 (0.069)	0.218 (0.064)	0.215 (0.062)	0.001	0.995	0.190	0.022
cg05635807	-	0.063 (0.023)	0.056 (0.017)	0.054 (0.017)	0.199	0.153	0.459	2.44E-07
cg19956914	*SUMF2*	0.473 (0.092)	0.427 (0.086)	0.453 (0.069)	-0.208	0.124	0.297	5.25E-04
cg10908953	*SORL1*	0.190 (0.069)	0.190 (0.062)	0.199 (0.070)	-0.145	0.253	-0.231	0.004
cg14096889	*ADAMTSL3*	0.101 (0.040)	0.094 (0.046)	0.095 (0.033)	0.166	0.200	0.393	1.91E-06
cg23351584	*PRSS23*	0.140 (0.032)	0.136 (0.032)	0.148 (0.031)	-0.399	1.95E-03	-0.438	8.84E-08
cg13389508	*PLEC1*	0.424 (0.039)	0.437 (0.031)	0.431 (0.044)	0.129	0.322	-0.257	0.002
cg09762515	*CUX1*	0.605 (0.081)	0.592 (0.078)	0.599 (0.08)	-0.074	0.569	0.152	0.063
cg16145216	*HIVEP3*	0.364 (0.080)	0.365 (0.074)	0.326 (0.082)	0.472	3.75E-04	0.626	2.25E-13
cg04039397	*CD96*	0.296 (0.065)	0.315 (0.074)	0.314 (0.061)	0.020	0.881	-0.372	1.71E-05
cg02818189	-	0.550 (0.093)	0.513 (0.085)	0.569 (0.093)	-0.455	1.48E-04	-0.365	1.51E-06

cg09301294	*EPHA6*	0.111 (0.029)	0.102 (0.036)	0.107 (0.032)	-0.067	0.594	0.222	0.005
cg25260137	-	0.124 (0.051)	0.115 (0.056)	0.114 (0.047)	0.023	0.859	0.312	1.53E-04
cg05228408	*CLCN6*	0.359 (0.057)	0.355 (0.056)	0.367 (0.054)	-0.224	0.065	-0.317	4.14E-05
cg02985540	-	0.278 (0.066)	0.271 (0.074)	0.287 (0.067)	-0.217	0.083	-0.249	0.002
cg26971042	*TLE3*	0.214 (0.039)	0.215 (0.040)	0.223 (0.045)	-0.103	0.410	-0.379	1.75E-06
cg06868100	*PRR15*	0.239 (0.080)	0.257 (0.077)	0.246 (0.100)	0.043	0.759	-0.113	0.202
cg19827923	*GPR55*	0.747 (0.045)	0.753 (0.041)	0.753 (0.044)	0.112	0.406	-0.155	0.069
cg14580211	*C5orf62*	0.602 (0.087)	0.598 (0.079)	0.622 (0.068)	-0.414	5.75E-04	-0.434	1.45E-08
cg07827420	*SEPT9*	0.435 (0.030)	0.438 (0.040)	0.443 (0.032)	-0.105	0.455	-0.340	1.38E-04
cg01107178	*ANKRD11*	0.647 (0.072)	0.661 (0.076)	0.631 (0.077)	0.392	5.32E-04	0.286	6.48E-05
cg14588779	*AKAP8L*	0.170 (0.053)	0.170 (0.057)	0.174 (0.057)	-0.086	0.519	-0.165	0.050
cg05460226	*PIK3R5*	0.390 (0.101)	0.401 (0.114)	0.432 (0.115)	-0.403	1.59E-03	-0.481	3.52E-09
cg14428590	*FSIP1*	0.404 (0.066)	0.393 (0.052)	0.415 (0.062)	-0.198	0.119	-0.381	2.29E-06
cg26736540	*TFAP2C*	0.068 (0.023)	0.064 (0.016)	0.065 (0.020)	-0.017	0.900	0.265	0.002
cg01564343	*TREML1*	0.670 (0.046)	0.665 (0.041)	0.657 (0.045)	0.238	0.069	0.353	2.14E-05
cg13193840	-	0.144 (0.033)	0.148 (0.030)	0.154 (0.036)	-0.204	0.144	-0.357	5.63E-05
cg13039251	*PDZD2*	0.727 (0.068)	0.687 (0.089)	0.704 (0.076)	-0.182	0.139	0.195	0.012
cg06901890	*FNBP1*	0.187 (0.039)	0.200 (0.055)	0.195 (0.046)	0.122	0.368	-0.158	0.065
cg17287155	*AHRR*	0.831 (0.032)	0.841 (0.038)	0.838 (0.038)	0.117	0.379	-0.240	0.005
cg20303561	*CCDC88C*	0.652 (0.063)	0.666 (0.083)	0.671 (0.056)	-0.039	0.765	-0.241	0.004
cg03760919	*RUNX1T1*	0.701 (0.053)	0.706 (0.069)	0.691 (0.055)	0.374	2.68E-03	0.215	0.006
cg04755561	*PKMYT1*	0.655 (0.057)	0.674 (0.059)	0.659 (0.047)	0.252	0.032	-0.161	0.030
cg04105282	*CRTAC1*	0.115 (0.051)	0.109 (0.040)	0.101 (0.043)	0.208	0.114	0.417	6.92E-07
cg08548559	*PIK3IP1*	0.287 (0.084)	0.296 (0.113)	0.310 (0.095)	0.030	0.820	-0.285	6.17E-04
cg16556677	*KCNQ1OT1*	0.734 (0.044)	0.739 (0.053)	0.746 (0.056)	-0.319	0.017	-0.337	7.38E-05
cg06009448	*C7orf50*	0.365 (0.058)	0.364 (0.060)	0.379 (0.050)	-0.311	0.011	-0.343	9.08E-06
cg19784816	*ITIH1*	0.697 (0.061)	0.694 (0.060)	0.683 (0.065)	0.165	0.205	0.309	1.86E-04
cg00378510	*LINGO3*	0.447 (0.105)	0.457 (0.094)	0.459 (0.112)	-0.065	0.627	-0.123	0.150
cg08709672	*AVPR1B*	0.569 (0.047)	0.572 (0.046)	0.595 (0.048)	-0.446	8.18E-04	-0.642	7.37E-14
cg19838043	*ZFYVE21*	0.611 (0.068)	0.581 (0.077)	0.596 (0.072)	-0.093	0.477	0.215	0.009
cg07123182	*KCNQ1OT1*	0.907 (0.026)	0.908 (0.020)	0.915 (0.019)	-0.218	0.112	-0.412	2.49E-06
cg18387156	*NRG1*	0.155 (0.054)	0.152 (0.053)	0.149 (0.055)	0.005	0.969	0.147	0.082

cg01416295	*MRPL24*	0.314 (0.069)	0.312 (0.073)	0.318 (0.088)	-0.086	0.525	-0.179	0.036
cg01598741	*HMGA2*	0.695 (0.070)	0.692 (0.072)	0.704 (0.069)	-0.099	0.485	-0.115	0.195
cg20185017	*CACNB2*	0.084 (0.029)	0.082 (0.021)	0.078 (0.026)	0.184	0.167	0.332	8.39E-05
cg25922751	*NCOR2*	0.067 (0.017)	0.073 (0.020)	0.069 (0.015)	0.273	0.026	-0.154	0.047
cg07066369	*CCND2*	0.067 (0.012)	0.064 (0.015)	0.070 (0.015)	-0.436	1.70E-03	-0.356	5.29E-05

[a] Estimates were calculated by linear regression models adjusting for age, drinking status, BMI, disease status, geographical regions, differential leukocyte proportions and beadchip operation date.

Table S4. Methylation-gene expression correlations of the smoking-related CpGs in SY (n = 144).

CpG information					Transcripts information		CpG-expression probe association		
**Chr**	**Position**	**CpG**	**Gene**	**Relation to Gene**	**Probe**	**Gene**	**Effect**	**s.e.**	***p***
1	11865352	cg05228408	*MTHFR*	TSS1500	ILMN_1734830	*MTHFR*	7.733	2.148	4.41E-04
1	11865352	cg05228408	*CLCN6*	TSS1500	ILMN_1763036	*CLCN6*	7.137	2.156	0.001
1	16301562	cg04211179	*ZBTB17*	5'UTR	ILMN_1711048	*ZBTB17*	7.856	1.711	9.73E-06
1	21617442	cg26348226	*ECE1*	TSS1500	ILMN_1672174	*ECE1*	-14.337	1.926	9.11E-12
1	25254746	cg10951873	*RUNX3*	Body	ILMN_1787461	*RUNX3*	-26.344	4.769	1.57E-07
1	28521540	cg20460771	*PTAFR*	TSS1500	ILMN_1746836	*PTAFR*	13.025	1.395	2.07E-16
1	90289611	cg20146909	*LRRC8D*	5'UTR	ILMN_1763409	*LRRC8D*	6.287	2.812	0.027
1	153746211	cg08129092	*INTS3*	3'UTR	ILMN_1756086	*INTS3*	-16.351	2.352	1.27E-10
1	154299179	cg06811467	*ATP8B2*	TSS1500	ILMN_1782057	*ATP8B2*	11.790	2.625	1.47E-05
1	154379696	cg09257526	*IL6R*	Body	ILMN_1696394	*IL6R*	-16.010	2.494	1.98E-09
1	154379696	cg09257526	*IL6R*	Body	ILMN_1754753	*IL6R*	-12.405	2.627	5.61E-06
2	231790037	cg16382047	*GPR55*	TSS200	ILMN_1723274	*GPR55*	7.689	1.974	1.52E-04
2	231790777	cg19827923	*GPR55*	TSS1500	ILMN_1723274	*GPR55*	-17.588	3.253	2.70E-07
3	49394622	cg18642234	*GPX1*	3'UTR	ILMN_1749662	*GPX1*	4.120	1.963	0.038
3	111260756	cg05655806	*CD96*	TSS200	ILMN_2415786	*CD96*	-7.176	1.715	5.05E-05
3	111260756	cg05655806	*CD96*	TSS200	ILMN_1711573	*CD96*	-9.435	1.646	5.89E-08
3	111260783	cg04039397	*CD96*	TSS200	ILMN_2415786	*CD96*	-6.825	1.525	1.57E-05
3	111260783	cg04039397	*CD96*	TSS200	ILMN_1711573	*CD96*	-9.026	1.449	5.36E-09
3	126242973	cg18165852	*CHST13*	TSS1500	ILMN_1734707	*CHST13*	-8.940	2.845	0.002
3	188425256	cg25853622	*LPP*	Body	ILMN_1651254	*LPP*	-5.155	1.120	9.25E-06
3	194119885	cg13185177	*GP5*	5'UTR	ILMN_1780622	*GP5*	3.062	1.533	0.048
4	56813860	cg26542660	*CEP135*	TSS1500	ILMN_1693766	*CEP135*	-7.353	3.078	0.018
5	138861241	cg04232128	*TMEM173*	Body	ILMN_2145116	*TMEM173*	17.222	1.732	5.84E-18
6	35696870	cg25114611	*FKBP5*	TSS1500	ILMN_1778444	*FKBP5*	-6.479	2.793	0.022

6	36650733	cg01955533	*CDKN1A*	5'UTR	ILMN_1784602	*CDKN1A*	5.044	1.837	0.007
7	2847575	cg18446336	*GNA12*	Body	ILMN_2216157	*GNA12*	3.496	1.181	0.004
7	5457225	cg09022230	*TNRC18*	Body	ILMN_1678329	*TNRC18*	-9.621	1.664	4.61E-08
7	45002736	cg04180046	*MYO1G*	Body	ILMN_1692295	*MYO1G*	5.040	1.758	0.005
7	51384528	cg09613161	*COBL*	TSS200	ILMN_1711888	*COBL*	-2.977	0.990	0.003
7	51384609	cg04016086	*COBL*	TSS200	ILMN_1711888	*COBL*	-3.274	1.548	0.036
7	56147257	cg19956914	*SUMF2*	Body	ILMN_1685371	*SUMF2*	2.725	1.084	0.013
7	100465833	cg22851200	*TRIP6*	Body	ILMN_1713990	*TRIP6*	-5.725	1.636	6.24E-04
7	116786606	cg22619824	*ST7*	Body	ILMN_1702175	*ST7*	7.387	1.314	9.94E-08
7	116786606	cg22619824	*ST7*	Body	ILMN_1707763	*ST7*	5.346	1.376	1.57E-04
8	31496644	cg18387156	*NRG1*	TSS1500	ILMN_1737252	*NRG1*	6.519	2.535	0.011
8	126446923	cg22644321	*TRIB1*	Body	ILMN_1803811	*TRIB1*	-18.246	2.262	2.93E-13
8	144576604	cg26361535	*ZC3H3*	Body	ILMN_1702389	*ZC3H3*	-3.217	1.597	0.046
8	145012748	cg25325005	*PLEC1*	Body	ILMN_1744268	*PLEC1*	-5.925	1.567	2.30E-04
8	145012748	cg25325005	*PLEC1*	Body	ILMN_1736792	*PLEC1*	7.896	1.510	6.06E-07
10	49892930	cg15164194	*WDFY4*	TSS1500	ILMN_3236551	*WDFY4*	-11.274	1.524	1.17E-11
10	73497514	cg10750182	*CDH23*	Body	ILMN_1779934	*CDH23*	-4.980	1.893	0.009
11	2722391	cg07123182	*KCNQ1*	Body	ILMN_2409520	*KCNQ1*	9.891	3.372	0.004
11	2722407	cg26963277	*KCNQ1OT1*	TSS1500	ILMN_3248707	*KCNQ1OT1*	5.587	2.563	0.031
11	86510915	cg11660018	*PRSS23*	TSS1500	ILMN_1797776	*PRSS23*	7.850	1.692	7.94E-06
11	86510998	cg23771366	*PRSS23*	TSS1500	ILMN_1797776	*PRSS23*	8.629	2.068	5.25E-05
11	86512100	cg23351584	*PRSS23*	5'UTR	ILMN_1797776	*PRSS23*	20.719	4.950	5.00E-05
11	121322456	cg10908953	*SORL1*	TSS1500	ILMN_1759818	*SORL1*	-18.779	2.766	2.95E-10
11	121322456	cg10908953	*SORL1*	TSS1500	ILMN_2060115	*SORL1*	-21.510	2.635	1.70E-13
12	4384888	cg07066369	*CCND2*	Body	ILMN_2067656	*CCND2*	28.294	6.613	3.47E-05
12	4384888	cg07066369	*CCND2*	Body	ILMN_1667081	*CCND2*	24.758	6.752	3.48E-04
12	7055657	cg23193870	*PTPN6*	TSS200	ILMN_1738675	*PTPN6*	-23.094	6.997	0.001
12	7055657	cg23193870	*PTPN6*	TSS200	ILMN_1716578	*PTPN6*	-21.788	7.095	0.003

12	11898284	cg07986378	*ETV6*	Body	ILMN_1789596	*ETV6*	-6.905	1.419	3.04E-06
12	124016861	cg21618017	*RILPL1*	Body	ILMN_1805643	*RILPL1*	-23.846	7.746	0.003
12	129337910	cg06419750	*GLT1D1*	TSS200	ILMN_1656327	*GLT1D1*	-4.899	1.281	1.96E-04
13	48987165	cg03646329	*RB1*	5'UTR	ILMN_1696591	*RB1*	3.697	1.372	0.008
13	114172890	cg00619505	*TMCO3*	Body	ILMN_2220739	*TMCO3*	11.309	2.788	8.24E-05
14	25079357	cg02150910	*GZMH*	TSS1500	ILMN_1731233	*GZMH*	-14.102	1.462	3.38E-17
14	91818668	cg01055824	*CCDC88C*	Body	ILMN_3248352	*CCDC88C*	-14.824	3.736	1.15E-04
14	91881497	cg20303561	*CCDC88C*	Body	ILMN_3248352	*CCDC88C*	-10.949	1.963	1.21E-07
14	93552128	cg05284742	*ITPK1*	Body	ILMN_1715674	*ITPK1*	-9.874	2.247	2.18E-05
14	104190678	cg26242531	*ZFYVE21*	Body	ILMN_2197030	*ZFYVE21*	-5.361	1.477	3.97E-04
15	38857474	cg03603381	*RASGRP1*	TSS1500	ILMN_1768958	*RASGRP1*	5.513	2.317	0.019
15	55513621	cg24687805	*RAB27A*	Body	ILMN_1665859	*RAB27A*	-20.736	4.910	4.31E-05
15	55513621	cg24687805	*RAB27A*	Body	ILMN_1699878	*RAB27A*	-27.973	4.637	1.36E-08
15	55513621	cg24687805	*RAB27A*	Body	ILMN_2329773	*RAB27A*	-25.988	4.564	7.01E-08
15	60959637	cg15451980	*RORA*	Body	ILMN_2322499	*RORA*	-10.094	1.355	8.70E-12
15	60959637	cg15451980	*RORA*	Body	ILMN_2322498	*RORA*	-10.437	1.349	1.81E-12
15	60959637	cg15451980	*RORA*	Body	ILMN_1813134	*RORA*	-8.499	1.430	2.12E-08
15	70387217	cg26971042	*TLE3*	Body	ILMN_2234412	*TLE3*	-8.926	2.702	0.001
15	74862662	cg02384859	*ARID3B*	Body	ILMN_1696956	*ARID3B*	9.939	1.669	2.01E-08
15	90357202	cg23161492	*ANPEP*	5'UTR	ILMN_1763837	*ANPEP*	-5.939	2.097	0.005
16	17562960	cg06321596	*XYLT1*	Body	ILMN_1830462	*XYLT1*	8.401	2.067	7.97E-05
16	84746995	cg08126789	*USP10*	Body	ILMN_1721116	*USP10*	6.098	2.780	0.030
16	89041793	cg04887172	*CBFA2T3*	Body	ILMN_1657627	*CBFA2T3*	-6.106	1.268	3.78E-06
16	89408248	cg01107178	*ANKRD11*	5'UTR	ILMN_2108709	*ANKRD11*	8.837	1.451	1.03E-08
17	8804279	cg05460226	*PIK3R5*	Body	ILMN_1681067	*PIK3R5*	-6.339	1.955	0.001
17	9921982	cg02018337	*GAS7*	Body	ILMN_1745994	*GAS7*	-3.668	1.795	0.043
17	27050723	cg18150958	*RPL23A*	Body	ILMN_1788607	*RPL23A*	-7.159	1.797	1.09E-04
17	27401793	cg18960216	*TIAF1*	5'UTR	ILMN_2055634	*TIAF1*	-8.108	2.458	0.001

17	46622522	cg22807449	*HOXB2*	TSS200	ILMN_1810274	*HOXB2*	8.546	1.931	1.92E-05
17	56082867	cg08591265	*SFRS1*	3'UTR;Body	ILMN_1795341	*SFRS1*	-4.897	1.302	2.47E-04
17	58227267	cg05248618	*CA4*	TSS200	ILMN_1695157	*CA4*	-11.653	2.872	8.22E-05
17	75371764	cg07827420	*SEPT9*	5'UTR	ILMN_1769118	*SEPT9*	-8.857	3.586	0.015
17	75445905	cg07324245	*SEPT9*	Body	ILMN_1769118	*SEPT9*	-8.944	1.384	1.58E-09
19	51875451	cg10126923	*NKG7*	Body	ILMN_1682993	*NKG7*	9.290	1.462	2.75E-09
19	51876788	cg12916723	*NKG7*	TSS1500	ILMN_1682993	*NKG7*	-12.113	2.207	1.84E-07
20	1310884	cg05007126	*SDCBP2*	TSS1500	ILMN_1705107	*SDCBP2*	7.264	2.590	0.006
21	40180000	cg15892280	*ETS2*	5'UTR	ILMN_1720158	*ETS2*	-13.054	1.607	2.15E-13
22	31686097	cg08548559	*PIK3IP1*	Body	ILMN_1719986	*PIK3IP1*	-4.952	1.593	0.002
22	37257404	cg02532700	*NCF4*	Body	ILMN_2335704	*NCF4*	-8.136	2.809	0.004
22	37257404	cg02532700	*NCF4*	Body	ILMN_1757361	*NCF4*	-6.751	2.766	0.016
22	37257404	cg02532700	*NCF4*	Body	ILMN_1785005	*NCF4*	-8.782	2.835	0.002

Abbreviations: Chr, Chromosome; Body, gene body; TSS200, within 200 bps from transcription start site; TSS1500, within 1500 bps from transcription start site; UTR, untranslated regions.

For each CpG, the significance threshold was defined as 0.05/number of expression probes of the corresponding gene.

Table S5. Contribution rates of smoking on urinary PAHs metabolites calculated in male individuals from the WHZH cohort (n = 206).

OH-PAHs	Concentrations of OH-PAHs ($\times 10^{-2}$)	Model 1[a]		Model 2[b]		Variance of OH-PAHs explained by smoking[c] ΔR^2 (%)
		R^2	p	R^2	p	
1-hydroxynaphthalene	0.32 (0.22, 0.52)	0.152	9.30E-04	0.059	0.277	9.307
2-hydroxynaphthalene	0.58 (0.38, 1.00)	0.258	4.72E-08	0.079	0.095	17.979
2-hydroxyfluorene	0.12 (0.08, 0.17)	0.080	0.131	0.065	0.204	1.497
9-hydroxyfluorene	0.27 (0.18, 0.44)	0.070	0.214	0.064	0.205	0.548
1-hydroxyphenanthrene	0.08 (0.06, 0.14)	0.198	1.71E-05	0.198	7.46E-06	0.020
2-hydroxyphenanthrene	0.06 (0.04, 0.08)	0.119	0.012	0.117	0.007	0.133
3-hydroxyphenanthrene	0.11 (0,07, 0.14)	0.252	1.07E-07	0.250	4.74E-08	0.170
4-hydroxyphenanthrene	0.10 (0.07, 0.15)	0.126	0.007	0.121	0.006	0.500
9-hydroxyphenanthrene	0.22 (0.15, 0.30)	0.093	0.064	0.093	0.041	0.000
1-hydroxypyrene	0.24 (0.17, 0.36)	0.102	0.034	0.096	0.031	0.591
ΣOH-PAHs	2.41 (1.82, 3.09)	0.169	2.34E-04	0.122	0.005	4.608

The OH-PAHs levels were calibrated by urinary creatinine and presented as micromoles per millimole creatinine. Concentrations of OH-PAHs ($\times 10^{-2}$) are median (25th, 75th).

[a] Model 1: Linear regression models with urinary metabolite as dependent variables, age, drinking, BMI, occupation, geographical regions beadchip and operation date included as independent variables. Concentrations of urinary PAHs metabolites were ln-transformed. [b] Model 2: additionally included smoking in Model 1.[c]The variance of each urinary OH-PAH metabolite explained by smoking, calculated as the variance explained by Model 2 minus the variance explained by Model 1.

Table S6. Associations between the smoking-related CpGs and urinary 2-hydroxynaphthalene levels in males from the WHZH Cohort and the Coke Oven Cohort[a].

CpG	Gene	WHZH (n = 206)		WHZH nonsmoker (n = 72)		COW (n = 107)		COW nonsmoker (n = 20)	
		Effect[b]	p	Effect[b]	p	Effect[c]	p	Effect[c]	p
cg05575921	*AHRR*	-0.664	1.94E-09	-0.223	0.284	-0.679	1.40E-05	-0.062	0.907
cg23576855	*AHRR*	-0.581	1.01E-07	-0.161	0.477	-0.587	1.57E-04	0.130	0.790
cg21566642	-	-0.574	2.59E-07	0.065	0.766	-0.531	4.88E-04	-0.413	0.311
cg05951221	-	-0.435	7.14E-05	-0.047	0.833	-0.573	3.39E-04	-0.719	0.077
cg26703534	*AHRR*	-0.529	6.58E-07	-0.463	0.028	-0.319	0.020	0.055	0.884
cg03636183	*F2RL3*	-0.462	2.70E-05	-0.252	0.226	-0.311	0.056	0.327	0.563
cg01940273	-	-0.592	2.88E-08	-0.248	0.250	-0.470	0.002	0.264	0.613
cg25648203	*AHRR*	-0.475	1.26E-05	-0.545	0.015	-0.377	0.017	0.346	0.348
cg22132788	*MYO1G*	0.334	0.004	0.138	0.599	0.426	0.006	0.266	0.673
cg21161138	*AHRR*	-0.562	3.78E-07	-0.321	0.180	-0.503	0.002	-0.715	0.201
cg03329539	-	-0.407	1.64E-04	0.095	0.667	-0.394	0.014	-0.234	0.621
cg06126421	-	-0.393	1.61E-04	0.172	0.423	-0.346	0.010	-0.026	0.944
cg01513913	-	-0.328	0.003	-0.176	0.482	-0.355	0.021	0.388	0.372
cg12803068	*MYO1G*	0.242	0.039	0.255	0.296	0.429	0.007	-0.125	0.832
cg21611682	*LRP5*	-0.362	0.001	-0.473	0.052	-0.373	0.024	0.571	0.206
cg00501876	*CSRNP1*	-0.302	0.007	-0.113	0.654	-0.133	0.382	-0.414	0.370
cg14817490	*AHRR*	-0.378	6.26E-04	-0.165	0.485	-0.450	0.006	-1.025	0.013
cg01208318	-	-0.331	0.003	0.002	0.994	-0.479	0.003	0.224	0.630
cg06811467	*ATP8B2*	-0.112	0.269	0.160	0.462	-0.186	0.202	-0.249	0.576
cg24540678	-	-0.210	0.061	0.050	0.825	-0.393	0.017	-0.334	0.524
cg23594345	-	-0.338	0.003	-0.228	0.349	-0.353	0.034	0.520	0.274
cg02018337	*GAS7*	-0.103	0.302	-0.087	0.697	-0.301	0.015	-0.246	0.511

cg07573717	*CAPZB*	-0.071	0.493	-0.228	0.272	-0.288	0.041	-0.569	0.207
cg14387626	-	-0.182	0.088	-0.246	0.263	-0.212	0.141	0.265	0.556
cg23161492	*ANPEP*	-0.417	2.87E-04	-0.112	0.637	-0.297	0.069	0.453	0.347
cg03991871	*AHRR*	-0.320	0.004	-0.300	0.206	-0.377	0.018	-8.351	0.999
cg00980649	-	-0.095	0.402	0.092	0.703	-0.382	0.017	0.396	0.388
cg11660018	*PRSS23*	-0.364	3.77E-04	-0.405	0.054	-0.382	0.005	0.362	0.440
cg03440944	*C7orf40*	-0.002	0.989	0.503	0.027	-0.286	0.019	-0.224	0.501
cg19089201	*MYO1G*	0.292	0.014	0.508	0.046	0.295	0.062	0.254	0.654
cg23079012	-	-0.354	0.001	-0.506	0.014	-0.097	0.556	0.711	0.109
cg13074055	-	-0.153	0.172	0.207	0.363	-0.318	0.060	0.876	0.064
cg26542660	*CEP135*	-0.073	0.461	0.122	0.575	-0.136	0.343	-0.324	0.449
cg22512531	*CRTAM*	0.190	0.076	0.290	0.187	0.212	0.073	-0.248	0.379
cg15164194	*WDFY4*	-0.144	0.163	-0.153	0.506	-0.145	0.301	-0.088	0.845
cg27113548	-	-0.281	0.014	-0.293	0.225	-0.361	0.017	0.453	0.391
cg19572487	*RARA*	-0.332	0.004	0.357	0.122	-0.306	0.034	-0.195	0.610
cg19859270	*GPR15*	-0.350	0.002	-0.141	0.547	-0.605	1.77E-04	-0.614	0.150
cg18960216	*TIAF1*	0.106	0.291	0.102	0.644	0.359	0.011	0.601	0.238
cg07978738	*ABLIM1*	-0.112	0.285	0.066	0.755	-0.349	0.023	-0.155	0.766
cg25189904	*GNG12*	-0.374	9.39E-04	0.364	0.132	-0.342	0.037	-0.214	0.581
cg04232128	*TMEM173*	-0.060	0.549	0.186	0.416	-0.270	0.056	-0.170	0.694
cg23916896	*AHRR*	-0.458	5.18E-05	-0.594	0.012	-0.100	0.536	-0.018	0.962
cg07339236	*ATP9A*	-0.291	0.007	-0.263	0.221	-0.178	0.265	0.379	0.517
cg18946533	*SH2D7*	-0.048	0.679	-0.168	0.475	0.408	0.014	0.625	0.201
cg05080154	*SALL3*	0.167	0.111	-0.279	0.197	0.375	0.016	0.387	0.175
cg12806681	*AHRR*	-0.247	0.026	0.078	0.737	-0.414	0.011	-0.312	0.477
cg09088988	*STK32A*	-0.090	0.407	-0.244	0.255	0.118	0.473	0.226	0.690
cg26850624	*AHRR*	0.135	0.202	0.285	0.172	0.186	0.221	0.753	0.187
cg23771366	*PRSS23*	-0.310	0.004	-0.277	0.195	-0.428	0.005	0.005	0.993

cg04016086	*COBL*	0.050	0.664	-0.388	0.112	0.320	0.035	0.479	0.257
cg10750182	*CDH23*	-0.309	0.004	-0.541	0.014	-0.254	0.068	-0.170	0.617
cg01692968	-	-0.363	7.14E-04	-0.210	0.286	-0.224	0.106	0.490	0.197
cg09578155	*LRP5*	-0.148	0.166	0.004	0.986	-0.098	0.515	0.011	0.984
cg23186333	*CD44*	-0.002	0.987	0.074	0.732	-0.199	0.183	0.060	0.917
cg09084200	*VPS26B*	-0.107	0.329	0.153	0.484	-0.242	0.134	-0.119	0.802
cg25305703	-	-0.159	0.143	0.142	0.499	-0.131	0.401	-0.047	0.927
cg14556677	*NEK6*	-0.103	0.307	-0.044	0.843	-0.149	0.315	-0.448	0.358
cg03217253	*ZNF677*	-0.028	0.810	-0.421	0.065	0.232	0.138	0.530	0.177
cg14663208	*HIVEP3*	0.198	0.070	0.078	0.754	0.272	0.034	0.147	0.588
cg14753356	-	-0.303	0.003	0.008	0.968	-0.375	0.003	-0.538	0.141
cg09935388	*GFI1*	-0.425	8.82E-05	0.109	0.627	-0.279	0.095	0.312	0.551
cg08595501	*IQGAP2*	-0.299	0.011	-0.134	0.577	-0.214	0.199	0.660	0.238
cg07465627	*STXBP4*	-0.100	0.369	0.269	0.271	-0.197	0.211	-0.492	0.197
cg15693483	*C7orf50*	-0.308	0.002	-0.501	0.031	-0.143	0.284	0.716	0.059
cg22644321	*TRIB1*	-0.219	0.028	-0.227	0.285	-0.153	0.270	-0.539	0.112
cg27537125	-	-0.118	0.285	0.288	0.199	-0.177	0.236	-0.410	0.311
cg19708306	-	0.072	0.485	-0.020	0.930	0.178	0.171	0.274	0.465
cg09471611	-	0.163	0.131	0.358	0.138	0.240	0.061	0.180	0.643
cg11902777	*AHRR*	-0.129	0.265	-0.189	0.437	-0.208	0.207	-0.370	0.171
cg06135139	*BHLHE23*	0.179	0.130	-0.186	0.464	0.176	0.273	0.245	0.677
cg09257526	*IL6R*	-0.159	0.129	-0.273	0.208	-0.286	0.044	-0.447	0.245
cg23973524	*CRTC1*	0.133	0.229	-0.392	0.076	0.414	0.013	0.627	0.093
cg02560388	-	-0.303	0.004	-0.048	0.831	-0.210	0.123	-0.253	0.471
cg06321596	*XYLT1*	-0.325	0.005	-0.243	0.297	-0.013	0.933	0.310	0.452
cg08035323	-	0.146	0.200	-0.177	0.496	0.177	0.214	-0.411	0.402
cg01447828	*PRX*	0.000	0.997	-0.437	0.100	0.448	0.006	-0.478	0.274
cg16619991	*ITGA1*	0.054	0.628	0.291	0.192	-0.058	0.714	-0.487	0.333

cg17426273	*NEBL*	-0.004	0.970	-0.516	0.024	0.120	0.425	0.678	0.118
cg09044186	*APOA5*	0.073	0.544	-0.103	0.701	0.150	0.335	0.343	0.569
cg02304156	*ATP2A1*	0.035	0.745	-0.044	0.852	0.172	0.282	-0.091	0.840
cg25325005	*PLEC1*	0.013	0.912	0.487	0.040	-0.390	0.014	-0.355	0.416
cg19254163	*GPR44*	-0.196	0.059	-0.121	0.572	-0.231	0.064	0.153	0.718
cg01055824	*CCDC88C*	0.000	0.997	0.275	0.246	-0.328	0.037	-0.431	0.388
cg23110422	*ETS2*	-0.188	0.101	0.281	0.257	-0.320	0.052	-0.164	0.777
cg11229399	-	0.050	0.641	0.049	0.831	0.112	0.483	-0.641	0.159
cg27122888	*NRXN2*	-0.229	0.035	-0.150	0.500	-0.211	0.162	0.085	0.852
cg22674699	*HOXD9*	-0.011	0.923	-0.299	0.160	0.210	0.200	0.149	0.774
cg14858469	*NR2F2*	0.072	0.540	-0.402	0.113	-0.030	0.853	-0.280	0.533
cg25853622	*LPP*	-0.096	0.297	-0.218	0.311	-0.146	0.210	-0.129	0.665
cg23572908	*VIPR2*	0.257	0.021	-0.229	0.365	0.269	0.089	0.246	0.644
cg15393221	*PRX*	0.139	0.209	-0.437	0.065	0.288	0.079	0.116	0.840
cg02801786	-	0.084	0.380	-0.134	0.464	0.194	0.195	0.519	0.174
cg07178945	*FGF23*	0.184	0.107	0.236	0.310	0.018	0.909	-0.449	0.246
cg04198308	*FAM19A5*	-0.023	0.836	-0.159	0.464	0.404	0.008	0.229	0.576
cg07324245	*SEPT9*	-0.140	0.151	-0.026	0.906	0.075	0.557	0.344	0.404
cg25114611	*FKBP5*	-0.164	0.109	-0.034	0.870	-0.259	0.072	-0.688	0.199
cg23924887	*ATP8B2*	0.022	0.845	0.178	0.470	-0.253	0.111	0.005	0.991
cg04214430	-	0.043	0.700	0.144	0.562	0.148	0.362	1.155	0.022
cg19719391	-	-0.034	0.743	-0.192	0.371	0.190	0.229	1.072	0.010
cg05396397	*NPPA*	0.126	0.275	-0.377	0.159	0.374	0.026	-0.052	0.876
cg13679772	*FOXN3*	0.099	0.341	0.097	0.672	0.144	0.312	0.370	0.323
cg08591265	*SFRS1*	-0.018	0.868	0.319	0.156	-0.093	0.511	-0.624	0.140
cg15474579	*CDKN1A*	-0.199	0.052	-0.078	0.729	-0.190	0.133	0.036	0.905
cg04551776	*AHRR*	-0.345	0.002	-0.163	0.487	-0.479	0.003	-0.302	0.515
cg20124610	*CARS2*	-0.132	0.238	0.069	0.786	-0.253	0.104	-0.565	0.289

cg11207515	*CNTNAP2*	0.196	0.100	-0.116	0.650	-0.028	0.869	-0.765	0.061
cg16783744	*DPYS*	0.000	0.997	-0.419	0.054	0.140	0.350	0.476	0.311
cg04887172	*CBFA2T3*	-0.147	0.188	-0.285	0.221	-0.047	0.705	-0.005	0.988
cg25949550	*CNTNAP2*	-0.125	0.248	-0.073	0.733	-0.443	0.006	-0.615	0.169
cg09338136	*AHRR*	-0.142	0.203	-0.175	0.449	-0.357	0.026	-0.806	0.053
cg02451831	*KIAA0087*	-0.215	0.056	-0.309	0.183	-0.224	0.172	-0.002	0.996
cg09613161	*COBL*	-0.011	0.917	-0.218	0.314	0.187	0.235	-0.076	0.885
cg02384859	*ARID3B*	0.134	0.212	0.200	0.402	0.242	0.076	0.121	0.762
cg00778858	*MTUS1*	0.093	0.418	-0.009	0.973	0.218	0.129	0.217	0.620
cg22851561	*C14orf43*	-0.127	0.272	-0.148	0.556	-0.063	0.706	-0.068	0.897
cg09022230	*TNRC18*	-0.463	2.68E-05	-0.546	0.013	-0.198	0.166	-0.108	0.707
cg11554391	*AHRR*	-0.255	0.026	-0.150	0.526	-0.408	0.013	-0.410	0.282
cg09373037	*SYT15*	0.165	0.158	-0.249	0.321	0.347	0.034	-0.040	0.939
cg01955533	*CDKN1A*	-0.122	0.215	-0.095	0.672	-0.328	0.019	-0.544	0.179
cg15700587	*MIR548I4*	0.140	0.176	0.034	0.879	0.062	0.652	-0.887	0.030
cg06644428	-	-0.285	0.010	-0.052	0.808	-0.354	0.029	-0.678	0.150
cg09156233	*BMPR1B*	0.267	0.017	-0.131	0.530	0.215	0.176	0.158	0.781
cg20164601	-	-0.126	0.277	0.368	0.136	-0.024	0.890	0.784	0.036
cg08617970	*VARS2*	-0.098	0.410	0.006	0.979	-0.140	0.398	0.565	0.288
cg00073090	-	-0.227	0.028	-0.091	0.653	-0.311	0.040	-0.404	0.334
cg16382047	*GPR55*	-0.184	0.061	-0.110	0.592	-0.354	0.009	0.074	0.825
cg04180046	*MYO1G*	0.010	0.931	-0.144	0.548	0.322	0.045	0.025	0.959
cg24090911	*AHRR*	-0.465	1.81E-05	-0.228	0.293	-0.369	0.013	-0.431	0.352
cg22678402	*FAM125A*	-0.066	0.542	-0.262	0.209	0.104	0.463	0.362	0.411
cg15892280	*ETS2*	-0.134	0.187	-0.150	0.483	-0.102	0.446	0.309	0.486
cg05329352	*ADRA2A*	-0.095	0.377	0.305	0.168	-0.270	0.089	0.676	0.165
cg02945646	*AP1G2*	-0.010	0.920	0.232	0.280	-0.142	0.349	-0.539	0.180
cg01383486	*GINS2*	-0.041	0.714	-0.091	0.699	0.003	0.983	0.009	0.986

cg23673974	*TBKBP1*	0.083	0.463	0.029	0.907	0.060	0.680	-0.259	0.497
cg04211179	*ZBTB17*	0.038	0.723	0.513	0.018	-0.277	0.044	-0.564	0.055
cg20344344	-	-0.032	0.776	0.098	0.684	0.097	0.537	-0.034	0.951
cg18656829	-	-0.104	0.381	-0.557	0.018	-0.080	0.612	-0.203	0.697
cg14316231	*MYST3*	-0.157	0.153	-0.112	0.631	-0.180	0.266	-0.097	0.835
cg04528720	-	0.228	0.032	0.329	0.133	0.242	0.059	0.015	0.953
cg20949306	*RAB3GAP1*	0.073	0.503	-0.050	0.836	0.376	0.005	0.377	0.362
cg17094249	-	-0.074	0.527	-0.059	0.827	-0.137	0.418	-0.208	0.710
cg08129092	*INTS3*	-0.026	0.807	-0.080	0.735	0.160	0.281	-0.245	0.544
cg09858188	-	0.125	0.248	-0.243	0.252	0.035	0.833	0.753	0.104
cg05339037	-	-0.069	0.519	0.098	0.675	-0.226	0.175	-0.199	0.659
cg24859433	-	-0.168	0.139	0.035	0.881	-0.065	0.691	-0.139	0.804
cg02003272	-	0.003	0.976	0.295	0.172	-0.007	0.965	0.081	0.851
cg14624207	*LRP5*	-0.355	7.74E-04	-0.402	0.080	-0.269	0.064	0.312	0.524
cg10351287	*STK32B*	0.070	0.527	-0.308	0.189	0.290	0.051	0.048	0.911
cg25165932	*SELPLG*	0.224	0.059	0.071	0.787	0.250	0.146	0.886	0.070
cg07779120	*IGF1R*	0.244	0.038	-0.123	0.601	0.338	0.035	0.214	0.675
cg20460771	*PTAFR*	0.233	0.021	0.280	0.210	0.336	0.014	0.270	0.438
cg04158878	-	0.101	0.355	0.054	0.824	0.164	0.256	-0.438	0.395
cg13279811	-	-0.205	0.069	-0.028	0.914	-0.097	0.534	0.655	0.154
cg20889322	-	-0.209	0.057	-0.223	0.328	-0.375	0.022	-0.534	0.176
cg20146909	*LRRC8D*	-0.193	0.075	-0.334	0.173	0.014	0.929	0.156	0.773
cg09419102	-	-0.087	0.407	0.107	0.632	-0.084	0.601	0.633	0.200
cg15159987	*CPAMD8*	-0.075	0.499	0.306	0.185	-0.375	0.011	-0.055	0.891
cg14785479	*SCARF2*	0.049	0.636	-0.017	0.930	-0.052	0.747	-0.223	0.616
cg10420527	*LRP5*	-0.306	0.005	-0.319	0.179	-0.292	0.061	0.247	0.563
cg06959340	*JUB*	-0.262	0.024	-0.030	0.900	-0.165	0.328	0.475	0.404
cg14977938	*ZFYVE21*	0.111	0.316	0.063	0.771	0.270	0.103	0.292	0.572

cg02583484	*HNRNPA1*	-0.166	0.123	0.101	0.670	-0.174	0.226	-0.023	0.955
cg03129384	*FAM196A*	0.053	0.605	-0.262	0.198	-0.052	0.744	-0.255	0.514
cg20399616	*BCAT1*	0.348	0.004	-0.137	0.604	0.368	0.028	-0.640	0.105
cg26963277	*KCNQ1OT1*	-0.220	0.054	0.088	0.723	-0.372	0.024	-0.579	0.256
cg23193870	*PTPN6*	-0.170	0.129	0.057	0.818	-0.244	0.120	-0.150	0.778
cg12876356	*GFI1*	-0.254	0.022	-0.370	0.071	-0.229	0.168	0.177	0.707
cg13711966	-	0.078	0.469	-0.048	0.838	0.165	0.279	0.478	0.324
cg03242819	*DOCK1*	0.075	0.503	-0.326	0.129	-0.022	0.894	0.034	0.942
cg18446336	*GNA12*	-0.230	0.030	-0.396	0.086	-0.225	0.124	0.360	0.486
cg05010058	*CEP68*	-0.076	0.500	0.005	0.984	-0.223	0.175	0.791	0.106
cg20664238	*NTRK3*	-0.088	0.446	-0.288	0.212	0.137	0.347	0.209	0.568
cg15342087	-	-0.311	0.008	-0.057	0.813	-0.097	0.558	-0.481	0.367
cg21618017	*RILPL1*	-0.059	0.571	0.083	0.694	-0.238	0.124	-0.180	0.720
cg17489908	*GATA3*	-0.217	0.053	-0.081	0.699	-0.191	0.228	-0.169	0.765
cg13668129	*HNRNPUL1*	-0.113	0.277	0.109	0.612	-0.194	0.194	-0.753	0.044
cg12836863	*BRCA2*	0.097	0.366	-0.104	0.665	0.038	0.789	0.152	0.688
cg03603381	*RASGRP1*	-0.204	0.073	-0.171	0.461	-0.215	0.179	0.221	0.661
cg18630040	*PLA2G7*	0.024	0.816	-0.510	0.011	0.182	0.227	0.207	0.588
cg01765406	-	-0.243	0.020	-0.275	0.216	-0.144	0.294	0.328	0.366
cg13724496	*BMP2*	0.133	0.241	-0.265	0.240	0.193	0.193	-0.099	0.823
cg13990486	*FLJ43663*	0.036	0.749	0.008	0.974	-0.129	0.421	-0.246	0.506
cg05270224	-	0.102	0.348	-0.264	0.218	0.499	0.002	0.493	0.352
cg25310233	-	-0.113	0.293	-0.029	0.897	-0.332	0.037	0.131	0.806
cg24032269	*TCOF1*	0.228	0.023	0.314	0.163	0.146	0.268	-0.131	0.728
cg13774342	-	-0.078	0.503	0.150	0.551	-0.044	0.774	-0.142	0.761
cg18150958	*RPL23A*	-0.085	0.470	0.040	0.863	-0.230	0.164	-0.120	0.838
cg18165852	*CHST13*	0.056	0.631	-0.590	0.022	-0.137	0.393	-0.690	0.105
cg26242531	*ZFYVE21*	0.122	0.226	-0.122	0.584	0.179	0.161	-0.100	0.841

cg12756150	-	0.142	0.208	-0.003	0.990	0.307	0.053	0.198	0.638
cg02150910	*GZMH*	0.137	0.193	0.270	0.239	0.282	0.038	-0.630	0.123
cg21473814	*CRTC1*	-0.029	0.798	-0.397	0.101	0.229	0.135	0.616	0.236
cg00619505	*TMCO3*	0.081	0.451	-0.384	0.101	0.222	0.139	0.817	0.033
cg21188533	*CACNA1D*	0.116	0.302	-0.223	0.334	0.091	0.587	-0.433	0.251
cg13418576	-	-0.138	0.226	0.269	0.282	-0.028	0.863	-0.327	0.464
cg17924476	*AHRR*	0.245	0.022	0.325	0.141	-0.128	0.373	-0.337	0.457
cg02532700	*NCF4*	-0.313	0.006	0.082	0.742	-0.210	0.205	-0.244	0.489
cg12593793	-	-0.224	0.026	0.138	0.540	-0.129	0.402	0.087	0.863
cg14544289	*SPTLC2*	-0.085	0.450	-0.108	0.652	-0.016	0.917	0.215	0.630
cg22619824	*ST7*	-0.345	0.002	-0.319	0.137	-0.092	0.560	0.033	0.947
cg22807449	*HOXB2*	-0.193	0.090	-0.154	0.486	0.028	0.845	0.699	0.154
cg15417641	*CACNA1D*	0.161	0.140	-0.046	0.832	0.109	0.515	-0.808	0.035
cg07381806	*MOBKL2A*	-0.364	0.002	-0.130	0.626	-0.230	0.154	1.342	0.008
cg08126789	*USP10*	0.278	0.011	0.013	0.953	0.215	0.189	0.240	0.667
cg03844971	*MLXIP*	-0.302	0.005	-0.340	0.146	-0.161	0.296	-0.345	0.545
cg05248618	*CA4*	-0.085	0.458	-0.644	0.010	0.139	0.383	0.092	0.799
cg05655806	*CD96*	0.014	0.896	0.378	0.111	0.055	0.720	0.030	0.941
cg09658497	*GNA12*	-0.124	0.275	-0.615	0.011	-0.344	0.033	0.089	0.859
cg06008724	*PHF21B*	0.036	0.724	-0.254	0.239	0.130	0.413	1.028	0.022
cg26348226	*ECE1*	-0.167	0.110	-0.089	0.692	-0.294	0.029	-0.150	0.639
cg18642234	*GPX1*	-0.177	0.098	-0.088	0.698	-0.236	0.128	0.316	0.402
cg19717773	*GNA12*	-0.263	0.024	-0.562	0.026	-0.352	0.030	-0.099	0.847
cg11295113	*FOLR2*	0.109	0.340	-0.026	0.915	0.116	0.459	0.362	0.470
cg01500140	*LIM2*	0.161	0.078	0.016	0.935	0.311	0.025	0.736	0.133
cg15746583	*CD8B*	0.043	0.679	-0.052	0.799	0.006	0.969	-0.532	0.182
cg10416861	-	0.170	0.128	0.240	0.302	0.402	0.011	0.240	0.674
cg09570614	-	0.047	0.650	-0.186	0.394	0.107	0.443	0.031	0.929

cg24687805	*RAB27A*	0.087	0.442	-0.330	0.199	0.235	0.132	0.998	0.013
cg13481776	*ALPI*	0.194	0.060	0.295	0.179	0.233	0.077	0.115	0.665
cg09686308	*CIB3*	0.088	0.443	0.148	0.533	0.217	0.130	-0.381	0.375
cg24996979	*C14orf43*	-0.337	0.001	0.166	0.458	-0.100	0.544	-0.113	0.825
cg03646329	*LPAR6*	-0.077	0.488	0.394	0.072	-0.141	0.369	-0.390	0.373
cg13314145	*NPTX2*	-0.125	0.276	-0.494	0.046	0.108	0.488	0.533	0.249
cg19713429	*CAPZB*	-0.019	0.866	0.254	0.244	-0.105	0.484	0.202	0.605
cg12303084	*ZMYND8*	0.046	0.664	0.457	0.030	-0.163	0.252	-0.024	0.943
cg21752525	-	-0.134	0.223	0.324	0.126	-0.049	0.759	-0.314	0.380
cg15451980	*RORA*	0.200	0.054	0.297	0.217	0.171	0.196	-0.126	0.635
cg15187398	*MOBKL2A*	-0.262	0.012	0.016	0.930	-0.183	0.272	0.664	0.248
cg00326958	*HNRNPF*	-0.075	0.522	0.484	0.049	-0.214	0.165	-0.294	0.479
cg24996482	-	0.137	0.215	-0.319	0.139	0.239	0.128	0.226	0.624
cg05007126	*SDCBP2*	-0.026	0.811	0.225	0.386	-0.323	0.022	0.421	0.304
cg26958735	-	0.141	0.194	-0.232	0.306	0.079	0.620	-0.275	0.547
cg03172931	-	0.243	0.023	0.192	0.410	0.213	0.148	0.229	0.586
cg15554421	*C3orf26*	-0.204	0.055	-0.369	0.101	-0.292	0.049	-0.004	0.993
cg11485823	*DPCR1*	-0.015	0.890	0.164	0.497	0.153	0.321	-0.181	0.640
cg24741609	*GLIS1*	-0.098	0.398	0.124	0.581	-0.218	0.172	-0.503	0.281
cg10179300	*TRIO*	-0.231	0.034	0.149	0.556	-0.272	0.100	0.087	0.860
cg02352716	-	0.226	0.032	0.250	0.277	0.186	0.156	0.275	0.177
cg09747445	*TLE3*	-0.190	0.086	-0.149	0.491	-0.371	0.012	-0.152	0.698
cg02869235	-	0.006	0.957	-0.093	0.693	-0.055	0.706	-0.388	0.349
cg22574825	*FLT1*	0.114	0.304	-0.114	0.605	-0.073	0.665	0.167	0.644
cg13185177	*GP5*	-0.162	0.115	-0.330	0.137	0.211	0.143	0.333	0.407
cg22851200	*TRIP6*	-0.192	0.078	-0.324	0.166	0.012	0.943	0.731	0.100
cg15207742	*RIMS4*	-0.045	0.675	-0.428	0.049	0.163	0.323	-0.093	0.850
cg14074174	*SNAPC2*	-0.147	0.152	-0.138	0.446	-0.222	0.110	-0.794	0.064

cg16611234	-	-0.266	0.025	-0.195	0.455	-0.400	0.016	0.001	0.997
cg26361535	*ZC3H3*	-0.089	0.416	-0.008	0.972	-0.209	0.194	0.209	0.625
cg23867146	-	0.038	0.727	0.103	0.657	-0.134	0.403	1.004	0.004
cg11621113	*MORG1*	-0.060	0.585	0.510	0.019	-0.119	0.417	0.221	0.444
cg07069636	-	-0.048	0.640	0.272	0.200	-0.202	0.091	-0.453	0.155
cg12884422	-	-0.199	0.093	-0.030	0.909	-0.090	0.597	0.497	0.299
cg07986378	*ETV6*	-0.347	8.64E-04	-0.002	0.994	-0.011	0.943	0.180	0.715
cg26364091	*CHADL*	-0.320	0.004	-0.177	0.429	-0.199	0.239	0.530	0.298
cg12158535	*PACS2*	0.084	0.465	-0.152	0.509	0.156	0.335	0.553	0.273
cg21322436	*CNTNAP2*	-0.460	2.81E-05	-0.555	0.009	-0.358	0.025	-0.124	0.810
cg10126923	*NKG7*	0.066	0.523	-0.209	0.378	0.285	0.054	-0.207	0.616
cg10951873	*RUNX3*	-0.078	0.498	0.321	0.147	-0.137	0.376	0.068	0.869
cg05284742	*ITPK1*	-0.406	1.33E-04	-0.456	0.032	-0.089	0.554	0.370	0.266
cg12873476	-	-0.263	0.010	-0.228	0.262	-0.346	0.036	-0.498	0.340
cg21733502	*ZSCAN5B*	-0.307	0.005	-0.022	0.928	-0.131	0.425	0.226	0.665
cg01294327	*LINGO3*	-0.319	0.006	0.079	0.754	0.006	0.972	0.650	0.055
cg15380836	*RILP*	-0.135	0.173	0.190	0.364	-0.140	0.352	-0.414	0.410
cg06419750	*GLT1D1*	0.025	0.822	-0.134	0.556	-0.004	0.982	-0.438	0.154
cg00336149	*CACNA1D*	0.138	0.221	-0.229	0.331	0.194	0.253	-0.931	0.004
cg22777952	*FOXB1*	0.011	0.923	-0.637	0.004	0.044	0.777	0.032	0.946
cg14667406	*LCT*	-0.303	0.009	-0.690	0.008	-0.014	0.929	-0.334	0.512
cg10204884	*PCBP3*	-0.120	0.244	-0.116	0.594	-0.203	0.210	-0.385	0.507
cg12916723	*NKG7*	0.197	0.083	-0.038	0.877	0.245	0.088	0.279	0.427
cg01726890	-	0.214	0.051	0.121	0.605	0.150	0.277	-0.194	0.580
cg03877174	*KIF1C*	0.150	0.138	0.011	0.961	0.065	0.682	-0.286	0.513
cg05635807	-	0.135	0.253	-0.680	0.007	0.313	0.050	-0.043	0.937
cg19956914	*SUMF2*	0.078	0.491	0.016	0.950	0.394	0.015	0.499	0.251
cg10908953	*SORL1*	-0.178	0.085	-0.151	0.481	-0.170	0.262	-0.687	0.167

cg14096889	*ADAMTSL3*	0.112	0.331	-0.553	0.013	0.214	0.165	0.470	0.180
cg23351584	*PRSS23*	-0.203	0.081	-0.166	0.505	-0.442	0.005	-0.351	0.354
cg13389508	*PLEC1*	-0.164	0.132	-0.136	0.532	-0.337	0.037	-0.645	0.203
cg09762515	*CUX1*	0.083	0.432	0.307	0.177	0.223	0.137	0.527	0.172
cg16145216	*HIVEP3*	0.280	0.014	0.309	0.193	0.178	0.242	0.125	0.579
cg04039397	*CD96*	-0.196	0.081	0.320	0.164	0.061	0.695	-0.268	0.416
cg02818189	-	-0.132	0.163	-0.019	0.927	-0.164	0.239	-0.037	0.937
cg09301294	*EPHA6*	0.011	0.913	-0.428	0.026	0.069	0.666	0.559	0.307
cg25260137	-	-0.029	0.799	-0.630	0.006	0.067	0.668	0.162	0.701
cg05228408	*CLCN6*	-0.103	0.332	0.012	0.952	-0.120	0.391	-0.175	0.737
cg02985540	-	-0.146	0.159	0.002	0.993	-0.230	0.123	-0.273	0.482
cg26971042	*TLE3*	-0.288	0.010	-0.319	0.181	-0.220	0.179	-0.502	0.252
cg06868100	*PRR15*	-0.120	0.291	0.428	0.082	0.037	0.828	0.874	0.088
cg19827923	*GPR55*	-0.114	0.302	-0.247	0.298	-0.380	0.013	-0.436	0.362
cg14580211	*C5orf62*	-0.348	5.12E-04	-0.221	0.352	-0.214	0.128	0.382	0.429
cg07827420	*SEPT9*	-0.135	0.251	0.180	0.480	-0.113	0.492	0.549	0.308
cg01107178	*ANKRD11*	0.195	0.069	0.376	0.114	0.172	0.212	-0.468	0.125
cg14588779	*AKAP8L*	-0.088	0.425	0.129	0.571	-0.185	0.239	-0.167	0.619
cg05460226	*PIK3R5*	-0.251	0.023	-0.035	0.869	-0.169	0.285	0.486	0.349
cg14428590	*FSIP1*	-0.230	0.034	-0.078	0.751	0.024	0.869	-0.254	0.454
cg26736540	*TFAP2C*	0.030	0.794	-0.397	0.128	0.004	0.981	-0.540	0.172
cg01564343	*TREML1*	-0.046	0.668	-0.253	0.273	0.111	0.480	-0.127	0.767
cg13193840	-	-0.144	0.221	0.345	0.155	-0.156	0.349	0.488	0.398
cg13039251	*PDZD2*	0.208	0.053	-0.381	0.092	-0.134	0.357	-0.151	0.563
cg06901890	*FNBP1*	-0.129	0.271	0.283	0.233	-0.138	0.410	0.433	0.360
cg17287155	*AHRR*	-0.194	0.091	0.103	0.623	-0.329	0.037	-0.315	0.424
cg20303561	*CCDC88C*	-0.005	0.961	0.422	0.051	0.017	0.916	-0.041	0.925
cg03760919	*RUNX1T1*	0.184	0.089	0.501	0.025	-0.020	0.877	-0.497	0.181

cg04755561	*PKMYT1*	0.048	0.643	0.022	0.921	-0.071	0.607	0.729	0.063
cg04105282	*CRTAC1*	0.082	0.460	-0.372	0.107	0.197	0.238	0.415	0.451
cg08548559	*PIK3IP1*	0.018	0.874	0.227	0.346	-0.391	0.012	-0.288	0.506
cg16556677	*KCNQ1OT1*	-0.179	0.113	0.115	0.652	-0.241	0.138	-0.180	0.712
cg06009448	*C7orf50*	-0.167	0.095	-0.071	0.746	-0.120	0.381	-0.095	0.827
cg19784816	*ITIH1*	0.054	0.598	-0.221	0.317	0.280	0.081	0.543	0.180
cg00378510	*LINGO3*	-0.197	0.073	0.279	0.202	0.121	0.452	0.949	0.021
cg08709672	*AVPR1B*	-0.270	0.022	-0.059	0.815	-0.291	0.066	-0.587	0.230
cg19838043	*ZFYVE21*	0.028	0.796	-0.299	0.208	0.327	0.043	0.978	0.038
cg07123182	*KCNQ1OT1*	-0.358	0.002	-0.132	0.592	-0.439	0.009	-0.111	0.840
cg18387156	*NRG1*	0.024	0.825	-0.285	0.203	-0.069	0.672	-0.210	0.711
cg01416295	*MRPL24*	-0.084	0.446	0.284	0.245	0.106	0.495	-0.749	0.062
cg01598741	*HMGA2*	-0.203	0.070	-0.273	0.274	0.164	0.319	0.176	0.683
cg20185017	*CACNB2*	0.002	0.984	-0.122	0.526	0.008	0.958	-0.115	0.841
cg25922751	*NCOR2*	-0.205	0.060	0.121	0.606	0.141	0.303	-0.017	0.965
cg07066369	*CCND2*	-0.226	0.050	-0.263	0.276	-0.353	0.033	-0.037	0.933

[a] Estimates were calculated by linear regression models. Methylation values were inverse-normal transformed and concentrations of urinary 2-hydroxynaphthalene and 1-hydroxypyrene were ln-transformed. [b] Adjusting for age, drinking status, BMI, occupation, beadchip operation date, differential leukocyte proportions and geographical regions. [c] Adjusting for 1-hydroxypyrene, age, drinking status, BMI, beadchip operation date, differential leukocyte proportions.

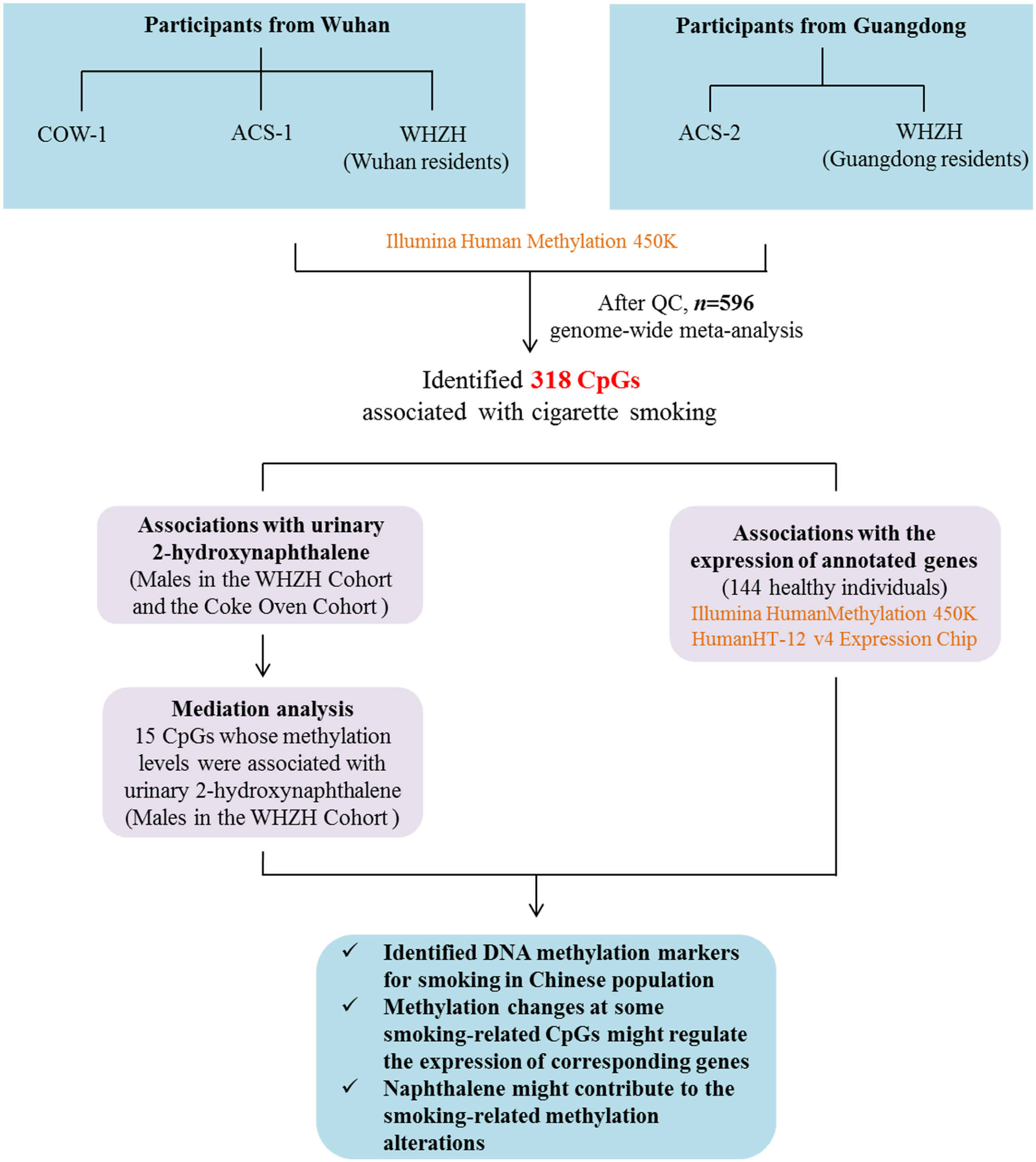

Figure S1. The flowchart of the study.

References

Dogan MV, Shields B, Cutrona C, Gao L, Gibbons FX, Simons R, et al. 2014. The effect of smoking on DNA methylation of peripheral blood mononuclear cells from african american women. BMC Genomics 15:151; doi: 10.1186/1471-2164-15-151 [Online 3 February 2014].

Guida F, Sandanger TM, Castagne R, Campanella G, Polidoro S, Palli D, et al. 2015. Dynamics of smoking-induced genome-wide methylation changes with time since smoking cessation. Hum Mol Genet 24:2349-2359.

Philibert RA, Beach SR, Lei MK, Brody GH. 2013. Changes in DNA methylation at the aryl hydrocarbon receptor repressor may be a new biomarker for smoking. Clin Epigenetics 5:19; doi: 10.1186/1868-7083-5-19. [Online 10 February 2014].

Shenker NS, Polidoro S, van Veldhoven K, Sacerdote C, Ricceri F, Birrell MA, et al. 2013. Epigenome-wide association study in the European Prospective Investigation into Cancer and Nutrition (EPIC-Turin) identifies novel genetic loci associated with smoking. Hum Mol Genet 22:843-851.

Sun YV, Smith AK, Conneely KN, Chang Q, Li W, Lazarus A, et al. 2013. Epigenomic association analysis identifies smoking-related DNA methylation sites in African Americans. Hum Genet 132:1027-1037.

Zeilinger S, Kuhnel B, Klopp N, Baurecht H, Kleinschmidt A, Gieger C, et al. 2013. Tobacco smoking leads to extensive genome-wide changes in DNA methylation. PLoS One 8:e63812; doi:10.1371/journal.pone.0063812 [Online 17 May 2013].
